# Floristic composition and turnover analysis in Dahomey Gap and the surrounding sub‐humid Togolese mountain minor forest refuges: Importance for biogeography and biodiversity conservation in sub‐Saharan Africa

**DOI:** 10.1002/ece3.9304

**Published:** 2022-10-04

**Authors:** Kossi Adjossou, Kouami Kokou, Marc Deconchat

**Affiliations:** ^1^ Département de Botanique, Laboratoire de la Recherche Forestière, Faculté des Sciences Université de Lomé Lomé Togo; ^2^ Dynafor, INRAE Université de Toulouse Castanet‐Tolosan France

**Keywords:** Dahomey Gap (DG), floristic approach, forest refuges, Togolese mountain, tropical biodiversity, vegetation

## Abstract

The origin of Dahomey Gap (DG) flora is one of the central biogeographical questions in sub‐Saharan, which has been addressed in several studies. However, floristic evidence based on representative samples from the DG seems to be lacking in the scientific debate. The present study was conducted to fill this gap. Specifically, we assessed Togolese mountain riparian forests as minor forest refugia, examined their contribution to larger sub‐Saharan forest refugia, and discussed the significance of these findings for biogeography and biodiversity conservation. Southwest Togo, West Africa, and sub‐Saharan Upper Guinea Region Floristic data were collected in riparian forests through an intensive botanical inventory (*n* = 198; 50 × 10 m^2^). A comparative analysis was performed based on the floristic evidence related to forest refuges. The results showed significantly high species richness (868 species) and a high gamma and beta diversity associated with spatial turnover patterns. They also showed a high affinity between the study forests and large sub‐Saharan forest refugia. Riparian forests share about 60% of their current species richness with large sub‐Saharan forest refugia and contained refuge bio‐indicator species. The floristic evidence, consistent with those of other studies, suggests that Togolese mountains would have very ancient origins and have experienced paleoclimatic events. The studied riparian would have served as refuges during recurrent climatic episodes. Our results support the minimal forest fragmentation hypothesis (network of refugia along rivers). However, they seem to be incompatible with the idea that the DG flora may be essentially a relic of the early Holocene. In sub‐Saharan Africa, where maintaining a vast area of natural forest is difficult due to human pressure, efforts to preserve maximum species diversity should include a focus on the conservation of minor forest refuges, particularly in sub‐humid mountain riparian zone.

## Graphical abstract

The origin of the DG is one of the central biogeographical questions in sub‐Saharan Africa, which has been addressed in recent phylogenetic studies. Till date, no study based on large floristic data collected in the DG has addressed this question. Our work, based on large floristic data collected in the DG and compared with those of regional floras, is consistent with the recurrent opening of the DG and suggests that during the warm period, the riparian forests of the sub‐humid Togolese Mountains would have served as important minor forest refugia and would continue to play this role in the future climate change.

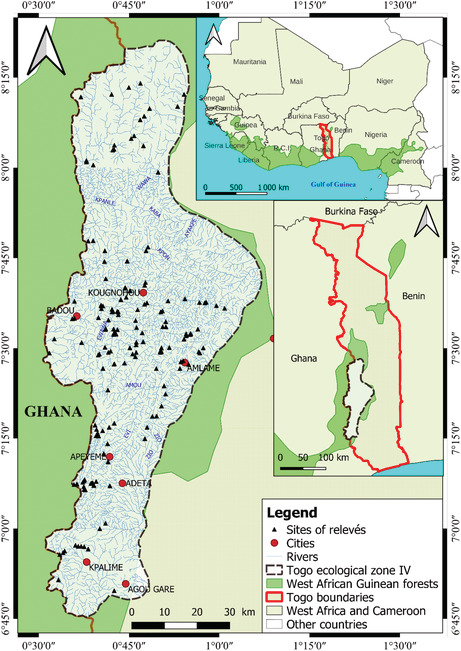

## 1 INTRODUCTION

Forest refugia are crucial as retreats for many plant and animal species, which would have become extinct during the past climate change coinciding with the glacial cycles (Broecker & Denton, 1989; Husemann et al., 2014; Maley, 1980). They have played an important role in structuring the biotic and genetic diversity of these organisms (Carlson et al., 2012; Ley et al., 2016; Nicolas et al., 2010). According to ecologists, forest refugia can again function similarly during current and future climate change and are a high conservation priority as key areas for the long‐term persistence of species and genetic diversity (Médail & Diadema, 2009; Montade et al., 2014). Hence, it is important to identify ancient climate refugia where possible and protect them at multiple scales (Eeley et al., 1999 cited in Noss (2001)).

Forest refuges occur at different spatial scales. Minor forest refugia are small‐scale climatic refugia as defined by Noss (2001). In other words, forest refuges occupy small sites such as forest pockets, local river catchments, micro‐environments, riparian habitats, mountain valleys, and foothills. Numerous studies worldwide have revealed that these minor forest refuges are very important as key areas for the long‐term persistence of species and genetic diversity (Aide & Rivera, 1998; Carnaval & Moritz, 2008; Cheddadi et al., 2009; Dupont et al., 2009; Ehrich et al., 2008; Jennings et al., 2011; Jiménez‐Mejías et al., 2012; Kuneš et al., 2008; Selwood & Zimmern, 2020; Terral et al., 2004; Tibby et al., 2017; Pennington et al., 2000).

Despite the importance of minor forest refuges for their impact on maintaining current and future diverse gene pools, they have received little attention in sub‐Saharan Africa (Wieringa & Poorter, 2004), probably due to the previous perception of forest refugia in this area. It has long been suggested that in the late Quaternary Period, the African rainforests were restricted to several large refugia (Maley, 1989; Maley & Brenac, 1998). Moreover, the sub‐Saharan African refugia located south of the equator are highly developed, while those located north of the equator are rather smaller (Linder, 2001). However, recent phylogenetic studies in sub‐Saharan Africa areas have contradicted these ideas by supporting that there are many small, rather than a few large, forest refugia in tropical Africa (Bohoussou et al., 2015).

In sub‐Saharan Africa, the Pleistocene forest refugia hypothesis is supported by palynological, climatological, and phylogenetic evidences. Major climatic refugia would probably have been located near Cape Palmas and the Nimba Mountains in Liberia, Cape Three Points in Ghana (Wieringa & Poorter, 2004), Benin‐Ghana, southwest Cameroon, southern Gabon, northern Gabon, eastern Democratic Republic of Congo, and western Uganda (Maley, 1989; Nicolas et al., 2010). However, minor refugia have not been identified elsewhere and studied in terms of biogeography and biodiversity conservation. Few studies on sub‐Saharan tropical landscapes, however, locate and assess the contribution of minor forest refugia to the distribution and conservation of local or regional plant diversity (Hall, 1973, 1981). To the best of our knowledge, the only case studies on the biogeography of minor forest refugia focus on the “ecological islands of south‐eastern Nigeria” and the southern slopes of Mount Cameroon (Hall, 1973, 1981). Based on the floristic evidence, Hall (1981) has showed that the ecological islands of south‐eastern Nigeria served as a rainforest refuge during the Pleistocene climate change. This approach also allowed him to estimate the minimum size of a viable conservation area at 10 km^2^, for the purposes of conserving floristic diversity in the small Pleistocene refugial forests of Nigeria.

The hypothesis that riparian forests acted as refugia for flora in sub‐humid tropical landscapes covered by savannah or deciduous forest during the arid Pleistocene period has been proposed to explain the relationships between the species composition of riparian and upland (i.e., non‐riparian) forests. In addition, it was postulated that tropical forests re‐expanded rapidly in the early Holocene originated from these refugia. Evidence for these assumptions has also been demonstrated in the temperate forests and in the Neotropics forests (Aide & Rivera, 1998; Meave & Kellman, 1994; Silva de Miranda et al., 2018). According to Meave and Kellman (1994), if the significant number of upland rainforest species can survive in these riparian forests, they could provide a plausible explanation for the survival of regional forest biotas during drier Pleistocene phases and their rapid reappearance in the Holocene pollen record. However, such studies have not been conducted in sub‐Saharan Africa. Although riparian forests are frequently studied in sub‐Saharan tropical Africa, much less information is available on their biogeography, particularly in mountain areas. Previous studies were conducted in South Africa (Hood & Naiman, 2000), Benin (Natta, 2003), Togo (Adjossou, 2004; Kokou et al., 2008), Central Africa (Gautier‐Hion & Brugiere, 2005), the Volta Sub‐Basin of Ghana (Boakye et al., 2019), Southern Sierra Leone (Fayiah et al., 2020), and South East Kenya (Habel & Ulrich, 2021) have shown that riparian areas are generally richer than other forest ecosystems. However, the floristic similarity between riparian and “Terre firme” forests has not been investigated to estimate the proportion of regional humid relict species in riparian forests, thus allowing the discussion of their status as forest refugia.

The biogeography and history of the Guinean‐Congolian rainforest have received a great deal of attention. White (1979, 1983) defined the Guinean‐Congolian rainforest as a phytogeographic domain comprising about 8000 plant species, of which 80% are endemic. These forests have been subdivided into three sub‐centers of endemism (Fayolle et al., 2014; Hardy et al., 2013; Linder et al., 2012; Poorter et al., 2004): (1) the Lower Guinea forests, which extend from Nigeria to the eastern border of Gabon—coinciding with the separation of the Congo and Ogooué basins, (2) the Congolese forests properly so called, which are confined to the Congo watershed and (3) the Upper Guinea forests, stretching from Ghana to Guinea and separated from the other entities by Dahomey Gap, the savannah band in Togo and Benin. The origin of the biogeographical feature of African rainforests has been the subject of interesting literature reviews (Hardy et al., 2013; Poorter et al., 2004). According to the authors, about 2.4 million years ago, the polar ice caps expanded to the point where they extended into temperate zones. Cold and temperate periods thus followed one another according to the periodic oscillations of the Earth in its orbit around the sun (Milankovitch cycle). Warming occurred 16,000 years ago, which caused our current climate. The ice ages changed the spatial arrangement of the climate zones and thus, influenced the environmental conditions. Species could either follow the new organization of the climate zones, adapt to the new conditions, or become extinct. These ice ages caused a great drought in the African continent. Tropical forests survived in a few refuges, which impoverished the tropical forest flora. About 6000 years ago, rainforest occupied a much larger area than presently and extended across the Dahomey Gap, thus connecting Upper and Lower Guinea (Maley, 1980, 1997).

So, the biogeographic history of sub‐Saharan tropical Africa is rich, especially around the Dahomey Gap. The origin of the DG flora is one of the central biogeographic questions in sub‐Saharan Africa, which has been addressed in recent phylogenetic and floristic studies (Demenou et al., 2016; Duminil et al., 2015; Fayolle et al., 2014; Koffi et al., 2011; Linder et al., 2012; Marshall et al., 2021). Phylogeographic data suggest that the forest flora of the DG may be essentially relics of the early Holocene when the geographical distribution of the Guinean‐Congolian forest reached its maximum (Demenou et al., 2016; Duminil et al., 2015; Koffi et al., 2011). However, the floristic evidence for these conclusions, based on representative samples from the DG, seems to be absent from previous reports on this topic. It is true that there is floristic evidence based on regional data that has allowed discussion of the biogeography of sub‐Saharan Africa as a whole (Fayolle et al., 2014; Linder et al., 2012). But these data were not representative of the flora of the Dahomey Gap, as the plots sampled were selected elsewhere across the forest blok. A very few plots (1 or 2) were selected in the DG plain. This was also true for some species used in phylogenetic studies such as *Distemonanthus benthamianus* (Demenou et al., 2016), which were not from the Dahomey gap but from the Togo mountains. Till date, no study based on large floristic data collected in the DG has addressed biogeographical issues. Phylogeographic and genetic data should therefore be compared to those of the representative floristic data, taken in and around the DG, to prove their value in understanding the biogeographic history of the Dahomey Gap.

The mountains of Togo form the main mountainous area adjacent to the DG in sub‐Saharan Africa. Due to this position, the vegetation of the Togolese Mountains is often described as dry vegetation similar to that of the DG (Hall & Swaine, 1976). The vegetation of the Togolese Mountains has been the subject of several botanical surveys (Aké Assi, 1971; Akpagana, 1989, 1992; Brunel, 1977, 1978; Guelly, 1994). Nevertheless, there is a significant lack of information specifically concerning the riparian forests of the region, in which new species of Togolese flora have recently been discovered (Adjossou, 2004, 2009; Adjossou & Kokou, 2009; Kokou, 1998; Kokou et al., 2008). Although several studies have focused on the vegetation of the Togolese Mountains, little information on this vegetation has been published in international journals (Adjossou & Kokou, 2009; Akpagana, 1992; Kokou et al., 2008); consequently, information on this vegetation is not easily accessible to a wide scientific audience.

We test the hypotheses that (1) during the opening of the DG, the riparian zones of the Togolese mountains would have served as a mini‐refuge for forest flora during restrictive climatic regimes; the populations persisting in these zones would have recolonized the surrounding landscape when conditions favorable to their survival and reproduction returned; (2) the DG would have been subjected to repeated openings (in the Pleistocene and then in the Holocene); during these crisis periods, forest fragmentation would have been minimal or would have taken the form of a network of refuges (along rivers and/or in the mountains) where most species would have survived; (3) the forest flora of the DG could be essentially relics of the early Holocene period when the geographical distribution of Guinean‐Congolian forest reached its maximum (Demenou et al., 2016; Duminil et al., 2015; Koffi et al., 2011).

We aimed to comparatively study the flora of the Togolese Mountains riparian and those of the large tropical climatic forest refuges. Specific objectives were to assess Togolese riparian as minor forest refugia, examine their contribution to larger sub‐Saharan forest refugia and discuss the importance of these results for biogeography and biodiversity conservation in sub‐Saharan tropical Africa.

We base our study on the floristic evidence and attributes commonly used in the field of biogeography. According to the ecologists and biogeographers, areas that have served as historical refugia during restrictive climatic regimes currently harbor higher levels of biodiversity than areas that have not experienced refugia (Boyer et al., 2016; Russell et al., 2009). They are also characterized by a high incidence of rare species, and a high level of beta and gamma diversity associated with species turnover (Cowling & Lombard, 2002). These are complex areas with a large number of endemic species, typical species of many forest types (Do Prado et al., 2014; Fiaschi & Pirani, 2009), and a significant proportion of tropical rainforest species (Meave et al., 1991; Meave & Kellman, 1994). These areas contain bio‐indicator taxa of rainforest refugia (Blan, 1993; Tchouto et al., 2009) and share similar vegetation (White, 1983). All these floristic evidence were evaluated and compared with those of other studies (palaeobotanical, palynological, phylogeographical, and geological).

## 2 MATERIALS AND METHODS

### 2.1 Study area

**FIGURE 1:**
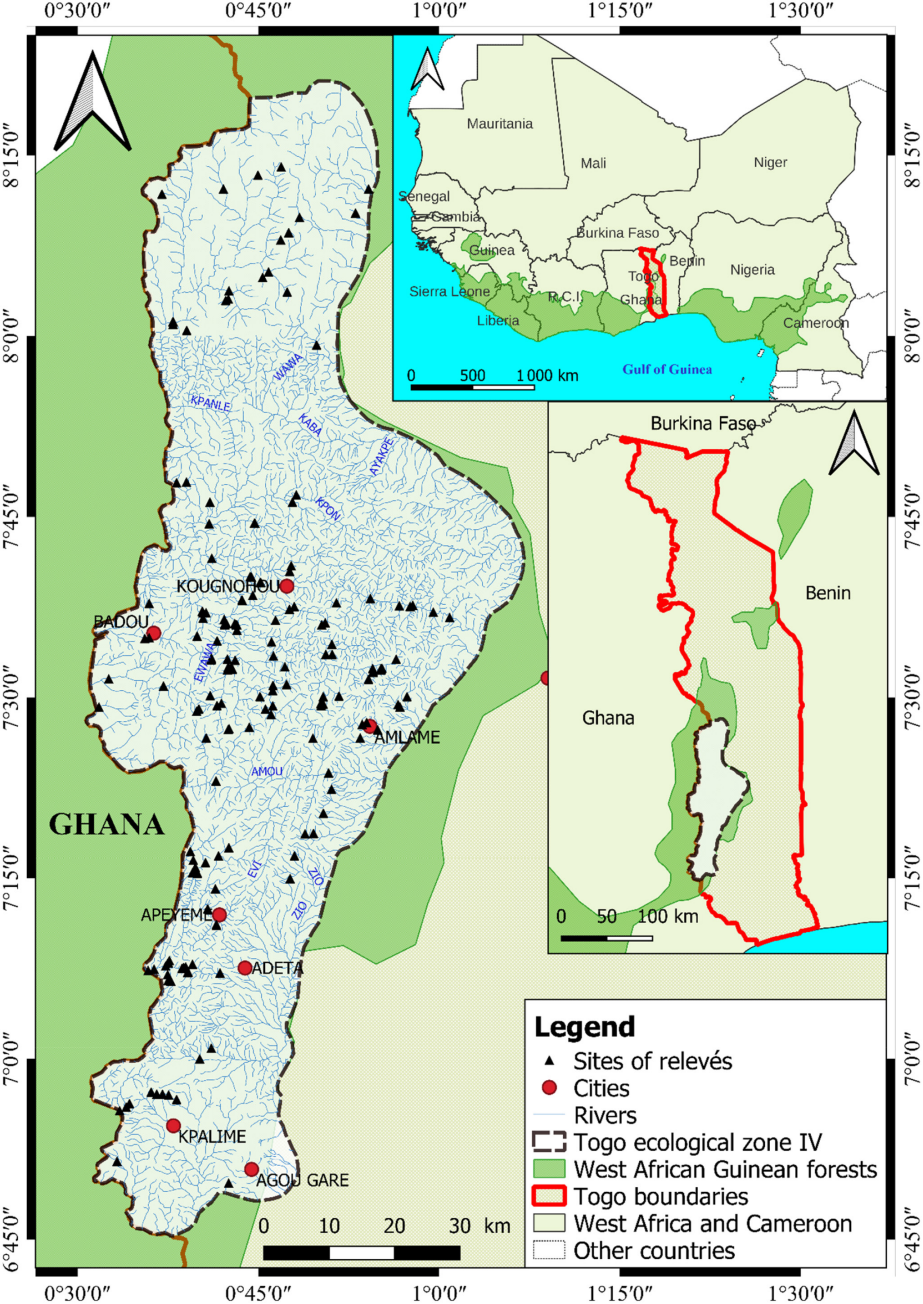
Map showing the hydrographic network of Togo highlands and the location of the 198 study plots.

The study area (6°15′–8°20′N; 0°30′–1°E) covers 6441 km^2^ in the southern part of the Atakora Mountains, southwest Togo, on the border between Togo and Ghana (Figure 1). It is adjacent to the DG, an extension of the savannah woodlands from the Sahel to the Gulf of Guinea, which separates the Upper Guinean forests from the rest of the African rainforests (Poorter et al., 2004). The climate in this area is mountainous (Papadakis, 1966) and characterized by a long rainy season (8–10 months). The average annual temperature and total annual rainfall ranges are 21–25°C and 1250–1900 mm, respectively. The study area presents a strong topographic and geomorphological heterogeneity, which is named Togo Mountains, Togo Monts, or Togo Highlands. The Togo Mountains are formed by a succession of plateaus (Kloto, Kouma, Danyi, Akposso, Akébou, and Adélé plateaus), mountains, hills, valleys, and deep caves, with average 800 m altitude and relatively high peaks in places, such as Djogadjèto (972 m) and Liva (950 m). The main geological component is late Precambrian. It consists of quartzites, phyllites, shales of Togo, and Buem sandstones that were extensively folded and metamorphosed during the Pan‐African Cambrian orogeny (Hall & Swaine, 1976). The main edaphic component is shale. A complex network of secondary rivers covers the area with three catchments: the Lake Volta basin to the west of the Monts and the Mono and Zio basins to the east of the Monts. The study area is mainly covered by semideciduous forests interspersed with Guinean savannah. Six semideciduous montane forestries were identified: Sterculiaceae and Sapotaceae forest, *Celtis mildbraedii* forest, *Terminalia superba* forest, *Ricinodendron heudelotii* forest, Meliaceae and Moraceae forest, and *Parinari excelsa* forest (Akpagana, 1989).

For the past four decades, the forests in the Togolese Mountains have been affected by agriculture, particularly coffee and cocoa production, which has led to repeated clearing. Charcoal production by local people in the 1990s, after the reduction in coffee and cocoa prices, has tended to aggravate the situation. Thus, the remaining forest is highly fragmented today and mainly located in hard‐to‐reach areas and along rivers (Adjossou, 2004). The high population growth rate in Togo (3.3% per year) also affects the forest remnants, in contrast to those along the rivers, which are still relatively untouched by the local population partly for traditional reasons.

### 2.2 Site selection and plant survey

A small‐scale topographic map (1:200,000) available for the study area was used to identify the hydrological network and potential sites with riparian forests for selecting the sampling sites. The sites were selected according to a general strategy to cover the habitat diversity (Mueller‐Dombois & Ellenberg, 1974). However, sites located on steep slopes, which are very difficult to access, were not selected (Figure 1). The floristic inventory was based on the plots méthod (*n* = 198; 50 × 10 m, or 250 m^2^ (50 × 5 m) on either side of the riverbed [The area of 250 m^2^ is used when, due to clearing, the width of the forest is less than 10 m]) carried out in the selected riparian communities during the dry season between 2002 and 2007. This plot size is in line with what is usually used in riparian forest inventories in West Africa (Kokou, 1998; Natta, 2003). Following the riverbed, usually upstream, the survey plots were located using GPS. At each site, the presence/absence of vascular plant taxa (trees, shrubs, lianas, herbs, and epiphytes) was noted. To have a complete floristic composition, these surveys were completed by including the species not observed in the 198 samples but observed in the riparian vegetation during the inventory. Taxon identification in the field is based on the floras reported by Aubréville (1959), Brunel et al. (1984), and Hutchinson et al. (1954–1972). Specimens of species that could not be identified in the field were collected and identified later at the herbarium of the University of Lomé (Togo). To standardize the species names, the African plant database was used.

### 2.3 Data analysis

#### 2.3.1 Floristic diversity analysis

##### Species and taxonomy richness

Species richness is the number of species recorded for a specific group of organisms during a given period. To calculate the species richness of the riparian forests of the Togolese Mountains, a general floristic list was established by compiling the inventories of the 198 floristic surveys, completed by the species not identified in the 198 surveys but observed in the riparian forests during the field surveys. However, only the species recorded in the plots were used in the statistical analyses. The inventory of the accepted species was based on those reported by Aubréville (1959), Brunel et al. (1984), and Hutchinson et al. (1954–1972). To avoid duplication, the accepted species were listed alphabetically. The synonyms for each species were checked using the African plant database. The total number of species on this general list was taken as the species richness. Then, the resulting species richness was compared to those of the richest sites in sub‐Saharan Africa considered as forest refuges, such as the Ziama forest in south‐eastern Guinea, the Taï forest in south‐eastern Côte d'Ivoire, the closed forests in southern Ghana, the Monts Doudou forests in southwestern Gabon, the Campo‐Ma'an forests in southwestern Cameroon, etc. (Table 1). To calculate taxonomic richness, the species recorded were grouped according to their taxonomic level (such as genus and family). Within each group, the species richness was calculated. The floristic list of the riparian forests of Monts Togo was prepared according to the APGIII classification system to compare it with the available floristic lists on the vegetation of sub‐Saharan Africa, mainly established according to the APGIII classification system (Fayolle et al., 2014; African plant database https://www.ville‐ge.ch/musinfo/bd/cjb/africa/index.php?langue=an).

**TABLE 1:** Country, geographical zone, refuge forest sites, area, annual rainfall, subsol, and taxa comparison between riparian forests and sites considered as forest refuges throughout tropics Africa region.

Country	Geographical zone	Refuge forest sites	Forest type	Area (ha)	Annual rainfall	Subsol	Taxa	Sources
Guinée	Southeast	Ziama	E‐SD	116,170	1600–2000	Granite	1262	Wright et al. (2006)
Côte d'Ivoire	Center‐East	Bossématié	SD	22,200	1400–1500	Schist	611	Kouamé et al. (2004)
	Southeast	Mabi	E	59,616	1650–1700	Schist	640	Kouamé et al. (2004)
		Songan	E	38,189	1600–1650	Schist	591	Kouamé et al. (2004)
		Tamin	E	24,934	1650–1700	Schist	512	Kouamé et al. (2004)
		Yaya	E	23,877	1700–1800	Schist	617	Kouamé et al. (2004)
	South coast	Dassioko	E	11,203	1550–1600	Sand	719	Kouamé et al. (2004)
		Monogaga	E	39,660	1650–1750	Sand	859	Kouamé et al. (2004)^a^
		Port Gauthier	E	2590	1550–1600	Sand	705	Kouamé et al. (2004)^a^
		Banco	E	3300	2000	Sand	773	Kouamé et al. (2004)^a^
	Center‐West	Haut Sassandra	SD	102,400	1460–1680	Granite	843	Kouamé et al. (2004)^a^
		Marahoué	SD	101,000	1400	Schist	475	Kouamé et al. (2004)^a^
	Southwest	Haute Dodo	E	236,733	1900–2300	Granite	906	Kouamé et al. (2004)^a^
		Taï	E	300,000	1800–2200	Granite	849	Kouamé et al. (2004)^a^
Ghana	South		E‐SD	‐	1750	Schist	1248	Hall and Swaine (1976)
Togo	Southwest	Togo mountain	SD (Terre ferme)	‐	1500–1900	Schist	648	Akpagana (1992)
		Togo mountain	RF	‐	1500–1900	Schist	868	This study
	South coast	Coast plain	SD	‐	1000–1250	Sand	649	Kokou et al. (1999)
Benin	‐	Sudano‐guinean	RF	‐	‐	‐	556	Natta (2003)
	‐	Soudanean	RF	‐	‐	‐	591	Natta (2003)
Gabon	Southwest	Monts Doudou		332,000			991	Sosef et al. (2004)
Cameroon	South	Campo‐Ma'an	E‐SD	770,000	1670–2950	‐	2297	Tchouto et al. (2009)

*Note*: The total number of species recorded in our study is of the same order as those of sub‐Saharan Africa forest refuges.

Abbreviations: E, evergreen; FR, riparian forest; SD, semideciduous.

^a^ Authors cited by Kouamé et al. (2004).

According to Kindt and Coe (2005), we recorded the number of species for several sites in most situations. We did not cover the whole area of interest. Accordingly, we do not know the species composition of the unsampled area. However, as species richness depends on sample size, we cannot expect to have recorded all species present in the study area. Richness estimators were therefore used to estimate the total number of species in the riparian forests surveyed. The approach used is as follows: based on the presence/absence matrix (198 surveys × 828 species), species accumulation curves were drawn to determine whether the surveyed sites were sufficiently sampled. Next, first and second‐order jackknife richness estimators (Jack1, Jack2) were used to estimate the total number of species in the surveyed riparian forest. Different methods of mathematical richness extrapolation exist, but the jackknife technique was chosen to predict total richness because it has been shown to outperform other estimators). Jack1 and Jack2 estimate the minimum and maximum number of species, respectively.

##### Species diversity and habitat heterogeneity analysis

To assess the habitat diversity and heterogeneity level, gamma, alpha, beta diversity, and Shannon's diversity index were calculated. Gamma, alpha, and beta diversity were used as basic diversity indices (Whittaker, 1972). Gamma diversity, the diversity at the landscape scale, is calculated as the total number of species or total richness across plots. Alpha diversity is calculated as the average species richness per plot. Beta diversity is a measure of data heterogeneity or habitat diversity (Mccune et al., 2002). The classical metrics were used to determine beta diversity: βw = (Sc/S) − 1, where βw is beta diversity, Sc is the number of species in the composite sample (the number of species in the dataset), and S is the average species richness in the sample units. If βw = 0, then all sample units have all species. Dataset heterogeneity increases with increased βw. As a rule, in this context, βw < 1 is rather low and βw > 5 can be considered high. The maximum value of βw is Sc − 1, which is obtained when the sampling units do not share any species. The Shannon's diversity index, derived from information theory, considers the number of species present in survey *i* (*n*_*i*_) and the relative cover (R_*ij*_) of the different species *j* in survey *i* (Shannon & Weaver, 1949). This index quantifies the heterogeneity of the biodiversity of a study area and thus indicates changes over time. The index decreases (≈0) when the number of species is low and only few species are dominant.

$$H^{\prime }=-\sum_{i=1}^{S}p_{i}.\log_{2}\left(p_{i}\right)$$

*H*′: Shannon biodiversity index; *i*: a species in the study area; *S*: species richness; *p*_*i*_: proportion of a species *i* in relation to the total number of species (*S*) in the study environment, which is calculated as follows: where is the number of individuals for species *i* and *N* is the total number of individuals; log2: logarithm to base 2.

##### Species rarity analysis

The rank‐frequency curve has been used to analyze rarity. This curve is generally represented by a graph, where the ranks of the species are plotted on the *x*‐axis in order of decreasing abundance, and the absolute or relative frequencies in the collection examined are plotted on the *y*‐axis in a logarithmic or semi‐logarithmic scale, which gives a more complete picture of the population than simple diversity index. Three types of rank‐frequency curves exist. However, we have chosen the curve cited by Frontier (1976) because it provides information on the diversity of the populations in the environments surveyed. It is based on a distribution represented by a straight line in semi‐logarithmic coordinates (rank in arithmetic scale; frequencies in logarithms). In the model based on semi‐logarithmic scales, we mostly observe not a single alignment, but a succession of rectilinear segments, suggesting the coexistence of several “stands,” useful for interpreting forest refuges. Generally speaking, the rarest species constitute a tail of distribution with rapidly decreasing numbers. However, 1% frequency threshold corresponding to 1 or 2 species‐occurrence was chosen in this study; all species with ≤1% frequency were considered rare.

##### Species turnover analysis

To assess whether plant assemblages in the Togolese highland riparian forest show a spatial turnover pattern and to understand the cause of the processes underlying species richness, Baselga's (2010) beta diversity partitioning method was used. According to Baselga, beta diversity can reflect two different phenomena: nesting and spatial turnover. Nesting of species assemblages occurs when biotas at sites with few species are subsets of biotas at rich sites, reflecting a nonrandom species loss process as a result of any factor favoring orderly assemblage disaggregation. In contrast to nesting, spatial turnover involves the replacement of some species due to environmental screening or spatial and historical constraints. The method, based on dissimilarities between multiple sites, combines three classical multiplicative metric formulations: total beta diversity (bSOR), spatial turnover (bSIM), and nesting (bNES). The beta.SOR, beta.SIM, and beta.NES formulas calculate Sorensen‐based, Simpson‐based, and nesting‐based multi‐site dissimilarities, respectively. The Beta.SOR and Beta.SIM indices measure beta diversity on a standard scale of zero to one. The presence–absence matrix (198 records × 828 species) was used as an input table for the analysis. The analysis was performed with R software (R Development Core Team, 2011), using the “beta‐multi.R” functions (Baselga, 2010).

#### 2.3.2 Endemism analysis

The sub‐Saharan tropical rainforests were defined by White (1979, 1983) as a phytogeographic domain comprising about 8000 plant species, of which 80% are endemic. These forests have been subdivided into sub‐centers of endemism (Hardy et al., 2013; Linder et al., 2012; Poorter et al., 2004). In this study, the analysis of endemism was based on the chorological subdivisions reported by White (1979, 1983) and Aké Assi (1984): (1) *Cosmopolitan* (Cosm), which includes species widely distributed on all or nearly all continents without particular centers of distribution; (2) *Pantropical* (Pan), which includes species occurring in the three sectors of the tropical zone (America, Africa + Madagascar, and Asia + Australia); (3) *Paleotropical* (Pal), which includes species distributed in the Old World (Africa + Madagascar and Asia + Australia); (4) *Afro‐American* (AA), which includes species in Africa and tropical America; (5) *Afro‐Malagasy* (AM), which includes species distributed in Africa, Madagascar and neighboring islands; (6) *Guinean‐Congolese Regional Endemism Center* (GC), which includes species occurring in the sub‐Saharan humid forest block; (7) *Upper Guinea sub‐center of endemism* (GCW), which includes species distributed in the moist forest block west of the DG; (8) *Lower Guinea and Congolese sub‐center of endemism* (GCE), which includes species distributed in the moist forest block east of the DG; (9) Dahomey Gap (DG), which includes species occurring in the DG; (10) *Sudan‐Zambezi regional center of endemism* (SZ), which includes species distributed in the dry forest and savannah domain; (11) *Guineo‐Congolian‐Zambezi regional transition zone and Guineo‐Congolian‐Sudanese* (GC‐SZ), which includes species distributed in the forest‐savannah transition zone; (12) *Taxa of introduced origin* (I) that have been introduced and cultivated or naturalized in tropical Africa. These species originate from other tropical areas (America, Asia, Australia, and New Zealand) [such as *Artocarpus altilis* (Parkinson) Fosberg, *Cassia siamea* Lam, *Cedrela odorata* L., *Gmelina arborea* Roxb., *Hevea brasiliensis* (Kunth) Mull.arg., *Mangifera indica* L., *Tectona grandis* L. f., and *Theobroma cacao* L.]. Phytogeographic characteristics have been assigned to species in the general list of riparian forests using specialized studies (Adjossou, 2009; Adou et al., 2005; Aké Assi, 1984; Akpagana, 1989; Aubréville, 1959; Berhaut, 1967–1988; Brunel et al., 1984; Guelly, 1994; Hawthorne & Jongkind, 2006; Hutchinson et al., 1954–1972; Kokou, 1998; Lebrun & Stork, 1991–1992; Natta, 2003; Poorter et al., 2004), and the African Plant Distribution Database (https://www.ville‐ge.ch/musinfo/bd/cjb/africa/index.php?langue=an).

### 2.4 Representativeness of rainforest species and forest type analysis

The relative proportion of rainforest species in the riparian forests of the Togolese Mountains was assessed according to the procedure reported by Meave and Kellman (1994), who considered the species of not only the rainforest block but also the xeric environments surrounding the rainforest zone. In this study, these xeric species include the GC‐SZ regional or forest‐savanna transition species. To ensure that the species inventoried in the forests of the Togolese mountain riparian areas were from moist forests, they were compared with the list of moist forest species of West Africa (Holmgren et al., 2004). For ambiguous species, they were compared with the African plant database.

Forest types in vegetation can be identified based on the occurrence of typical tree species (Langenberger et al., 2006). In sub‐Saharan Africa, forest types and their characteristic species are known from previous studies (Akpagana, 1989; Hall & Swaine, 1976; White, 1983). To assess the forest types present in the riparian forests studied, the forest type classification of White (1983) and Hall and Swaine (1976), reported by Jongkind et al. (2006), was used. It should be noted that not all species in the general floristic list were systematically assessed according to the forest types to which they belong, only a few typical tree species were selected to show the presence of forest types in the riparian forests studied.

### 2.5 Similarities between riparian vegetation and large sub‐Saharan forest refuge analysis

To assess the similarity between the riparian vegetation studied and the large sub‐Saharan forest refuges, the shared species were analyzed. This method was used because, in ecology, the number of shared species is a standard measure of similarity between two communities, and shared areas in species distribution are assumed to indicate a unique shared biological history (Lenormand et al., 2019). First, we checked whether riparian forests, and not the whole study area, were used as forest refugia. Therefore, the floristic list of riparian forests was first compared with the floristic list of previous inventories for vegetation lists of non‐riparian forests only, i.e., “Terre firme” forests, and vegetation matrix lists of whole forests. The non‐riparian forest vegetation lists were derived from the “Terre firme” forest inventory (Adjossou, 2009; Akpagana, 1992), while the whole forest matrix vegetation lists were derived from the compilation of available floristic lists and herbarium data on the whole forest vegetation of the study area (Adjossou, 2004, 2009; Akpagana, 1989, 1992; Brunel, 1977, 1978; Brunel et al., 1984). Second, the floristic list obtained from the riparian forests was compared with floristic lists representative of sites considered as major forest refuges in sub‐Saharan tropical Africa, including the Taï Forest Reserve located between southwest Côte d'Ivoire and Liberia near the large forest refuges of Palmas Cape and the Nimba Mountains; the DG vegetation sensu stricto located between Togo and Benin, part of the large forest refuges between Ghana and Benin; and the Haute Sangha forest (Republic of Congo) located in the large forest refuge areas of southern Cameroon—northern Gabon. For the forest refuges of southwest Côte d'Ivoire, the complete list of vascular flora established by Adou et al. (2005) was used. For the DG, the complete lists of vascular flora in the forest islands of the coastal plain of Togo established by Kokou (1998, 1999) and the riparian forests of Benin established by Natta (2003) were used. For the North Congolese forest refugia, the floristic list of woody species (DBH = 10 cm) established by Kimpouni et al. (2013) was used. To address the shortcomings of the floristic evidence in answering the biogeographical questions raised by the study, we compared our floristic evidences with those of palynological, phylogeographical, and palaeoecological studies.

## 3 RESULTS

### 3.1 Floristic diversity and turnover

**FIGURE 2:**
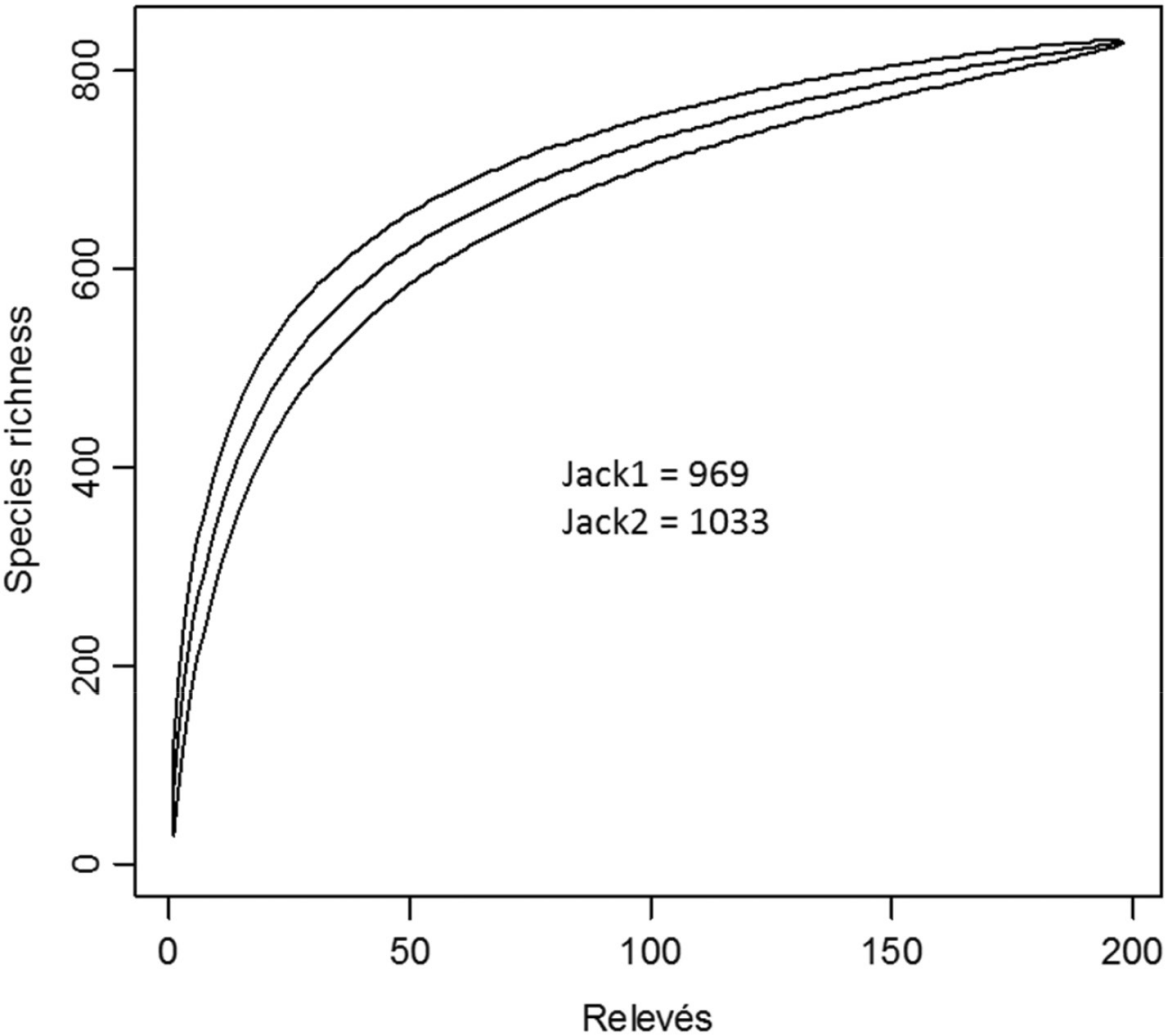
Area‐species curve of the Togolese mountain riparian vegetation. The area‐species curve did not stabilize with the sampling effort indicating that not all species were identified during the surveys. Estimates (first and second order jackknife richness estimators: Jack1, Jack2) show that at least 101 and at most 165 species remain to be identified, thus increasing the species richness of the surveyed riparian forests to at least 969 and at most 1033 species.

The results show an exceptional species richness in the riparian forests of the Togolese Highlands, amounting to 868 vascular species (Appendix S1) divided into 506 genera and 115 families. Rubiaceae (88 species) and Fabaceae (87) are the most abundant Angiosperm families in the riparian flora of the Togolese Mountains, followed by Apocynaceae (44), Malvaceae (42), Moraceae (31), and Euphorbiaceae (27). The most abundant genera are *Ficus* with 25 species, *Dioscorea* (13), *Canthium* (9), and *Drypetes* (8) (Table 2). This richness represents almost a third of the Upper Guinean forest flora richness. It is also comparable to that of the richest forest sites in sub‐Saharan Africa (Table 1). The area‐species curve did not saturate with the sampling effort (Figure 2), indicating that not all species were identified during the surveys. Estimates show that at least 101 and at most 165 species remain to be identified, thus increasing the species richness of the surveyed riparian forests to at least 969 and at most 1033 species.

**TABLE 2:** The 25 most common plant families and genera recorded in Togo mountain riparian forest community according to the classification systems of Cronquist and APGIII.

Cronquist		APGIII		Cronquist APGIII	
Families	Nb. species	Families	Nb. species	Genera	Nb. Species
Rubiaceae	88	Rubiaceae	88	*Ficus*	25
Euphorbiaceae	52	Fabaceae	87	*Dioscorea*	13
Papilionaceae	47	Apocynaceae	44	*Canthium*	9
Moraceae	33	Malvaceae	42	*Drypetes*	8
Apocynaceae	29	Moraceae	31	*Garcinia*	7
Gramineae	24	Euphorbiaceae	27	*Grewia*	7
Caesalpiniaceae	22	Gramineae	24	*Psychotria*	7
Acanthaceae	21	Acanthaceae	21	*Strychnos*	7
Asteraceae	20	Asteraceae	20	*Albizia*	6
Sterculiaceae	19	Annonaceae	17	*Cassia*	6
Mimosaceae	18	Cucurbitaceae	17	*Combretum*	6
Annonaceae	17	Phyllantaceae	17	*Celtis*	5
Cucurbitaceae	17	Araceae	16	*Cola*	5
Araceae	16	Sapindaceae	16	*Commelina*	5
Sapindaceae	16	Meliaceae	14	*Dalbergia*	5
Asclepiadaceae	15	Dioscoreaceae	13	*Desmodium*	5
Meliaceae	14	Commelinaceae	12	*Diospyros*	5
Verbenaceae	14	Orchidaceae	12	*Dracaena*	5
Dioscoreaceae	13	Asparagaceae	11	*Hippocratea*	5
Clusiaceae	12	Lamiaceae	11	*Macaranga*	5
Commelinaceae	12	Celastraceae	10	*Momordica*	5
Orchidaceae	12	Clusiaceae	10	*Salacia*	5
Tiliaceae	12	Menispermaceae	10	*Tricalysia*	5
Loganiaceae	10	Combretaceae	9	*Tylophora*	5
Menispermaceae	10	Marantaceae	9	*Vitex*	5

**FIGURE 3:**
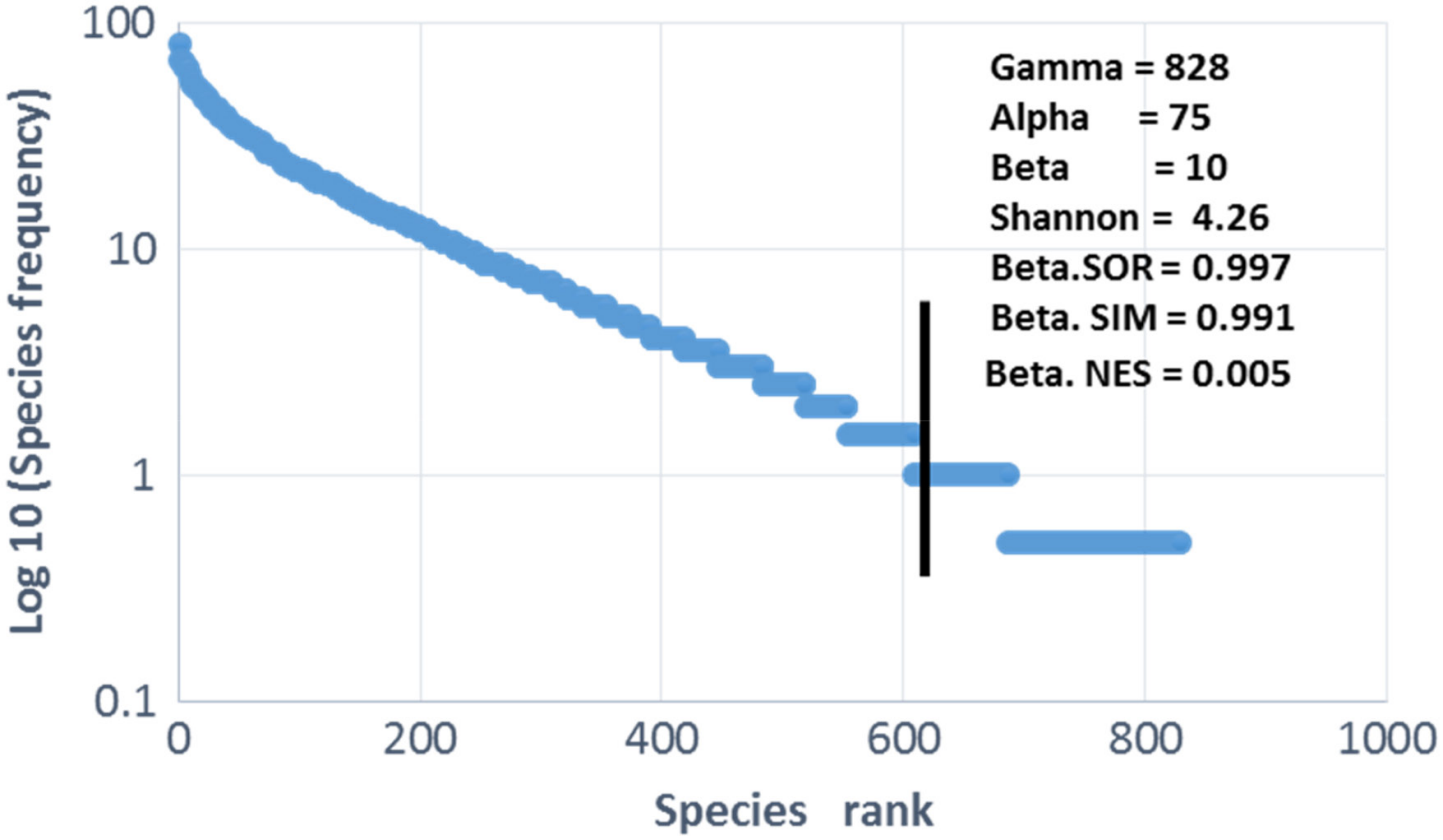
Rank‐frequency curve of the Togolese mountain riparian vegetation. The curve is represented by a graph, where the ranks of the species are plotted on the *x*‐axis in order of decreasing abundance and the relative frequencies are plotted on the *y*‐axis in semi‐logarithmic scale, which gives a more complete picture of floristic diversity than a simple diversity index. In the model based on semi‐logarithmic scales, we observe not a single alignment but a succession of rectilinear segments, suggesting the coexistence of several “stands” or “habitats”. The rarest species constitute a tail of distribution with rapidly decreasing numbers. However, 1% frequency threshold corresponding to 1 or 2 species‐occurrence was chosen in this study; all species with ≤1% frequency were considered rare. The vertical line intercepting the distribution marks the 1% relative frequency threshold, at which species are considered relatively frequent. The diversity indices are projected onto the graph. The Beta.SOR and Beta.SIM indices measure beta diversity on a standard scale of zero to one.

The value of gamma (828, *n* = 198), alpha (75.22 ± 22.92), beta (10), and Shannon (4.26) diversity indices are high (Figure 3), indicating a rich and diverse flora associated with high habitat heterogeneity. The rank‐frequency curve shows that a significant number of species (31%) could be considered rare as they were observed once or twice, i.e., a frequency of around 1% (Figure 3). *Pseudospondias microcarpa* (A. Rich.) Engl. was the most frequent species (81%), followed by *Canarium schweinfurthii* Engl. (68%), *Elaeis guineensis* Jacq. (67.7%), *Sterculia tragacantha* Lindl. (67.2%), *Costus afer* Ker‐Gawl. (64.6%), *Cleistopholis patens* (Benth.) Engl. & Diels (62.6%), *Funtumia africana* (Benth.) Stapf (62.6%), *Pycnanthus angolensis* (Welw.) Warb. (62.6%), *Alchornea cordifolia* (Schum. & Thonn.) Müll. Arg (58.6%), *Cola gigantea* var. glabrescens Brenan & Keay (56.6%), *Palisota hirsuta* (Thunb.) K. Schum. (55%), *Albizia zygia* (DC.) J.F. Macbr. (53.5%), *Phaulopsis barteri* (T. Anders.) Lindau (52.5%), *Trilepisium madagascariense* DC. (52%), *Aubrevillea kerstingii* (Harms) Pellegr. (51%), *Tabernaemontana pachysiphon* Stapf Var. cumminsii (51%).

Beta.SOR is mainly composed of bSIM (Beta.SOR = 0.9973, bSIM = 0.991, and bNES = 0.005; Figure 3), indicating that the beta diversity observed in the riparian forests of the Togolese Mountains reflects spatial turnover and not nesting. In other words, some species are replaced by others due to environmental selection and historical constraints.

### 3.2 Species composition and distribution

The forests studied contain bio‐indicator taxa of rainforest refugia such as slow‐dispersing taxa (*Amorphophallus*, *Anubias*, *Cercestis*, *Culcasia Rhaphidophora*, Rinorea spp., *Urera*, etc.), Caesalpinioideae and Rubiaceae and a paleoflora indicating the early origin of the vegetation studied.

**FIGURE 4:**
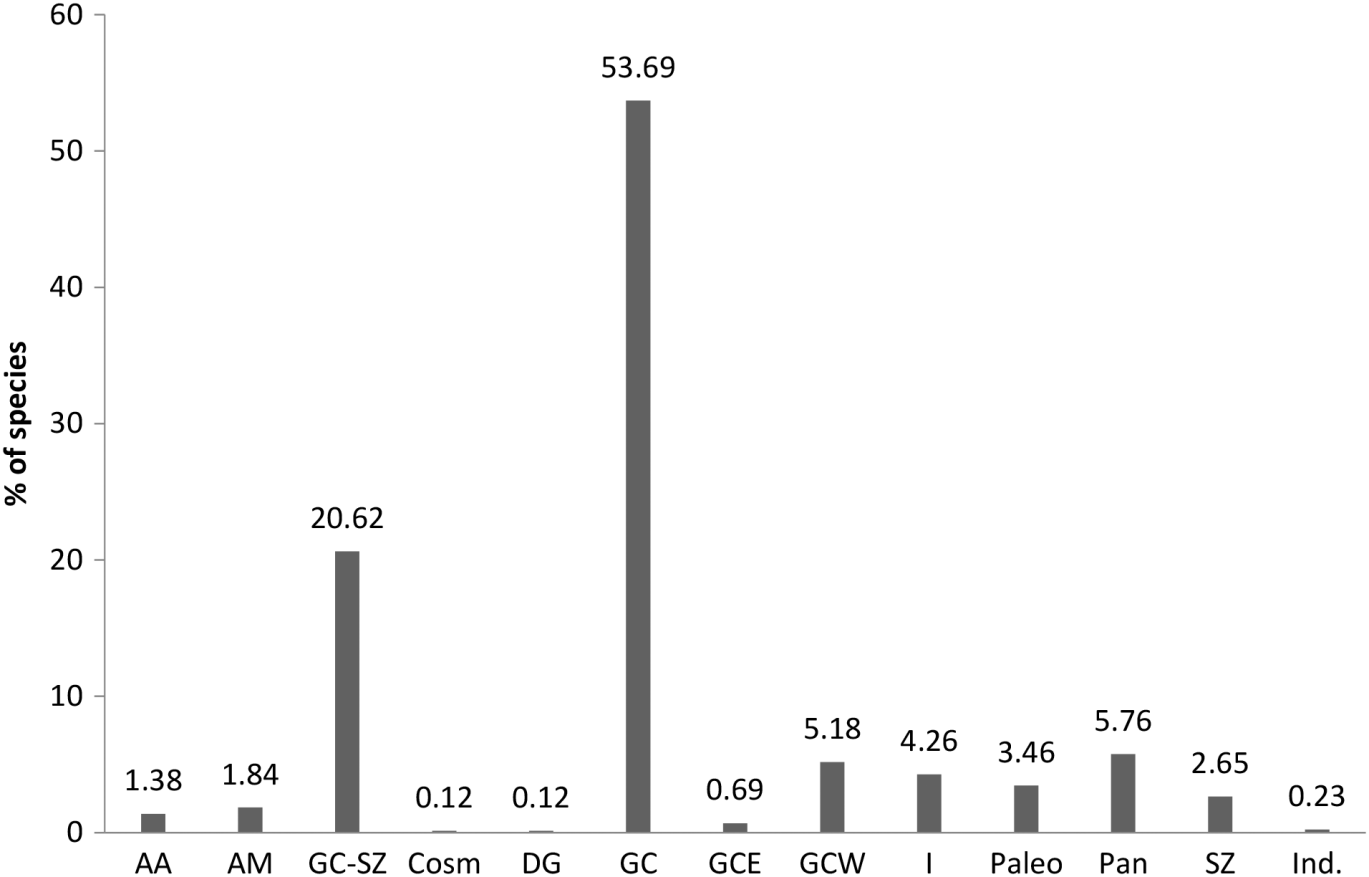
Phytogeographical origin of the sub‐humid Togolese mountain minor forest refuges flora: *Cosmopolitan* (Cosm), *Pantropical* (Pan), *Paleotropical* (Pal), *Afro‐American* (AA), *Afro‐Malagasy* (AM), Guinean‐Congolese regional center of endemism (GC), *Upper Guinean sub‐center of endemism including Western Guinean‐Congolese forest block* (GCW), *Lower Guinea and Congolese sub‐center of endemism* (GCE), Dahomey Gap (DG), Sudanese‐zambezian regional center of endemism (SZ), *Guineo‐Congolian‐Zambezi regional transition zone and Guineo‐Congolian‐Sudanese* (GC‐SZ), Introduced origine (I), Indeterminacy (Ind).

Species distribution according to phytogeographical origin showed that taxa from the GC represents 60% of the listed species, of which 54% was common to the western and eastern Guinean‐Congolese forest block, 5.30% was endemic to the western forest (GCW) including one endemic species from DG, and 0.70% was endemic to the Eastern forest (GCE). GC‐SZ species also occupied a relatively high proportion (21%; Figure 4).

Studied riparian comprise a high proportion of tropical rainforest species and typical elements of many tropical forest types. Among the 835 native species listed in our study, 93.89% were typical of tropical rainforests. Nearly all sub‐Saharan forests were represented in riparian vegetation by typical tree species such as moist evergreen forest [e.g., *Afzelia bella* Harms, *Afzelia bracteata* Vogel ex Benth. *Drypetes afzelii* Hutch. *Drypetes aylmeri* Hutch. & Dalziel, *Drypetes klainei* Pierre ex Pax, *Isolona cooperi* Hutch. & Dalziel ex Coop. & Record, *Parinari glabra* Oliv. *Xylopiastrum taiense* Aubré, and *Xylopia villosa* Chipp. Pentadesma butyracea]; and Upland evergreen forest [e.g., *Alchornea floribunda* Müll. Arg., *Leptaspis cochleata* Thwaites, *Lonchitis currori* (H.K.) Mett.ex Kühn, *Strombosia glaucescens* J.Leonard var. lucida, and *Tabernaemontana pachysiphon* Stapf] (Table 3). The forests studied contain most of the characteristic species of the Guinean‐Congolian Mixed Moist Forest, common to sub‐Saharan forest refuges sites (Table 4).

**TABLE 3:** Eleven sub‐Saharan African forest types and their typical species found in the riparian forests of the Togo Mountains.

No.	Forest type^a^	Characteristic species found in the gallery forests of Togo mountains
1	Ultra‐wet evergreen forest	NA
2	Wet evergreen forest	*Carapa procera* D C; *Cercestis afzelii* Schott; *Culcasia angolensis* Welw.; *Dacryodes klaineana* (Pierre) H.J. Lam.; *Dracaena ovata* Ker‐Gawl.; *Drypetes aylmeri* Hutct. & Dalziel; *Leptaspis cochleata* Thwaites; *Strombosia glauscescens* J.Leonard var. lucida.; *Uapaca guineensis* Müll. Arg.
3	Moist evergreen forest	*Afzelia bella* Harms; *Afzelia bracteata* Vogel ex Benth.; *Culcasia angolensis* Welw.; *Drypetes afzeli* Hutch. *Drypetes aylmeri* Hutch. & Dalziel; *Drypetes klainei* Pierre ex Pax, *Isolona cooperi* Hutch. & Dalziel ex Coop. & Record, *Leptaspis cochleata* Thwaites, *Parinari glabra* Oliv.; *Strombosia glauscescen*s J.Leonard var. lucida; *Xylopiastrum taiense* Aubré; *Xylopia villosa* Chipp.; *Pentadesma butyraccea* Sabine
4	Upland evergreen forest	*Alchornea floribunda* Müll. Arg.; *Culcasia angolensis* Welw.; *Leptaspis cochleata* Thwaites; *Lonchitis currori* (H.K.) Mett.ex Kühn; *Strombosia glauscescens* J.Leonard var. lucida; *Tabernaemontana pachysiphon* Stapf
5	Upland forest. Montane Forest	*Cola verticillata* (Thonn.) Stapf ex A. Chev.; *Eugenia calophylloides* DC.; *Parinari excelsa* Sabine, *Vangueriopsis discolor* (Benth.) Verdc
6	Moist Semideciduous forest	*Aningeria altissima* (A.Chev.) Aubrév. ex Pellegr.; *Aubrevillea kerstingii* (Harms) Pellegr.; *Celtis adolfi‐friderici* Engl.; *Celtis mildbraedii* Engl.
7	Dry semideciduous forest	*Antiaris africana* Engl.; *Celtis zenkeri* Engl.; *Mansonia altissima* (A.Chev.) A.Chev.var.altissima; *Milicia excelsa* (Welw)
8	Extreme dry forests	*Diospyros abyssinica* (Hiern) F. White; *Drypetes parviflora* Müll. Arg.Pax. & K. Hoffm.; *Lecaniodiscus cupanioides* Planch. & Benth.; *Millettia thonningii* (Schum. & Thonn). Bak.
9	Transition forest	*Anogeissus leiocarpus* (DC.) Guill. & Perr.; *Khaya grandifoliola* DC
10	Gallery (in savanna areas) or Riverine forest	*Ancytrophylum secundiflorum* (P.Beauv.) Wendl.; *Breonadia salicina* (Vahl) Hepper & Wood; *Cathormion altissimum* (Hook.f.) Hutch. & Dandy; *Cynometra megalophylla* Harms; *Hexalobus crispiflorus* A. Rich.; *Ixora brachypoda* DC.; *Maratia fraxinea* J.Smith.; *Pterocarpus santalinoides* L. Herit. ex DC.; *Spondiathus preussii* Engl.
11	Swamp forest	*Mitragyna stipulosa* O. Kuntze; *Raphia sudanica* A.Chev., *Spondiathus preussii* Engl., *Symphonia globulifera* L. f.; *Uapaca heudelotii* Baill., *Uapaca guineensis* Müll. Arg.

*Note*: Out of the 11 known forest types in the GC forest block, 10 are represented in the studied forests by their characteristic species. Only the ultra‐wet evergreen forest is not represented in the riparian forests of the Togolese Mountains. The ultra‐humid evergreen forest type may not be represented in the riparian forests of the Togolese Mountains due to the influence of the DG drought.

^a^ Forest type adapted from Hawthorne and Jongkind (2006).

**TABLE 4:** The characteristic tree species of the Guinean‐Congolian Mixed Moist Forest, common to sub‐Saharan forest refuges sites (White, 1983), also present in the studied forests

Species	Families
*Amphimas pterocarpoïdes* Harms	Fabaceae
*Blighia welwitschii* (Hiern) Radlk	Sapindaceae
Canarium schweinfurthii Engel.	Burseraceae
Celtis adolfi‐friderici Engel.	Canabaceae
*Celtis mildbraedii* Engl.	Canabaceae
*Celtis zenkeri* Engl.	Canabaceae
*Discoglypremna caloneura* (Pax) Prain.	Euphorbiaceae
*Distemonanthus benthamianus* Baill.	Fabaceae
*Entandrophragma cylindricum* (sprague) Sprague	Meliaceae
*Guarea cedrata* (A.Chev.)Pellegr.	Meliaceae
*Hannoa klaineana* Pierre et Engl. = Quassia klaineana	Simaroubaceae
*Klainedoxa gabonensi*s Pierre	Irvingiaceae
*Maesopsis eminii* Engl.	Rhamnaceae
*Mammea africana* Sabine	Clusiaceae
*Nauclea diderrichii* (de Wild. & Th. Dur.) Merril.	Rubiaceae
*Parinari excelsa* Sabine	Chrysobalanaceae
*Parinari glabra* Oliv. = Maranthes glabra	Chrysobalanaceae
*Parkia bicolor* A.Cheval.	Fabaceae
*Piptadeniastrum africanum* (Hook. f.) Brenan	Fabaceae
*Pseudospondias microcarpa* (A. Rich.) Engl.	Anacardiaceae
*Sterculia oblonga* Mast.	Malvaceae
*Sterculia rhinopetala* K. Schum.	Malvaceae
*Strombosia glauscescens* J.Leonard var. lucida = S. pustulata	Strombosiaceae

The results show a significant contribution of the studied forests to the current flora of large sub‐Saharan forest refuges. Indeed, The riparian vegetation studied shares 41% with the flora recorded in southwest Côte d'Ivoire, 76% with that of the humid forests of the study area, 100% with that of the entire matrix of the study area, 50% with that of the forest patches of the coastal plain of Togo, 41% with that of the riparian forests of Benin and 32.64% (dbh = 10 cm) with that of the humid forests of northern Congo (Appendix S1). The studied riparian vegetation shares 71% of its 868 species with that of the “terre firme” forests of the whole study area. This means that 29%, or 255, species are not common to both forests. These species are, mostly, those whose distribution is confined to the riparian corridor, probably due to historical events (Table 5).

**TABLE 5:** Some species of Togolese montage forests whose distribution is restricted to the riparian corridor

Species	Families
*Berlinia grandiflora (Valh) Hutch*. *& Dalz*.	Fabaceae
*Carapa procera DC*.	Meliaceae
*Cathormion altissimum (Hook*.*f*.*)Hutch*. *& Dandy*	Fabaceae
*Crotonogyne chevalieri (Beille) Keay*	Euphorbiaceae
*Dracaena ovata Ker‐Gawl*.	Agavaceae
*Drypetes afzeli Hutch*.	Euphorbiaceae
*Drypetes aylmeri Hutct*. & *Dalz*.	Euphorbiaceae
*Drypetes gilgiana(Pax)(Pax)&K*.*Hoffm*.	Euphorbiaceae
*Drypetes klainei Pierre ex Pax*	Euphorbiaceae
*Garcinia polyantha Oliv*. *=G*. *smeathmanii*	Clusiaceae
*Hexalobus crispiflorus A*. *Rich*.	Annonaceae
*Isolona cooperi Hutch*. *& Dalziel ex Coop*. *& Record*	Annonaceae
*Ixora brachypoda DC*.	Rubiaceae
*Pentadesma butyraccea Sabine*	Clusiaceae
*Pentaclethra macrophylla Benth*.	Fabaceae
*Phoenix reclinata Jacq*.	Arecaceae
*Pterocarpus santalinoides Linn*. *Herit*.*ex DC*.	Fabaceae
*Spondiathus preussii Engl*.	Euphorbiaceae
*Uapaca guineensis Müll*. *Arg*.	Euphorbiaceae
*Uapaca heudelotii Baill*	Euphorbiaceae

## 4 DISCUSSION

### 4.1 Floristic evidence of Togolese highland minor forest refugia

#### 4.1.1 Evidence based on species diversity and rarity

Our results showed that the species richness in the studied riparian forests is significantly high. The number of species listed in this study (868 species) is higher than those listed by Akpagana (1992) in the *“terre firme”* forest of the same area (648 species), and Kokou et al. (1999) in the forest patches of the whole coastal plain of DG in southern Togo (649 species). The total number of taxa listed in this study is rather of the same order as places considered to be very rich in sub‐Saharan Africa (Table 1). Very few data sets cover tropical riparian vegetation comprehensively and are therefore suitable for comparison. The only study using the same plot size was conducted by Natta (2003) in riparian forests of the Benin, Sudanese‐Guinean zone, who analyzed 198 vascular plant plots (500 m^2^), which recorded 556 species. In this study, with the same number of identically sized plots, 828 species were recorded, thus much higher than the number found by Natta (2003). These results support the biodiversity analysis made by Wieringa and Poorter (2004) for Upper Guinea, which considered Togo plateau as an extension of the mountain high diversity belt.

The riparian forests studied showed a high incidence of rare species, and a high level of beta and gamma diversity associated with species turnover, which is the evidence of forest refugia according to ecologists (Cowling & Lombard, 2002). The observed high beta diversity is an indication of a heterogeneous and complex environment, which is also characteristic of forest refuges (Do Prado et al., 2014; Ibarra Polesel & Damborsky, 2017; Naiman et al., 1993). Furthermore, our results showed patterns of spatial turnover indicating that the observed richness could be explained by both environmental and historical factors (Baselga, 2010). These results are therefore similar to the observations of Wieringa and Poorter (2004), who showed that environmental and historical factors explain 56% of the variation in rare and endemic species richness in West Africa and in line with the assumptions on the forest refuges. It is possible that the values of this index are influenced by the high number of rare species (30%) observed. However, the high incidence of rare species observed is not specific to the forests studied and is similar to those reported in the rainforests of the Amazon basin of Venezuela (Uhl & Murphy, 1981), on the northern slopes of the Barva Volcano in Costa Rica (Lieberman et al., 1996), in the Western Ghats of India (Amarnath et al., 2003), in the Philippines (Langenberger et al., 2006), and in West Africa (Holmgren et al., 2004; Wieringa & Poorter, 2004). The study on species distribution in West Africa shows that most rare species (observed in one or two collections) were found near the coast, on the border between Côte d'Ivoire and Ghana, and on the border between Côte d'Ivoire and Liberia, while the others were confined to the mountains. These areas coincide with the three postulated Pleistocene forest refuges: Cape Three points, Cape Palmas, and Mount Nimba (Holmgren et al., 2004; Wieringa & Poorter, 2004). Some of these species such as *Acanthus guineensis* Heine & P. Taylor, *Dracaena ovata* Ker‐Gawl, and *Pierrodendron kerstingii* (Engl.) Little were found in the Togo riparian zone in this study. However, the information on rarity must be qualified since the dominance of a few frequent species and the abundance of infrequent species is a general pattern in ecology, for terrestrial and aquatic systems, plants, or animals. It has deserved increasing attention in the last decade with the “hyperdominance” work (see Ter Steege et al., 2013).

#### 4.1.2 Evidence based on the presence of bio‐indicator species of rainforests refuges

In southwest Cameroon refugia, bio‐indicators species of rainforest refugia such as slow‐dispersing taxa (Begonia, Rinorea spp., etc.), Caesalpinioideae and Rubiaceae have been used to identify Central African rainforest refugia (Tchouto et al., 2009). These evidences were also present in the forests studied. For example, the *Rinorea* species were collected in the present study. The Begonia species were not collected in the present study but were included in Akpagana (1992) record made in the Togolese mountains. Besides, our results show that the studied forests are characterized by the co‐dominance of Rubiaceae–Fabaceae (88 Rubiaceae species against 87 Fabaceae species). In sub‐Saharan Africa, the dominance of Rubiaceae is an indicator of very humid environments. In the very humid southwestern forests of Côte d'Ivoire, Rubiaceae clearly dominates over Fabaceae (Adou et al., 2005), with 101 Rubiaceae species and 78 Fabaceae species. However, in the relatively dry DG, Fabaceae largely dominates over Rubiaceae. Kokou (1998) identified 134 Fabaceae species against 58 Rubiaceae species in the forest patches of the coastal plain of Togo, and Natta (2003) identified 134 Fabaceae species against 79 Rubiaceae species in the riparian forests of Benin. These results support the hypothesis that water balance appears to have been the main factor controlling vegetation during the Holocene (Rull, 2005). The presence of small groups of fragile plants with low dispersal power in the studied forests supports the existence of refuges in Togo Highlands. According to the biogeographical hypotheses based on the diversification of the groups of small fragile plants with low dispersal power (Blan, 1993), these would have diversification and abundance formerly more marked. The current species would have then been relict and testified to long wet past periods during which these groups would have specialized. The current climate, equally humid and favorable to these fragile species, would have been preceded by dry climates, during which these groups would have undergone strong regressions and persisted only in refuges (mountains, waterfalls,). According to Blan (1993), the current presence of these plants at low altitudes in areas surrounded by mountains, as is the case in Cameroon, Congo, and Gabon, suggests that the current wet period has been long enough to allow these low dispersal species to recolonise the lowland forests.

#### 4.1.3 Evidence based on the representativeness of forest types and proportion of humid tropical species

Studied riparian comprise a typical element of many tropical forests types. Out of the 11 known forest types in the GC forest block, 10 are represented in the studied forests by their characteristic species. Only the ultra‐wet evergreen forest is not represented in the riparian forests of the Togolese Mountains. Thus, we can say that almost all sub‐Saharan tropical forest types were represented in the studied riparian forests by their typical tree species. Furthermore, our results show that 93.89% of the 835 native species listed in the studied riparian forest were typical of tropical rainforests. This percentage is slightly higher than that reported (85.3%) by Pither and Kellman (2002) in the fragments of riparian forests in savannas of the Mount Pine Ridge and agreement with the hypotheses on forest refugia studied in sub‐humid tropical landscapes worldwide (Meave et al., 1991; Meave & Kellman, 1994; Pither & Kellman, 2002).

In addition, the studied riparian vegetation shares 71% of its 868 species with that of the “terre firme” forests of the whole study area. This means that 29%, or 255, species are not common to both forests. This is because many species are restricted to the riparian corridor (Table 5). Simultaneously, some species identified in the “mainland” forests were not found in the riparian forests. We do not believe that these species are restricted to the “mainland” forests, as information on the ecology of some of them shows that they could also inhabit the riparian forests. It is therefore possible that these species exist, particularly as estimates show that there are several unidentified species in the riparian forests surveyed. It is clear from these analyses that it was not the mountains of Togo as a whole that served as refuges for tropical rainforests, but it was the riparian forests from which the post‐Pleistocene recolonization took place.

#### 4.1.4 Evidence based on endemism

Our results showed that 60% (517 species) of the species studied have a GC endemic distribution pattern indicating very high endemism levels in the studied forests. These results are also consistent with the Pleistocene refuge model, which predicts that areas with a high number of endemic species probably served as refuges for tropical forest taxa during the expansion of savannas during the Ice Age in Europe, corresponding to the period of high heat in the tropics (Fiaschi & Pirani, 2009).

#### 4.1.5 Evidence based on affinity with vegetation and flora of forest refuges

The vegetation of the studied riparian forests have an affinity with those of the forest refuges in sub‐Saharan Africa. White (1983) described a forest type in sub‐Saharan Africa qualified as Guinean‐Congolian mixed moist forest. It seems that this forest type is the characteristic forest of refuge sites. According to him, most Guinean‐Congolian rainforests belong to this type. It occurs on well‐drained soils throughout the Guinean‐Congolian region except in the wettest and driest extremities. It is relatively undeveloped in West Africa due to the rapid increase in severe drought (It is easily understood that the ultra‐humid evergreen forest type may not be represented in the riparian forests of the Togolese Mountains due to the influence of the DG drought). Instead, it covers an enormous area in the heart of the eastern rainforest block, including northeastern Gabon, south‐eastern Cameroon, southwestern Central African Republic, northern Republic of Congo, and most of the Zaire basin and its periphery. The dominant vegetation is a semi‐evergreen rainforest of mixed composition. The mixed semi‐evergreen humid forest is relatively rich in terms of flora and most species of this forest type are widely distributed. White has listed the characteristic species of the mixed moist semi‐evergreen rain forest, all of which are found in the riparian forests studied (Table 4).

#### 4.1.6 Consistent floristic evidence with those of the palynological, phylogeographical, paleobotanical, and geological

Our results are in favor of a long‐term origin of the Togo Mountains as demonstrated by the palaeobotanical, palynological, and geological studies. Indeed, it is accepted that the differentiation of biogeographical units goes back a long way in the Earth's history. Genera are necessarily older than species and families are older than genera. Consequently, the higher the taxonomic level on which the subdivision into biogeographical units is based, the further back in Earth's history the events that gave rise to them. Empires and regions would be characterized by families and even orders, and would thus be a copy of continental drift. The New Zealand flora is often cited as being of Gondwanan origin. Comparison of our results with those obtained in New Zealand rainforests (Jaffre et al., 1994) shows a perfect similarity between these two floras with regard to the representativeness of the families and genera (Table 6). Fossil data reported by Craw et al. (1999), which has been cited by Craw et al. (1999), show that the Marattiaceae family dates back to the Carboniferous and the Cyatheaceae to the Cretaceous. These taxa are also present in the forests studied.

**TABLE 6:** Gondwanan taxa in Togolese riparian

Families	Genera
Adiantaceae, Agavaceae, Araceae, Arecaceae, Aspleniaeae, Cyatheaceae, Cyperaceae, Commelinacae, Dioscoreaceae, Euphorbiaceae, Graminae, Liliaceae, Orchidaceae, Pandanaceae, Polypodiaceae, Taccaceae, Rubiaceae, Schizeaceae, Smilacaceae	*Abru*s, *Albyzia*, *Alstonia*, *Caesalpinia*, *Canarium*, *Cyathea*, *Desmodium*, *Diospyros*, *Drypetes*, *Eugenia*, *Ficus*, *Garcinia*, *Gouania*, *Gymnema*, *Homalium*, *Hugonia*, *Jasminum*, *Lygodium*, *Mammea*, *Manilkara*, *Maytenus*, *Mesoneuron*, *Mimusops*, *Morinda*, *Mucuna*, *Pendanus*, *Phyllanthus*, *Polyscias*, *Premna*, *Pseuderanthemum*, *Psychotria*, *Rauvolfia*, *Secamone*, *Stephania*, *Syzygium*, *Terminalia*, *Tinospora*, *Tylophora*, *Vantilgo*, *Vitex*, and *Xylopia*

*Note*: The New Zealand flora is often cited as being of Gondwanan origin. Comparison of our results with those obtained in New Zealand rainforests (Jaffre et al., 1994) shows a perfect similarity between these two floras with regard to the representativeness of the families and genera.

The Paleobotanical research has provided information on the nature of the various tropical vegetation patterns that developed through central and northern Africa from the Cretaceous to the end of the Tertiary and has brought to light several stages in the development of the rainforest (Elenga et al., 1991, 1994; Maley, 1991, 1996). According to these sources, it is in the upper Eocene, about 45 million years ago, that the floristic composition of the south Cameroon vegetation really begins to resemble its present state. It is at this time that many taxa still living today appear. For instance; *Bombax buonopozense*, *Pentaclethra*, *Symphonia globulfera*, etc. The presence of these elements in the current flora indicated that the humid tropical flora would have been differentiated long before the Pleistocene era in the DG and around. We rejected any idea of seed transport of these species, especially by water, as the Togo Mountains are relatively isolated from the rest of the rainforests and are at a higher altitude than the surrounding drier forests. These results are in line with geological studies that suggest that the mountains of Togo date from the Late Precambrian (Hall & Swaine, 1976). The mountains of Togo can therefore bear witness to very ancient historical events.

Indeed, the distribution of current forests and savannas in West and Central Africa is thought to be the legacy of the long‐term history of climate and human impacts. Palaeo‐environmental reconstructions suggest that West and Central African forests have experienced a succession of contraction and extension in response to dry and humid periods since the Last Glacial Maximum (LGM, ~21 cal ka BP; Bengo & Maley, 1991; Daniau et al., 2020; Elenga et al., 1991, 1992, 1994; Giresse et al., 1991; Maley, 1992). The forests studied would have experienced these events. Indeed, palynological studies have concluded on the existence of a mountain climate during the Late Pleistocene, implying a decrease in mean temperature of at least 2–3°C. This cooler climate, which occurred between 28,000 years BP and 9000 years BP, would have favored the spread of montane trees such as *Olea hochstetteri* and *Polyscias fulva* (Maley & Brenac, 1998). *Polyscias fulva*, currently found on Mount Cameroon and further north on the Cameroonian Ridge, are also present in the forests studied. *Polyscias fulva* lives in association with *Parinari excelsa* at high altitude in the Togo mountain. The occurrence of low dispersal elements in these forests gives also evidence of that humid phase. Sosef (1994, 2004) had noted the presence of seven species of Begonia occurring in the Monts Doudou Reserve, which are recognized as indicators of a Pleistocene forest refuge. This has been confirmed by phylogenetic data. Molecular dating of the African genus *Begonia* indicated that these taxa originated during the Pleistocene (Plana et al., 2004). Tree species of the genera *Carapa* (Meliaceae) and *Coffea* (Rubiaceae) originated during the Pleistocene (Brée et al., 2020; Koenen et al., 2015) were also found in the studied forests.

In addition, Palynological and phylogenetical studies had described the Holocene flora (Elenga et al., 1992, 1996; Maley, 2001; Ngomanda et al., 2005; Reynaud‐Farrera, 1996; Schwartz et al., 1990; Souwnmi, 1999; Vincens et al., 1994, 1998). Elements of these flora such as *Celtis*, *Holoptelea grandis*, etc. (dating from 5100–4200 year B.P.) on the one hand and Moraceae, *Alchornea*, *Elaeis guineensis*, etc. (1100–800 year B.P.) on the other hand, are represented in the studied forests indicating that these forests have experienced Holocene events, i.e., the recovery of vegetation during the early/mid‐Holocene followed by its retreat during the late dry phase.

### 4.2 Contribution of sub‐Saharan minor forest refugia to larger sub‐Saharan forest refugia

The forests studied would contribute about 60% of their rich flora to the large sub‐Saharan forest refuges above mentioned. The riparian vegetation studied shares 41% of its 868 species with the flora recorded in southwest Côte d'Ivoire (Adou et al., 2005), which is located in the sector of the three major Pleistocene forest refuges of West Africa (Cap Trois Points, Cap Palmas, and Mount Nimba). This percentage, 41%, is clearly underestimated since several species were identified in the riparian forests studied, including Dictyandra arborescens Welw. ex Hook. f., Parkia filicoidea Welw. ex Oliv., *Pterocarpus mildbraedii Harms*, *Sterculia rhinopetala* K. Schum., *Stephania dinklagei* (Engl.) Diels, and *Trichilia megalantha* Harms, would appear in the forests of southwest Côte d'Ivoire according to the African plant database but not Adou et al., 2005 records. Considering these typically Guinean‐Congolese species, the number of shared species would reach 60%. Almost all of these are not recorded by Kokou (1998, 1999) in the forest patches of the Togolese coastal plain and Natta (2003) in the riparian forests of Benin but are recorded by Kimpouni et al. (2013) in the Upper Sangha forest (Republic of Congo) located in the large forest refugia of southern Cameroon and northern Gabon. These species have also been described by White as characteristic species of the Guinean‐Congolian mixed semi‐evergreen rainforest in northeast Gabon, south‐east Cameroon, southwest Central African Republic, northern Congo Republic, and most of the Zaire basin and its periphery. These results show that the riparian forests of the Togolese Mountains have more affinity toward the vegetation of the large sub‐Saharan forest refugia than toward the vegetation of the DG sensu stricto, because they contain many species present in very humid forest. Therefore, recent work under the ECO SYN project (Poorter et al., 2004) has linked the sub‐humid mountains of Togo to the West African humid forest block separately from the relatively drier DG.

The above statements do not identify the importance of DG as a forest refuge. It is true that rainforest species (GC) decline markedly in the DG compared to that in the sub‐humid Togolese montane, but the riparian forests studied share 40%–50% species with the DG vegetation, most of which are hybrid species (GC‐SZ) that can live in both wet and relatively dry habitats. For example, the studied riparian vegetation shares 50% of its 868 species with that of the forest patches of the coastal plain of Togo and 40% with that of the riparian forests of Benin. The influence of the sea and the existence of some rivers in the DG allowed relict species to maintain their populations during the Pleistocene warm‐up. Some of these species, such as *Alstonia congensis* Engl., which is currently found in the Haho River basin, and *Balanites wilsoniana* Dawe & Sprague and *Schrebera arborea* A. Chev., identified in the Mono River basin in the DG (Kokou, 1998), testify the important role played by rivers during the Pleistocene warming period. These results therefore support the hypothesis that there is a rapid increase in severe droughts in West Africa, in this case around the DG. However, our results suggest that the DG, except the Togolese Mountains, would have provided little refuge for Guinean‐Congolian moist forest species during the arid Pleistocene period. By contrast, several species had found refuge in the wet valley bottoms of rivers in the Togolese Mountains. Three factors could justify the absence of most of the species of the Guinean‐Congolian rainforest from the forests of the coastal plain of Togo and the riparian forests of Benin. First is the severity of the aridity in the DG. During the Pleistocene period, the DG would be more affected by aridity. Second is the absence of mountains that could provide refuges. Third is the geology that would offer few refuge substrates.

### 4.3 Implications for sub‐Saharan Tropical African biogeography and biodiversity conservation

The origin of the DG is one of the central biogeographical questions in sub‐Saharan Africa, which has been addressed in recent phylogenetic studies. Till date, no study based on large floristic data collected in the DG has addressed this question. Our work, based on large floristic data collected in the DG and compared with those of other studies suggests that the riparian forests of the sub‐humid Togolese Mountains would have served as important minor forest refugia during recurrent climatic episodes.

Indeed, the Cross River biogeographic break, like other smaller breaks identified in other places is thought to be the result of past fragmentation of the forest block as a result of climatic changes. It appears, therefore, that the major breaks in the sub‐Saharan forest block must have been repeated many times with relative geographical constancy since the late Pliocene (Maley, 1991; White, 1979). Our results are in line with these conclusions and rejected the hypothesis of a single opening of the DG. The riparian forests of the Togolese mountains would have served as refuges during recurrent climatic crises during their history and would continue to play this role in future.

Our findings also seem to confirm the hypotheses that the riparian forests played the role of refuge for flora during the arid period of Pleistocene in sub‐humid tropical landscapes covered with savannas or deciduous forests. During this severe drought period, the riparian zones would have been the only forest places maintaining the conditions necessary for most species, and the current flora would have been reconstituted and differentiated from these zones when moisture conditions became favorable for a forest cover. This process could explain why the flora of the tropical riparian forests often contains a significant part of the flora of the neighboring forests (Meave et al., 1991; Meave & Kellman, 1994). These results therefore support the hypothesis that the forest fragmentation was minimal or took the form of a network of refugia (along rivers and/or in mountains) where most species survived.

The few phylogeographic studies conducted in the DG have suggested that the forest flora of the DG may be essentially relics from the early Holocene, when the Guinean‐Congolian forest reached its maximum geographical distribution (Demenou et al., 2016; Duminil et al., 2015; Koffi et al., 2011). Our floristic data do not seem to be compatible with these ideas. Prior to the last opening of DG, the differentiation of sub‐Saharan rainforests would have taken place under exceptionally wet conditions, allowing their maximum development. These conditions would probably coincide with the wet periods of the Late Pleistocene and Holocene (Salanville, 1992). We hypothesize that during this period of high humidity, the Rubiaceae family would be more differentiated than the Fabaceae family, since Rubiaceae would be more prevalent in the wetter forests (southwestern Côte d'Ivoire) than in the relatively drier ones (DG) where Fabaceae would be strongly represented. But the period of severe aridity following the last wet period would have impoverished DG in ancient forest species, which would be typical Guinean‐Congolese species. Moreover, it is widely accepted that evolutionary changes would have occurred in isolated populations in forest refugia. Models of Fabaceae and Gramineae studied in North Africa also argue that isolated populations would favor the emergence of genotypes adapted to environmental conditions (Amirouche & Michet, 2009). Speciation would have therefore contributed to the creation of hybrid individuals adapted to the relatively precarious climatic and soil conditions in forest refuges. Fabaceae and Gramineae would have been better adapted to the severe environmental conditions and served as pioneer and transitional species during the recolonization phase around forest refuges. They continue to play this role today in environments hostile to GC species. Our results seem to indicate that, in the DG, there are more adapted ecotypes in current transitional forests than relict species of ancient forests. This is the case of *Millettia thonningii* (Schum. & Thonn.) Bak. (Fabaceae), an endemic species of DG currently introduced in some sites in Africa. These observations raise important questions about the patterns of disjunction (dispersal, vicariance, and geographic separation; Dexter et al., 2017) that may be elucidated in further studies for understanding the processes of reconstitution and differentiation of the post‐Pleistocene DG flora.

Historically, the biogeographical theory suggested that large reserves are good for biodiversity conservation. In the real world of conservation planning, the opportunity to apply such guidelines is constrained by costs and patterns of land use history (Margules & Pressey, 2000). According to Margules & Pressey, if the area available for reservation is limited, a choice might be made between a few large reserves that favor the persistence of some species or many small reserves that together are more representative of the region's biodiversity but individually less effective for the persistence of some species. In sub‐Saharan tropical Africa, where maintaining a vast area of natural forest is difficult due to human pressure, efforts to preserve maximum species diversity should include a focus on the conservation of minor forest refuges, particularly in sub‐humid mountain riparian zone. In this scenario, the findings of Bohoussou et al. (2015) constitute beneficial for managing biodiversity in sub‐Saharan tropical Africa.

## 5 CONCLUSION

This study was undertaken with the aim of contributing to scientific debates on tropical biogeography and its importance for biodiversity conservation in the context of climate change. Our work, based on floristic evidence compared to those from palaeobotanical, palynological, phylogenetic, and geological studies, suggests that the mountains of Togo and their riparian forests would have very ancient origins. They would therefore have experienced paleoclimatic events as evidenced by the presence in these forests of paleoflora elements dating from the Cretaceous, Pleistocene, and Holocene. The study rejects the idea of seed transport of these species, especially by water, as the mountains of Togo are relatively isolated from the rest of the humid forest blocks and are at a higher altitude than the surrounding drier forests.

According to ecologists the major breaks in the sub‐Saharan forest block must have been repeated many times with relative geographical constancy since the late Pliocene. Our results are in line with these conclusions and rejected the hypothesis of a single opening of DG in Holocene. The studied riparian would have served as refuges during recurrent climatic episodes and would continue to play this role in future. Ours results therefore support the hypothesis that the forest fragmentation was minimal or took the form of a network of refugia (along rivers and/or in mountains) where most species survived. Isolated populations such as *Alstonia congensis* Engl., which is currently found in the Haho River basin, and *Balanites wilsoniana* Dawe & Sprague and *Schrebera arborea* A. Chev., identified in the Mono River basin in the DG are evidence of this.

The phylogeographic studies conducted in the DG have suggested that the forest flora of the DG may be essentially relics from the early Holocene, when the Guinean‐Congolian forest reached its maximum geographical distribution. Our floristic data do not seem to be compatible with these ideas.

In sub‐Saharan tropical Africa, where maintaining a vast area of natural forest is difficult due to human pressure, efforts to preserve maximum species diversity should include a focus on the conservation of minor forest refuges, particularly in sub‐humid mountain riparian zone.

## AUTHOR CONTRIBUTIONS

**Kossi Adjossou involved in** conceptualization (lead), data curation (lead), formal analysis (lead), funding acquisition (lead), investigation (lead), methodology (lead), project administration (lead), supervision (equal), validation (equal), writing—original draft (lead), writing—review and editing (lead), fieldwork and identified the specimens (equal). **Kouami Kokou involved in** funding acquisition (supporting), investigation (supporting), methodology (supporting), supervision (equal), validation (equal), writing—review and editing (supporting), fieldwork and identified the specimens (equal). **Marc Deconchat involved in** formal analysis (suppoting), methodology (supporting), supervision (equal), validation (equal), writing—review and editing (suporting).

## CONFLICT OF INTEREST

The authors declare no conflicts of interest.

## Supporting information


Appendix S1

## Data Availability

Data supporting the results of this study will be archived in the DRAYD data directory (URL: https://doi.org/10.5061/dryad.w6m905qsf).

## References

[ece39304-bib-0001] Adjossou, K. (2004). Diversité floristique des forêts riveraines de la zone écologique IV du togo (p. 75). (Master thesis). University of Lomé.

[ece39304-bib-0002] Adjossou, K. , & Kokou, K. (2009). Flore forestière de la zone montagneuse subhumide du togo (afrique de l'ouest). In X. Van Der Burgt , J. Van Der Maesen , & J.‐M. Onana (Eds.), Systématique et conservation des plantes Africaines (pp. 615–624). Royal Botanic Gardens.

[ece39304-bib-0003] Adjossou, K. (2009). Diversité, structure et dynamique de la végétation dans les fragments de forêts humides du togo: Les enjeux pour la conservation de la biodiversité (p. 190). (PHD Thesis). University of Lomé.

[ece39304-bib-0004] Adou, Y. , Blom, E. , Dengueadhe, K. T. S. , Van Rompaey, R. , N'guessan, E. K. , Wittebolle, G. , & Bongers, F. (2005). Diversité floristique et végétation dans le parc national de taï, côte d'ivoire [en ligne] . Researchgate.

[ece39304-bib-0502] Aide, T. M. , & Rivera, E. (1998). Geographic patterns of genetic diversity in *Poulsenia armata* (Moraceae): Implications for the theory of Pleistocene refugia and the importance of riparian forest. Journal of Biogeography, 25, 695–705. 10.1046/j.1365-2699.1998.2540695.x

[ece39304-bib-0005] Aké Assi, L. (1971). Présence d'un piper d'amérique du sud sur les pentes de la montagne klouto (togo). In H. Merxmüller (Ed.), Proceedings of the seventh plenary meeting of the Association pour l'Etude Taxonomique de la Flore de l'Afrique Tropicale (AETFAT) (p. 169). H. Merxmüller.

[ece39304-bib-0006] Aké Assi, L. (1984). Flore descriptive de Côte d'Ivoire: Etude descriptive et biogéographique avec quelques notes ethnobotaniques (p. 1206). (PHD Thesis). University of Abidjan.

[ece39304-bib-0007] Akpagana, K. (1989). Recherches sur les forêts denses humides du Togo (p. 181). (PHD Thesis). University of Bordeaux III.

[ece39304-bib-0008] Akpagana, K. (1992). Les forêts denses humides des monts togo et agou (république du togo). Adansonia, 14, 109–172.

[ece39304-bib-0009] Amarnath, G. , Murthy, M. S. R. , Britto, S. J. , Rajashekar, G. , & Dutt, C. B. S. (2003). Diagnostic analysis of conservation zones using remote sensing and gis techniques in wet evergreen forests of the western ghats – An ecological hotspot, Tamil Nadu, India. Biodiversity and Conservation, 12, 2331–2359.

[ece39304-bib-0010] Aubréville, A. (1959). La flore forestière de la côte d'ivoire (Vol. 3, 2nd ed.). Centre Technique Forestier Tropical.

[ece39304-bib-0511] Amirouche, R. , & Misset, M. T. (2009). Flore spontanée d'Algérie: Différenciation écogéographique des espèces et polyploïdie. Cahiers Agricultures, 18(6), 474–480.

[ece39304-bib-0011] Baselga, A. (2010). Partitioning the turnover and nestedness components of beta diversity. Global Ecology and Biogeography, 19, 134–143.

[ece39304-bib-0012] Bengo, M. D. , & Maley, J. (1991). Analyses des flux pollinitiques sur la marge sud du Golfe de Guinée depuis 135 000 ans. Comptes Rendus de l'Academie des Sciences de Paris Série II, 313, 843–849.

[ece39304-bib-0013] Berhaut, J. (1967–1988). Flore illustée du sénégal (Vol. 1–6, p. 9). Clairafrique et Maisonneuve Gouvernement du Sénégal.

[ece39304-bib-0014] Blan, P. (1993). Disjonction dans les flores de sous‐bois en Afrique. In J. L. Guillaumet , M. Belin , & H. Puig (Eds.), Phytogeographie tropicale réalités et perspectives (pp. 25–38). ORSTOM.

[ece39304-bib-0015] Boakye, E. A. , Gebrekirstos, A. , Hyppolite, D. N. , Barnes, V. R. , Porembski, S. , & Bräuning, A. (2019). Carbon isotopes of Riparian forests trees in the savannas of the Volta Sub‐Basin of Ghana reveal contrasting responses to climatic and environmental variations. Forests, 10, 251. 10.3390/f10030251

[ece39304-bib-0016] Bohoussou, K. H. , Cornette, R. , Akpatou, B. , Colyn, M. , Kerbis Peterhans, J. , Kennis, J. , Šumbera, R. , Verheyen, E. , N'goran, E. , Katuala, P. , & Nicolas, V. (2015). The phylogeography of the rodent genus malacomys suggests multiple afrotropical pleistocene lowland forest refugia. Journal of Biogeography, 42, 2049–2061.

[ece39304-bib-0017] Boyer, S. L. , Markle, T. M. , Baker, C. M. , Luxbacher, A. M. , & Kozak, K. H. (2016). Historical refugia have shaped biogeographical patterns of species richness and phylogenetic diversity in mite harvestmen (arachnida, opiliones, cyphophthalmi) endemic to the australian wet tropics. Journal of Biogeography, 43, 1400–1411.

[ece39304-bib-0018] Brée, B. , Helmstetter, A. J. , Bethune, K. , Ghogue, J.‐P. , Sonké, B. , & Couvreur, T. L. P. (2020). Diversification of African rainforest restricted clades: Piptostigmateae and Annickieae (Annonaceae). Diversity, 12, 227. 10.3390/d12060227

[ece39304-bib-0501] Broecker, W. S. , & Denton, G. H. (1989). The role of ocean‐atmosphere reorganizations in glacial cycles. Geochimica et Cosmochimica Acta, 53, 2465–2501.

[ece39304-bib-0019] Brunel, J. F. (1977). Contribution à l'inventaire floristique des angiospermes au togo. Annales Université du Bénin, 3, 161–182.

[ece39304-bib-0020] Brunel, J. F. (1978). Contribution à l'inventaire floristique des angiospermes au togo. Annales Université du Bénin, 4, 83–102.

[ece39304-bib-0021] Brunel, J. F. , Hiepko, P. , & Scholz, H. (1984). Flore analytique du togo. Phanérogames. Englera (Vol. 4, pp. 1–751). GTZ.

[ece39304-bib-0022] Carlson, S. E. , Linder, H. P. , & Donoghue, M. J. (2012). The historical biogeography of scabiosa (dipsacaceae): Implications for old world plant disjunctions. Journal of Biogeography, 39, 1086–1100.

[ece39304-bib-0023] Carnaval, A. C. , & Moritz, C. (2008). Historical climate modelling predicts patterns of current biodiversity in the brazilian atlantic forest. Journal of Biogeography, 35, 1187–1201.

[ece39304-bib-0024] Cheddadi, R. , Fady, B. , François, L. , Hajar, L. , Suc, J. P. , Huang, K. , Demarteau, M. , Vendramin, G. G. , & Ortu, E. (2009). Putative glacial refugia of cedrus atlantica deduced from quaternary pollen records and modern genetic diversity. Journal of Biogeography, 36, 1361–1371.

[ece39304-bib-0025] Cowling, R. M. , & Lombard, A. T. (2002). Heterogeneity, speciation/extinction history and climate: Explaining regional plant diversity patterns in the cape floristic region. Diversity and Distributions, 8, 163–179.

[ece39304-bib-0026] Craw, R. C. , Grehan, J. R. , & Heads, J. M. (1999). In A. Hallam , B. R. Rosen , & T. C. Whitmore (Eds.), Panbiogeography Tracking the History of life (p. 229). Oxford University Press.

[ece39304-bib-0027] Daniau, A.‐L. , Desprat, S. , Aleman, J. C. , Bremond, L. , Davis, B. , Wi, F. , Marlon, J. R. , Marquer, L. , Montade, V. , Morales‐Molino, C. , Naughton, F. , Rius, D. , & Urrego, H. (2020). Terrestrial plant microfossils in palaeoenvironmental studies, pollen, microcharcoal and phytolith. Towards a comprehensive understanding of vegetation, fire and climate changes over the past one million years. Revue de Micropaleontologie, 67, 100412. 10.1016/j.revmic.2019.02.001

[ece39304-bib-0028] Demenou, B. B. , Piñeiro, R. , & Hardy, O. J. (2016). Origin and history of the dahomey gap separating west and central African rain forests: Insights from the phylogeography of the legume tree distemonanthus benthamianus. Journal of Biogeography, 43, 1020–1031.

[ece39304-bib-0512] Dexter, K. G. , Lavin, M. , Torke, B. M. , Twyford, A. D. , Kursar, T. A. , Coley, P. D. , Drake, C. , Hollands, R. , & Toby Pennington, R. (2017). Dispersal assembly of rain forest tree communities across the Amazon basin. Proceedings of the National Academy of Sciences of the United States of America, 114(10), 2645–2650. 10.1073/pnas.161365511 28213498 PMC5347625

[ece39304-bib-0029] Do Prado, J. R. , Brennand, P. G. G. , Godoy, L. P. , Libardi, G. S. , De Abreu‐Júnior, E. F. , Roth, P. R. O. , Chiquito, E. A. , & Percequillo, A. R. (2014). Species richness and areas of endemism of oryzomyine rodents (cricetidae, sigmodontinae) in south america: An ndm/vndm approach. Journal of Biogeography, 42, 540–551.

[ece39304-bib-0030] Duminil, J. , Mona, S. , Mardulyn, P. , Doumenge, C. , Walmacq, F. , Doucet, J.‐L. , & Hardy, O. J. (2015). Late pleistocene molecular dating of past population fragmentation and demographic changes in African rain forest tree species supports the forest refuge hypothesis. Journal of Biogeography, 42, 1443–1454.

[ece39304-bib-0031] Dupont, L. M. , Schlütz, F. , Ewah, C. T. , Jennerjahn, T. C. , Paul, A. , & Behling, H. (2009). Two‐step vegetation response to enhanced precipitation in northeast brazil during heinrich event 1. Global Change Biology, 16, 1647–1660.

[ece39304-bib-1001] Eeley, H. A. C , Lawes, M. J. , & Piper, S. E. (1999). The influence of climate on the distribution of indigenous forest in KwaZulu‐Natal,South Africa. Journal of Biogeography, 26, 595–617.

[ece39304-bib-0032] Ehrich, D. , Alsos, I. G. , & Brochmann, C. (2008). Where did the northern peatland species survive the dry glacials: Cloudberry (rubus chamaemorus) as an example. Journal of Biogeography, 35, 801–814.

[ece39304-bib-0033] Elenga, H. , Vincens, A. , & Schwartz, D. (1991). Présenced’éléments forestiers montagnards sur les Plateaux Batéké (Congo) au Pléistocène supérieur: nouvelles données palynologiques. Palaeoecology of Africa, 22, 239–252.

[ece39304-bib-0034] Elenga, H. , Schwartz, D. , & Vincens, A. (1992). Changements climatiques et action anthropique sur le littoral congolais au cours de l'Holocène. Bulletin de la Société Géologique de France, 163(1), 83–90.

[ece39304-bib-0035] Elenga, H. , Schwartz, D. , & Vincens, A. (1994). Pollen evidence of late Quaternary vegetation and inferred climate changes in Congo. Palaeogeography, Palaeoclimatology, Palaeoecology, 109(2–4), 345–356.

[ece39304-bib-0036] Elenga, H. , Schwartz, D. , Vincens, A. , Bertaux, J. , de Namur, C. , Martin, L. , Wirrmann, D. , & Servant, M. (1996). Diagramme pollinique holocène du lac Kitina (Congo): Mise en évidence de changements paléobotaniques et paléoclimatiques dans le massif forestier du Mayombe. Comptes Rendus de l'Académie des Sciences, Série 2a: Sciences de la Terre et des Planètes, 323, 403–410.

[ece39304-bib-0037] Fayiah, M. , Kallon, B. F. , Dong, S. , James, M. S. , & Singh, S. (2020). Species diversity, growth, status, and biovolume of Taia River Riparian forest in Southern Sierra Leone: Implications for community‐based conservation. International Journal of Forestry Research, 2020, 2198573, 13 pages. 10.1155/2020/2198573

[ece39304-bib-0038] Fayolle, A. , Swaine, M. D. , Bastin, J.‐F. , Bourland, N. , Comiskey, J. A. , Dauby, G. , Doucet, J.‐L. , Gillet, J. F. , Gourlet‐Fleury, S. , Hardy, O. J. , Kirunda, B. , Kouamé, F. N. , & Plumptre, A. J. (2014). Patterns of tree species composition across tropical African forests. Journal of Biogeography, 41, 2320–2331.

[ece39304-bib-0039] Fiaschi, P. , & Pirani, J. R. (2009). Review of plant biogeographic studies in brazil. Journal of Systematics and Evolution, 47, 477–496.

[ece39304-bib-0040] Frontier, S. (1976). Utilisation des diagrammes rang‐fréquence dans l'analyse des écosystèmes. Journal de Recherche Océanographique, 1(3), 35–48.

[ece39304-bib-0041] Gautier‐Hion, A. , & Brugiere, D. (2005). Significance of riparian forests for the conservation of Central African primates. International Journal of Primatology, Springer Verlag, 26(3), 515–523. 10.1007/s10764-005-4363-1

[ece39304-bib-0042] Giresse, P. , Maley, J. , & Kelts, K. (1991). Sedimentation and palaeoenvironment in crater lake Barombi Mbo, Cameroon during the last 25,000 years. Sedimentary Geology, 71, 151–175.

[ece39304-bib-0044] Guelly, K. A. (1994). Les savanes de la zone forestière subhumide du togo (p. 163). (PHD Thesis). University Pierre et Marie Curie

[ece39304-bib-0045] Habel, J. C. , & Ulrich, W. (2021). Ecosystem functions in degraded riparian forests of southeastern Kenya. Ecology and Evolution, 11, 12665–12675. 10.1002/ece3.8011 34594529 PMC8462158

[ece39304-bib-0046] Hall, J. B. (1973). Vegetational zones on the southern slopes of mount cameroon. Vegetatio, 27, 49–69.

[ece39304-bib-0047] Hall, J. B. (1981). Ecological islands in south‐eastern nigeria. African Journal of Ecology, 19, 55–72.

[ece39304-bib-0048] Hall, J. B. , & Swaine, M. D. (1976). Classification and ecology of closed‐canopy forest in ghana. Journal of Ecology, 64, 913–951.

[ece39304-bib-0049] Hardy, O. J. , Born, C. , Budde, K. , Daïnou, K. , Dauby, G. , Duminil, J. , Ewédjéa, E.‐E. B. K. , Gomeze, C. , Heuertzac, M. , Koffiaf, G. K. , Lowegh, A. J. , Micheneauai, C. , Ndiade‐Bourobouj, D. , Piñeiroa, R. , & Poncet, V. (2013). Comparative phylogeography of African rain forest trees: A review of genetic signatures of vegetation history in the Guineo‐Congolian region. Comptes Rendus Geoscience, 345(7–8), 284–296.

[ece39304-bib-0050] Hawthorne, W. , & Jongkind, C. (2006). Woody plants of western African forests. A guide to the forest trees, shrubs and lianes from Senegal to Ghana. Royal Botanic Gardens.

[ece39304-bib-0051] Holmgren, M. , Poorter, L. , Siepel, A. , Bongers, F. , Buitelaar, M. , Chatelain, C. , Gautier, L. , Hawthorne, W. D. , Helmink, A. T. F. , Jongkind, C. C. H. , Os‐Breijer, H. J. , Wieringa, J. J. , & Van Zoest, A. R. (2004). Ecological profiles of rare and endemic species. In L. Poorter , F. Bongers , F. N. Kouame , & W. D. Hawthorne (Eds.), Biodiversity of west African forests: An ecological atlas of woody plant species (pp. 391–445). CABI Publishing.

[ece39304-bib-0043] Hood, W. G. , & Naiman, R. J. (2000). Vunerability of riparian zones to invasion by exotic vascular plants. Plant Ecology, 148, 105–114.

[ece39304-bib-0052] Husemann, M. , Schmitt, T. , Zachos, F. E. , Ulrich, W. , & Habel, J. C. (2014). Palaearctic biogeography revisited: Evidence for the existence of a north African refugium for western palaearctic biota. Journal of Biogeography, 41, 81–94.

[ece39304-bib-0053] Hutchinson, J. , Dalziel, J. , & Hepper, F. (1954–1972). In R. W. J. Keay & F. N. Hepper (Eds.), Flora of west tropical Africa (2nd ed.. Revised by). Crown Agent.

[ece39304-bib-0054] Ibarra Polesel, M. G. , & Damborsky, M. P. (2018). Changes in the structure of Melolonthidae (Coleoptera: Scarabaeoidea) assemblages along a temporal gradient in a natural reserve in Chaco, Argentina. Austral Entomology, 57, 377–386. 10.1111/aen.12288

[ece39304-bib-0055] Jaffre, T. , Morat, P. , & Veillon, J.‐M. (1994). Dossier nouvelle‐calédonie. La flore: Caractéristiques et composition floristique des principales formations végétales . Bois et forêts des tropiques – N°242 ‐ 4E Trimestre.

[ece39304-bib-0056] Jennings, R. , Finlayson, C. , Fa, D. , & Finlayson, G. (2011). Southern iberia as a refuge for the last neanderthal populations. Journal of Biogeography, 38, 1873–1885.

[ece39304-bib-0057] Jiménez‐Mejías, P. , Luceño, M. , Lye, K. A. , Brochmann, C. , & Gussarova, G. (2012). Genetically diverse but with surprisingly little geographical structure: The complex history of the widespread herb carex nigra (cyperaceae). Journal of Biogeography, 39, 2279–2291.

[ece39304-bib-0058] Kimpouni, V. , Apani, E. , & Motom, M. (2013). Analyse phytoécologique de la flore ligneuse de la haute sangha (république du congo). Adansonia, 35, 107–134.

[ece39304-bib-0506] Kindt, R. , & Coe, R. (2005). Tree diversity analysis. A manual and software for common statistical methods for ecological and biodiversity studies. World Agroforestry Centre (ICRAF).

[ece39304-bib-0059] Koenen, E. J. M. , Clarkson, J. J. , Pennington, T. D. , & Chatrou, L. W. (2015). Recently evolved diversity and convergent radiations of rainforest mahoganies (Meliaceae) shed new light on the origins of rainforest hyperdiversity. The New Phytologist, 207, 327–339. 10.1111/nph.13490 26053172

[ece39304-bib-0060] Koffi, K. G. , Hardy, O. J. , Doumenge, C. , Cruaud, C. , & Heuertz, M. (2011). Diversity gradients and phylogeographic patterns in santiria trimera (Burseraceae), a widespread African tree typical of mature rainforests. American Journal of Botany, 98, 254–264. 21613114 10.3732/ajb.1000220

[ece39304-bib-0061] Kokou, K. (1998). Les mosaïques forestières au sud du togo: Biodiversité, dynamique et activités humaines (p. 140). (PHD Thesis). University of Montpellier II.

[ece39304-bib-0062] Kokou, K. , Adjossou, K. , & Kokutse, A. D. (2008). Considering sacred and riverside forests in criteria and indicators of forest management in low wood producing countries: The case of Togo. Ecological Indicators, 8, 158–169.

[ece39304-bib-0509] Kokou, K. , Caballe, G. , Akpagana, K. , & Batawila, K. (1999). Les îlots forestiers au sud du Togo: Dynamique et relations avec les végétations périphériques. Revue d’Ecologie, Terre et Vie, Société Nationale de Protection de la Nature, 54(4), 301–314.

[ece39304-bib-0505] Kouamé, F. N. , Kouadio, K. E. , Kouassi, K. , & Poorter, L. (2004). Floristic diversity of closed forestsin Côte d'Ivoire. In L. Poorter , F. Bongers , F. N. Kouame , & W. D. Hawthorne (Eds.), Biodiversity of west africanforests: An ecological atlas of woody plant species (pp. 53–60). CABI Publishing.

[ece39304-bib-0063] Kuneš, P. , Pelánková, B. , Chytrý, M. , Jankovská, V. , Pokorný, P. , & Petr, L. (2008). Interpretation of the last‐glacial vegetation of Eastern‐Central Europe using modern analogues from Southern Siberia. Journal of Biogeography, 35, 2223–2236.

[ece39304-bib-0064] Langenberger, G. , Martin, K. , & Sauerborn, J. (2006). Vascular plant species inventory of a Philippine lowland rain forest and its conservation value. Biodiversity and Conservation, 15, 1271–1301.

[ece39304-bib-0065] Lebrun, J. P. , & Stork, A. L. (1991–1992). Enumération des plantes à fleurs d'afrique tropicale . Editions du Cons et Jard Bot de la ville de Geneve.

[ece39304-bib-0508] Lenormand, M. , Papuga, G. , Argagnon, O. , Soubeyrand, M. , De Barros, G. , Alleaume, S. , & Luque, S. (2019). Biogeographical network analysis of plant species distribution in the Mediterranean region. Ecology and Evolution, 9(1), 237–250. 30680110 10.1002/ece3.4718 PMC6342112

[ece39304-bib-0066] Ley, A. C. , Heuertz, M. , & Hardy, O. J. (2016). The evolutionary history of central African rain forest plants: Phylogeographical insights from sister species in the climber genus haumania (marantaceae). Journal of Biogeography, 44, 308–321.

[ece39304-bib-0068] Lieberman, D. , Lieberman, M. , Peralta, R. , & Hartshorn, G. S. (1996). Tropical forest structure and composition on a large‐scale altitudinal gradient in Costa Rica. Journal of Ecology, 84, 137–152.

[ece39304-bib-0069] Linder, H. P. (2001). Plant diversity and endemism in Sub‐Saharan tropical Africa. Journal of Biogeography, 28, 169–182.

[ece39304-bib-0070] Linder, H. P. , De Klerk, H. M. , Born, J. , Burgess, N. D. , Fjeldså, J. , & Rahbek, C. (2012). The partitioning of Africa: Statistically defined biogeographical regions in Sub‐Saharan Africa. Journal of Biogeography, 39, 1189–1205.

[ece39304-bib-0071] Maley, J. (1980). Les changements climatiques de la fin du Tertiaire en Afrique: Leur conséquence sur l'apparition du Sahara et de sa végétation = end‐Tertiary climatic changes in Africa: their effect on the origin of the Sahara and its vegetation. In M. A. J. Williams & H. Faure (Eds.), The Sahara and the Nile: Quaternary environments and prehistoric occupation in Northern Africa (pp. 63–86). Balkema.

[ece39304-bib-0072] Maley, J. (1989). Late quaternary climatic changes in the African rain forest: Forest refugia and the major role of sea surface temperature variations. In M. Leinen & M. Sarnthein (Eds.), Paleoclimatology and paleometeorology: Modern and past patterns of global atmospheric transport (pp. 585–616). Springer Netherlands.

[ece39304-bib-0073] Maley, J. (1991). The African rain forest vegetation and palaeoenvironments during late Quaternary. Climatic Change, 19(1), 79–98.

[ece39304-bib-0074] Maley, J. (1992). Mise en evidence d'une pejoration climatique entre ca. 2500 et 2000 ans BP en Afrique tropicale humide; commentaires sur la note de D. Schwartz. Bulletin de la Société Géologique de France, 163(3), 363–365.

[ece39304-bib-0075] Maley, J. (1996). The African rain forest: Main characteristics of changes in vegetation and climate from the Upper Cretaceous to the Quaternary. Proceedings of the Royal Society of Edinburgh. Section B: Biological Sciences, 104B, 31–73.

[ece39304-bib-0503] Maley, J. (1997). Middle to Late Holocene changes in tropical Africa and other continents: Paleomonsoon and sea surface temperature variations. In H. Niizhet Dalfes , G. Kukla , & H. Weiss (Eds.), Third millennium BC climate change and Old World collapse (pp. 611–640). Springer‐Verlag Berlin Heidelberg.

[ece39304-bib-0076] Maley, J. , & Brenac, P. (1998). Vegetation dynamics, palaeoenvironments and climatic changes in the forests of western cameroon during the last 28,000 years B.P. Review of Palaeobotany and Palynology, 99, 157–187.

[ece39304-bib-0077] Maley, J. (2001). Elaeis guineensis Jacq.(oil palm) fluctuations in central Africa during the late Holocene: Climate or human driving forces for this pioneering species? Vegetation History and Archaeobotany, 10(2), 117–120.

[ece39304-bib-0078] Margules, C. R. , & Pressey, R. L. (2000). Systematic conservation planning. Nature, 405, 243–253. 10821285 10.1038/35012251

[ece39304-bib-0079] Marshall, C. A. M. , Wieringa, J. J. , & Hawthorne, W. D. (2021). An interpolated biogeographical framework for tropical Africa using plant species distributions and the physical environment. Journal of Biogeography, 48, 23–36. 10.1111/jbi.13976

[ece39304-bib-0080] Mccune, B. , Grace, J. B. , & Urban, D. L. (2002). Analysis of ecological communities Gleneden Beach (Oregon ed.). MjM Software Design.

[ece39304-bib-0081] Meave, J. , & Kellman, M. (1994). Maintenance of rain forest diversity in riparian forests of tropical savannas: Implications for species conservation during pleistocene drought. Journal of Biogeography, 21, 121–135.

[ece39304-bib-0082] Meave, J. , Kellman, M. , Macdougall, A. , & Rosales, J. (1991). Riparian habitats as tropical forest refugia. Global Ecology and Biogeography Letters, 1, 69–76.

[ece39304-bib-0083] Médail, F. , & Diadema, K. (2009). Glacial refugia influence plant diversity patterns in the mediterranean basin. Journal of Biogeography, 36, 1333–1345.

[ece39304-bib-0084] Montade, V. , Ledru, M.‐P. , Burte, J. , Martins, E. S. P. R. , Verola, C. F. , Costa, I. R. D. , Magalhães, E. , & Silva, F. H. (2014). Stability of a neotropical microrefugium during climatic instability. Journal of Biogeography, 41, 1215–1226.

[ece39304-bib-0085] Mueller‐Dombois, D. , & Ellenberg, H. (1974). Aims and methods of vegetation ecology. Wiley and Sons.

[ece39304-bib-0086] Naiman, R. J. , Decamps, H. , & Pollock, M. (1993). The role of riparian corridors in maintaining regional biodiversity. Ecological Applications, 3, 209–212. 27759328 10.2307/1941822

[ece39304-bib-0087] Natta, A. (2003) Ecological assessment of riparian forests in Benin: Phytodiversity, phytosociology, and spatial distribution of tree species (p. 189). (PHD Thesis). Wageningen University.

[ece39304-bib-0088] Nicolas, V. , Missoup, A. D. , Denys, C. , Kerbis Peterhans, J. , Katuala, P. , Couloux, A. , & Colyn, M. (2010). The roles of rivers and pleistocene refugia in shaping genetic diversity in praomys misonnei in tropical Africa. Journal of Biogeography, 38, 191–207.

[ece39304-bib-0089] Ngomanda, A. , Chepstow‐Lusty, A. , Makaya, M. , Schevin, P. , Maley, J. , Fontugne, M. , Oslisly, R. , Rabenkogo, N. , & Jolly, D. (2005). Vegetation changes during the past 1300 years in western equatorial Afnrca: A highresolution pollen record from Lake Kamalee, Lope Reserve, Central Gabon. The Holocene, 15(7), 1021–1031.

[ece39304-bib-0090] Noss, R. F. (2001). Beyond kyoto: Forest management in a time of rapid climate change después de kyoto: Manejo forestal en tiempos de cambio climático acelerado. Conservation Biology, 15, 578–590.

[ece39304-bib-0091] Papadakis, J. (1966). Enquete agro‐ecologique en afrique occidentale/liberia, Côte‐D'ivoire, Ghana, Togo, Dahomey, Nigeria (p. 43). FAO.

[ece39304-bib-0107] Pennington, T. R. , Prado, D. E. , & Pendry, C. A. (2000). Neotropical seasonally dry forests and quaternary vegetation changes. Journal of Biogeography, 27, 261–273.

[ece39304-bib-0510] Pither, R. , & Kellman, M. (2002). Tree species diversity in small tropical riparian forest fragments in Belize, Central America. Biodiversity and Conservation, 11, 1623–1636.

[ece39304-bib-0092] Plana, V. , Gascoigne, A. , Forrest, L. L. , Harris, D. , & Pennington, R. T. (2004). Pleistocene and pre‐Pleistocene Begonia speciation in Africa. Molecular Phylogenetics and Evolution, 31(2), 449–461. 15062787 10.1016/j.ympev.2003.08.023

[ece39304-bib-0504] Poorter, L. , Bongers, F. , & Lemmens, R. H. M. J. (2004). West African forests: Introduction. In L. Poorter , F. Bongers , F. N. Kouame , & W. D. Hawthorne (Eds.), Biodiversity of westafricanforests: An ecological atlas of woody plant species (p. 5). CABI Publishing.

[ece39304-bib-0094] Reynaud‐Farrera, I. , Maley, J. , & Wirrmann, D. (1996). Végétation et climat dans les forêts du Sud‐Ouest Cameroun depuis 4770 ans BP: Analyse pollinique des sédiments du Lac Ossa. Comptes Rendus. Académie des Sciences, 322 (série II a), 749–755.

[ece39304-bib-0095] R Development Core Team . (2011). R: A language and environment for Statistical Computing. Toulouse. Available at:. https//www.R‐project.org/

[ece39304-bib-0096] Rull, V. (2005). Palaeovegetational and palaeoenvironmental trends in the summit of the guaiquinima massif (Venezuelan Guayana) during the holocene. Journal of Quaternary Science, 20, 135–145.

[ece39304-bib-0097] Russell, D. A. , Rich, F. J. , Schneider, V. , & Lynch‐Stieglitz, J. (2009). A warm thermal enclave in the late pleistocene of the south‐eastern United States. Biological Reviews, 84, 173–202. 19391200 10.1111/j.1469-185x.2008.00069.x

[ece39304-bib-0098] Salanville, P. (1992). Changements climatiques dans la péninsule arabique durant le Pléistocène supérieur et l'Holocène. Paléorient, 18‐1, 5–26.

[ece39304-bib-0099] Schwartz, D. , Guillet, B. , & Dechamps, R. (1990). Etude de deux flores forestières mi‐holocène (6000–3000 BP) et subactuelle (500 BP) conservées in situ sur le littoral pontenegrin (Congo). In L. Raymond & S. Dominique (Eds.), Paysages quaternaires de l'Afrique centrale atlantique (pp. 283–297). ORSTOM (Didactiques).

[ece39304-bib-0100] Selwood, K. E. , & Zimmern, H. C. (2020). Refuges for biodiversity conservation: a review of the evidence. Biological Conservation, 245, 108502. 10.1016/j.biocon.2020.108502

[ece39304-bib-0507] Shannon, C. E. , & Weaver, W. (1949). The mathematical theory of communication. University of Illinois Press.

[ece39304-bib-0101] Silva de Miranda, P. L. , Oliveira‐Filho, A. , Pennington, R. T. , Neves, D. M. , Baker, T. R. , & Dexter, K. G. (2018). Using tree species inventories to map biomes and assess their climatic overlaps in lowland tropical South America. Global Ecology and Biogeography, 27, 899–912. 10.1111/geb.12749

[ece39304-bib-0102] Sosef, M.S.M. (1994). Refuge Begonias. Taxonomy, Phylogeny and Historical Biogeography of Begonia sect. Loasibegonia and sect. Scutobegonia in Relation to Glacial Rain Forest Refuges in Africa . (PhD Thesis), Wageningen Agricultural University.

[ece39304-bib-0103] Sosef, M. , Issembe, Y. , Bourbou, H. P. , & Koopman, W. J. M. (2004). Botanical diversity of the pleistocene forest refuge Monts Doudou. California Academy of Sciences Memoir, 28, 17–91.

[ece39304-bib-0104] Souwnmi, M. A. (1999). The significance of the oil palm (*Elaeis guineensis* Jacq.) in the late Holocene environments of west and west central Africa: A further consideration. Vegetation History and Archaeobotany, 8(3), 199–210.

[ece39304-bib-0105] Terral, J. F. , Badal, E. , Heinz, C. , Roiron, P. , Thiebault, S. , & Figueiral, I. (2004). A hydraulic conductivity model points to post‐neogene survival of the mediterranean olive. Ecology, 85, 3158–3165.

[ece39304-bib-0106] Tibby, J. , Barr, C. , Marshall, J. C. , Mcgregor, G. B. , Moss, P. T. , Arnold, L. J. , Page, T. J. , Questiaux, D. , Olley, J. , Kemp, J. , Spooner, N. , Petherick, L. , Penny, D. , Mooney, S. , & Moss, E. (2017). Persistence of wetlands on north stradbroke island (south‐east Queensland, Australia) during the last glacial cycle: Implications for quaternary science and biogeography. Journal of Quaternary Science, 32, 770–781.

[ece39304-bib-0108] Tchouto, M. G. P. , de Wilde, J. J. F. E. , de Boer, W. F. , van der Maesen, L. J. G. , & Cleef, A. M. (2009). Bio‐indicator species and Central African rain forest refuges in the Campo‐Ma'an area, Cameroon. Systematics and Biodiversity, 7(1), 21–31. 10.1017/S1477200008002892

[ece39304-bib-0109] Ter Steege, H. , Pitman, N. C. , Sabatier, D. , Baraloto, C. , Salomão, R. P. , Guevara, J. E. , Phillips, O. L. , Castilho, C. V. , Magnusson, W. E. , Molino, J. F. , & Monteagudo, A. (2013). Hyperdominance in the Amazonian Tree Flora. Science, 342, 1243092. 10.1126/science.1243092 24136971

[ece39304-bib-0110] Uhl, C. , & Murphy, P. G. (1981). Composition structure and regeneration of a tierra firme forest in the amazon basin of Venezuela. Tropical Ecology, 22, 219–237.

[ece39304-bib-0111] Vincens, A. , Buchet, G. , Elenga, H. , Fournier, M. , Martin, L. , de Namur, C. , Schwartz, D. , Servant, M. , & Wirrmann, D. (1994). Changement majeur de la végétation du lac Sinnda (vallée du Niari, Sud Congo) consécutif à l'assèchement climatique holocène supérieur: Apport de la palynologie. Comptes Rendus à l'Academie des Sciences de Paris, 318, 1521–1526.

[ece39304-bib-0112] Vincens, A. , Schwartz, D. , Bertau, J. , Elenga, H. , & de Namur, C. (1998). Late holocene climatic changes in western equatorial Africa inferred from pollen from Lake Sinnda, Southern Congo. Quaternary Research, 50, 34–45.

[ece39304-bib-0113] White, F. (1983). In Unesco (Ed.), The vegetation of Africa (p. 357). Courvoisier S.A.

[ece39304-bib-0114] Whittaker, R. H. (1972). Evolution and measurement of species diversity. Taxon, 21, 213–251.

[ece39304-bib-0115] Wieringa, J. J. , & Poorter, L. (2004). Biodiversity hotspots in West Africa; patterns and causes. In L. Poorter , F. Bongers , F. N. Kouame´ , & W. D. Hawthorne (Eds.), Biodiversity of west African forests: An ecological atlas of woody plant species (pp. 61–72). CABI Publishing.

[ece39304-bib-0116] Wright, H. E. , McCullough, J. , Alonso, L. E. , & Diallo, M. S. (Eds.). (2006). Une Évaluation Biologique Rapide de Trois Forêt Classées du Sud‐est de la Guinée. Bulletin RAP d'Evaluation Rapide 40. Conservation International.

