# Supplementary material for: Floristic composition and turnover analysis in Dahomey Gap and the surrounding sub‐humid Togolese mountain minor forest refuges: Importance for biogeography and biodiversity conservation in sub‐Saharan Africa
Source: Ecol Evol. 2022 Oct 4;12(10):e9304. doi: 10.1002/ece3.9304 (PMC9532220; doi:10.1002/ece3.9304)
Supplement: Supplementary file 1 — Appendix S1 [file ECE3-12-e9304-s001.docx]

Appendix. S1 Dahomey gap minor forest refuges Togo flora : nomber (N=868) , name of species including authority, classical botanical family (Cronquist) and following APGIII (Angiosperm Phylogeny Group, 2009); phytogeographical origin **(Phyto)** : Cosmopolitan **(Cosm)**, Pantropical (**Pan)**, Paleotropical **(Pal),** Afro-American (**AA**), Afro-Malagasy (**AM**), Guinean-Congolese regional centre of endemism (**GC**), Upper Guinean subcentre of endemism including Western Guinean-Congolese forest block (**GCW**), Lower Guinea and Congolese sub-centre of endemism (**GCE**), Dahomey Gap (**DG**), Sudanese-zambezian regional centre of endemism (**SZ**), Guineo-Congolian-Zambezi regional transition zone and Guineo-Congolian-Sudanese (**GC-SZ**), Introduced origine (**I**), Indeterminacy (**Ind**) ; sites considered as major forest refuges in sub-Saharan tropical Africa, including the Taï Forest Reserve located between southwest Côte d'Ivoire **(CI)** and Liberia near the large forest refuges of Palmas Cape and the Nimba Mountains, the Dahomey Gap**( DG**) vegetation sensu stricto located between Togo and Benin, part of the large forest refuges between Ghana and Benin and the Haute Sangha forest refuges in Democratic Republic of Congo (**RDC**); previous inventories in non-riparian forests only, i.e., "Terre firme" forests **(TF)** and vegetation matrix of whole forests (**ALL**); (+): presence , (-): absent

| **N** | ***Species*** | **Cronquist** | **APGIII** | **Phyto** | **C I** | **Benin** | **RDC** | **DG** | **TF** | **ALL** |
| --- | --- | --- | --- | --- | --- | --- | --- | --- | --- | --- |
| 1 | *Abrus canescens Welw. ex Bak.* | Papilionaceae | Fabaceae | AT | - | + | - | + | + | + |
| 2 | *Abrus precatorius Linn.* | Papilionaceae | Fabaceae | Pan | + | + | - | + | + | + |
| 3 | *Abrus pulchellus Wall. ex Thw.* | Papilionaceae | Fabaceae | Paleo | - | + | - | + | + | + |
| 4 | *Abutilon mauritianum (Jac.) Medic.* | Malvaceae | Malvaceae | AT | + | + | - | + | - | + |
| 5 | *Acacia pennata Willd.* | Mimosaceae | Fabaceae | GC | + | - | - | + | + | + |
| 6 | *Acalypha racemosa Wall.ex Baill.* | Euphorbiaceae | Euphorbiaceae | Paleo | - | - | - | + | + | + |
| 7 | *Acanthus guineensis Heine & P. Taylor* | Acanthaceae | Acanthaceae | GCW | - | - | - | - | - | + |
| 8 | *Achyranthes aspera Linn.* | Amaranthaceae | Amaranthaceae | Pan | - | + | - | + | + | + |
| 9 | *Acridocarpus smeathmannii (DC.) Guill. & Perr.* | Malpighiaceae | Malpighiaceae | AT | - | - | - | + | + | + |
| 10 | *Acroceras gabunense (Hack.) W.D.Clayton* | Gramineae | Gramineae | GC | - | - | - | + | - | + |
| 11 | *Acroceras zizanioides (Kunth.) Dandy* | Gramineae | Gramineae | Pan | - | + | - | + | - | + |
| 12 | *Adenia cissampeloides (Planch.ex Hook.) Harms* | Passifloraceae | Passifloraceae | GC | + | + | - | + | + | + |
| 13 | *Adenia guineensis W. de Wilde* | Passifloraceae | Passifloraceae | GC | - | - | - | - | + | + |
| 14 | *Adenia lobata (Jacq.) Engl.* | Passifloraceae | Passifloraceae | GC | + | + | - | + | + | + |
| 15 | *Adenia rumicifolia Engl & Harms* | Passifloraceae | Passifloraceae | AT | - | + | - | - | + | + |
| 16 | *Adiantum phillipense Linn.* | Adiantaceae | Pteridaceae | Paleo | - | + | - | - | + | + |
| 17 | *Adiantum schweinfurthii Kuhn* | Adiantaceae | Pteridaceae | SZ | - | - | - | - | + | + |
| 18 | *Adiantum vogelii Mett. ex Keys.* | Adiantaceae | Pteridaceae | GC | + | - | - | - | + | + |
| 19 | *Aframomum daniellii (Hook. F.) K. Schum.* | Zingiberaceae | Zingiberaceae | GC | - | - | - | - | + | + |
| 20 | *Aframomum sceptrum (Oliv. & Hanb.) K.Schum.* | Zingiberaceae | Zingiberaceae | GC | + | + | - | + | + | + |
| 21 | *Afrosersalisia afzelii (Engl.) A. Chev. = Synsepalum afzelii* | Sapotaceae | Sapotaceae | GC | + | - | - | - | + | + |
| 22 | *Afzelia africana Sm.* | Caesalpiniaceae | Fabaceae | AT | - | + | - | + | + | + |
| 23 | *Afzelia bella Harms var.gracilior Keay* | Caesalpiniaceae | Fabaceae | GCW | + | - | - | - | - | + |
| 24 | *Afzelia bracteata vogel ex Benth.* | Caesalpiniaceae | Fabaceae | GCW | - | - | - | - | + | + |
| 25 | *Agelaea obliqua (P. Beauv.) Baill.=A. pentagyna* | Connaraceae | Connaraceae | GC | + | - | - | + | + | + |
| 26 | *Ageratum conyzoides Linn.* | Asteraceae | Asteraceae | Pan | + | + | - | + | + | + |
| 27 | *Aidia genipiflora (D.C.) Dandy* | Rubiaceae | Rubiaceae | GC | + | - | - | - | + | + |
| 28 | *Alafia barteri Oliv.* | Apocynaceae | Apocynaceae | GC | - | + | - | + | + | + |
| 29 | *Alafia scandens (Thonn.) De Wild.* | Apocynaceae | Apocynaceae | GC | - | + | - | - | + | + |
| 30 | *Albizia adianthifolia (Schum.) W. F. Wright* | Mimosaceae | Fabaceae | GC | + | - | + | + | + | + |
| 31 | *Albizia coriaria Welw.ex Oliv.* | Mimosaceae | Fabaceae | AT | - | + | - | - | - | + |
| 32 | *Albizia ferruginea (Guill.& Perr.)Benth.* | Mimosaceae | Fabaceae | GC | + | + | + | + | + | + |
| 33 | *Albizia glaberrima (Schum. & Thonn.) Benth.* | Mimosaceae | Fabaceae | GC | - | + | + | + | + | + |
| 34 | *Albizia lebbeck (Linn.) Benth.* | Mimosaceae | Fabaceae | Pan | - | - | - | - | - | + |
| 35 | *Albizia zygia (DC.) J.F. Macbr.* | Mimosaceae | Fabaceae | GC | + | - | - | + | + | + |
| 36 | *Alchornea cordifolia (Schum. & Thonn.) Müll. Arg.* | Euphorbiaceae | Euphorbiaceae | AT | + | + | + | + | + | + |
| 37 | *Alchornea floribunda Müll.Arg.* | Euphorbiaceae | Euphorbiaceae | GC | + | - | - | - | - | + |
| 38 | *Allophyllus africanus P. Beauv.* | Sapindaceae | Sapindaceae | AT | + | + | + | + | + | + |
| 39 | *Allophyllus spicatus (Poir.) Radlk.* | Sapindaceae | Sapindaceae | AT | - | + | - | + | + | + |
| 40 | *Allophyllus talbotii Beker f.* | Sapindaceae | Sapindaceae | GC | - | - | - | - | + | + |
| 41 | *Alstonia boonei De Wild.* | Apocynaceae | Apocynaceae | GC | + | - | + | + | + | + |
| 42 | *Amorphophallus accrensis N.E.Br.= A. johnsonii* | Araceae | Araceae | GCW | + | - | - | + | + | + |
| 43 | *Amorphophallus flavovirens N. E. Br.* | Araceae | Araceae | AT | - | + | - | - | - | + |
| 44 | *Ampelocissus bombycina (Bak.) Planch.* | Vitaceae | Vitaceae | AT | - | + | - | + | + | + |
| 45 | *Ampelocissus multistriata (Baker) Planch* | Vitaceae | Vitaceae | AT | - | + | - | - | + | + |
| 46 | *Amphimas pterocarpoïdes Harms* | Caesalpiniaceae | Fabaceae | GC | + | - | + | - | + | + |
| 47 | *Ananas comosus (Linn.) Merr.* | Bromeliaceae | Bromeliaceae | I | - | - | - | + | + | + |
| 48 | *Anchomanes difformis (Bl.) Engl.* | Araceae | Araceae | GC | + | + | - | + | + | + |
| 49 | *Ancistrophyllum secundiflorum (P. Beauv.) Wendl. = Laccosperma scundiflorum* | Arecaceae | Arecaceae | GC | + | - | - | - | + | + |
| 50 | *Ancistrophyllum spp = Laccosperma spp* | Arecaceae | Arecaceae | Ind | - | - | - | - | - | + |
| 51 | *Ancylobotrys amoena Hua* | Apocynaceae | Apocynaceae | AT | - | - | - | - | + | + |
| 52 | *Andropogon gayanus Kunth var. gayanus* | Gramineae | Gramineae | AT | - | + | - | + | - | + |
| 53 | *Andropogon macrophyllus Stapf.* | Gramineae | Gramineae | SZ | - | + | - | - | + | + |
| 54 | *Andropogon tectorum Schum. & Thonn.* | Gramineae | Gramineae | SZ | - | + | - | + | + | + |
| 55 | *Aneilema beninense (P. Beauv.) Kunth* | Commelinaceae | Commelinaceae | GC | - | + | - | + | + | + |
| 56 | *Aneilema dispermum Brenan* | Commelinaceae | Commelinaceae | GC | - | - | - | - | + | + |
| 57 | *Aneilema umbrosum subesp. ovato-oblongum (P. Beauv.) Morton* | Commelinaceae | Commelinaceae | GC | - | - | - | + | + | + |
| 58 | *Aningeria altissima (A. Chev.) Aubrév. et Pellegr.* | Sapotaceae | Sapotaceae | GC | - | - | + | - | + | + |
| 59 | *Anogeissus leiocarpus (DC.) Guill. & perr.* | Combretaceae | Combretaceae | SZ | - | + | - | + | + | + |
| 60 | *Anthocleista djalonensis A. Chev.* | Loganiaceae | Gentianaceae | AT | - | + | - | + | + | + |
| 61 | *Anthocleista nobilis G. Don* | Loganiaceae | Gentianaceae | GCW | + | + | - | - | + | + |
| 62 | *Anthocleista vogelii Planch.* | Loganiaceae | Gentianaceae | GC | + | + | + | + | + | + |
| 63 | *Antiaris africana Engl. = A. toxicaria* | Moraceae | Moraceae | AT | + | - | + | + | + | + |
| 64 | *Antidesma laciniatum Mül. Arg. var. membranaceum Müll. Arg.* | Euphorbiaceae | Phyllantaceae | GC | - | - | + | - | + | + |
| 65 | *Antidesma membranaceum Müll. Arg.* | Euphorbiaceae | Phyllantaceae | GC | + | + | + | + | + | + |
| 66 | *Antidesma vernosum F.T.A.* | Euphorbiaceae | Phyllantaceae | AT | - | + | - | - | + | + |
| 67 | *Anubias gigantea A. Chev.ex Hutch.* | Araceae | Araceae | GCW | - | - | - | - | - | + |
| 68 | *Anubias hastifolus Engel* | Araceae | Araceae | GC | - | - | - | - | - | + |
| 69 | *Aphanostylis mannii (Stapf) Pierre* | Apocynaceae | Apocynaceae | GC | - | - | - | - | + | + |
| 70 | *Aphania senegalensis (Juss.ex Poir.) Radlk.* | Sapindaceae | Sapindaceae | GC | + | + | - | + | + | + |
| 71 | *Argocoffeopsis jasminoides (Welw. ex Hiern) Robbrecht* | Rubiaceae | Rubiaceae | GC | - | - | - | - | + | + |
| 72 | *Argocoffeopsis rupestris (Hiern)(Robbrecht)* | Rubiaceae | Rubiaceae | GC | + | - | - | + | + | + |
| 73 | *Artabotrys velutinus Sc. Elliot* | Annonaceae | Annonaceae | GC | - | + | - | + | + | + |
| 74 | *Artocarpus altilis (Parkinson)Fosberg* | Moraceae | Moraceae | I | - | - | - | - | - | + |
| 75 | *Asparagus africanus Lam.* | Liliaceae | Asparagaceae | SZ | - | + | - | + | + | + |
| 76 | *Asparagus flagellaris (Kunth) Bak.* | Liliaceae | Asparagaceae | SZ | - | - | - | + | - | + |
| 77 | *Asparagus warneckei (Engl.) Hutch.* | Liliaceae | Asparagaceae | GC | - | - | - | + | - | + |
| 78 | *Aspilia paludosa Berhaut* | Asteraceae | Asteraceae | SZ | - | + | - | - | - | + |
| 79 | *Asplenium buettneri Hieron.ex Brause* | Aspleniaceae | Aspleniaceae | GC | - | - | - | - | - | + |
| 80 | *Asplenium emarginatum P. Beauv.* | Aspleniaceae | Aspleniaceae | GC | - | - | - | + | + | + |
| 81 | *Asplenium formosum Willd.* | Aspleniaceae | Aspleniaceae | Paleo | - | - | - | - | + | + |
| 82 | *Asplenium hemitomum Hieron.* | Aspleniaceae | Aspleniaceae | GC | - | - | - | - | - | + |
| 83 | *Asystasia gangetica (Linn.) T. Anders.* | Acanthaceae | Acanthaceae | Pan | + | + | - | + | + | + |
| 84 | *Asystasia scandens (Lindau)Hooker* | Acanthaceae | Acanthaceae | Paleo | - | - | - | - | + | + |
| 85 | *Asystasia vogeliana Benth.* | Acanthaceae | Acanthaceae | GC | + | - | - | - | + | + |
| 86 | *Aubrevillea kerstingii (Harms) Pellegr.* | Mimosaceae | Fabaceae | GC | + | - | + | - | + | + |
| 87 | *Baissea leonensis Benth.* | Apocynaceae | Apocynaceae | GC | - | - | - | + | + | + |
| 88 | *Baissea multiflora A. DC.* | Apocynaceae | Apocynaceae | AT | + | - | - | + | - | + |
| 89 | *Baissea zygodioides (K. Schum.) Staf* | Apocynaceae | Apocynaceae | GCW | + | - | - | + | + | + |
| 90 | *Bambusa vulgaris Schrzd ex Wendel* | Gramineae | Gramineae | I | - | + | - | + | + | + |
| 91 | *Baphia nitida Lodd.* | Papilionaceae | Fabaceae | GC | + | - | - | + | + | + |
| 92 | *Baphia pubescens Hook.f.= B. bancoensis* | Papilionaceae | Fabaceae | GC | + | - | - | + | + | + |
| 93 | *Belonophora hypoglauca (Welw.ex Hiern)Dawson&Cheek* | Rubiaceae | Rubiaceae | GC | - | - | - | - | + | + |
| 94 | *Bequaertiodendron oblanceolatum (S. Moore) Heine= Englerophylum oblanceolatum* | Sapotaceae | Sapotaceae | GC | - | - | - | - | + | + |
| 95 | *Berlinia grandiflora (Valh) Hutch. & Dalz.* | Caesalpiniaceae | Fabaceae | AT | + | + | + | + | - | + |
| 96 | *Bersama abyssinica Fres.Subsp.Paollinioides* | Melianthaceae | Melianthaceae | GC | - | - | - | - | + | + |
| 97 | *Bertiera bracteolata Hiern* | Rubiaceae | Rubiaceae | GC | + | - | - | - | - | + |
| 98 | *Bertiera racemosa (D. Don.) K. Schum.* | Rubiaceae | Rubiaceae | GC | + | - | - | - | + | + |
| 99 | *Bidens pilosa Linn.* | Asteraceae | Asteraceae | I | - | + | - | + | - | + |
| 100 | *Blepharis maderaspatensis (Linn.) Heyne ex Roth.* | Acanthaceae | Acanthaceae | Paleo | - | + | - | + | - | + |
| 101 | *Blighia sapida Konig* | Sapindaceae | Sapindaceae | Pan | - | + | - | + | + | + |
| 102 | *Blighia unijugata Bak.* | Sapindaceae | Sapindaceae | GC | + | + | - | + | + | + |
| 103 | *Blighia welwitschii (Hiern) Radlk* | Sapindaceae | Sapindaceae | GC | + | - | + | - | + | + |
| 104 | *Boehmeria macrophylla Hornem* | Urticaceae | Urticaceae | Paleo | - | - | - | - | - | + |
| 105 | *Bolbitis acrostichoïdes (Afzel. ex Sw.) Ching* | Lomariopsidaceae | Dryopteridaceae | GC | + | - | - | - | - | + |
| 106 | *Bolbitis heudoletii (Bory ex Fée) Alston* | Lomariopsidaceae | Dryopteridaceae | GC | - | + | - | - | - | + |
| 107 | *Bombax buonopozense P. Beauv.* | Bombacaceae | Malvaceae | GC | + | - | + | - | + | + |
| 108 | *Breonadia salicina (Vahl)Hepper &Wood* | Rubiaceae | Rubiaceae | SZ | - | - | - | - | - | + |
| 109 | *Bridelia atroviridis Müll. Arg.* | Euphorbiaceae | Phyllantaceae | GC | - | + | - | - | + | + |
| 110 | *Bridelia ferruginea Benth.* | Euphorbiaceae | Phyllantaceae | AT | - | + | - | + | + | + |
| 111 | *Bridelia micrantha (Hochst)Baill.* | Euphorbiaceae | Phyllantaceae | GC | - | + | + | - | + | + |
| 112 | *Brillantaisia lamium (Nees)Benth.* | Acanthaceae | Acanthaceae | GC | + | - | - | - | + | + |
| 113 | *Brillantaisia owariensis P.Beauv.* | Acanthaceae | Acanthaceae | GC | - | - | - | - | + | + |
| 114 | *Bulbophyllum congolanum Schiltr.* | Orchidaceae | Orchidaceae | GC | - | - | - | - | - | + |
| 115 | *Byrsocarpus coccineus Schium. & Thonn. = Rourea coccinea* | Connaraceae | Connaraceae | GC | + | + | - | + | + | + |
| 116 | *Caesalpinia pulcherrima Linn.* | Caesalpiniaceae | Fabaceae | I | - | - | - | + | + | + |
| 117 | *Caladium bicolor (Ait.) Vent* | Araceae | Araceae | I | - | - | - | - | - | + |
| 118 | *Calliandra portoricensis (Jacq.) Benth.* | Mimosaceae | Fabaceae | I | - | - | - | + | + | + |
| 119 | *Caloncoba echinata (Oliv.) Gilg.* | Flacourtiaceae | Salicaceae | GCW | - | - | - | - | - | + |
| 120 | *Caloncoba gilgiana (Sprague )Gilg* | Flacourtiaceae | Salicaceae | GC | - | - | - | + | - | + |
| 121 | *Calopogonium mucunoides Desv.* | Papilionaceae | Fabaceae | I | - | - | - | + | - | + |
| 122 | *Caloncoba wilwitschii (Oliv.) Gilg.* | Flacourtiaceae | Salicaceae | GC | - | - | + | - | - | + |
| 123 | *Calyptrochilum emarginatum (Sw.) Schltr.* | Orchidaceae | Orchidaceae | GC | - | + | - | + | - | + |
| 124 | *Campylospermum flavum (Schum. & Thonn. ex Stapf) Farron* | Ochnaceae | Ochnaceae | GC | + | + | - | + | + | + |
| 125 | *Campylospermum myrioneura Gilg.* | Ochnaceae | Ochnaceae | GC | - | - | - | - | + | + |
| 126 | *Canarium schweinfurthii Engel.* | Burseraceae | Burseraceae | GC | + | - | + | - | + | + |
| 127 | *Canthium cornelia Cham.* | Rubiaceae | Rubiaceae | SZ | - | + | - | - | - | + |
| 128 | *Canthium hispidum Benth. = Keetia hispida* | Rubiaceae | Rubiaceae | GC | + | + | - | + | - | + |
| 129 | *Canthium horizontale (Schum. & Thonn.) Hiern= Psydrax horizontalis* | Rubiaceae | Rubiaceae | AT | + | - | - | + | + | + |
| 130 | *Canthium multiflorum (Schum. & Thonn.) Hiern = Keetia multiflora* | Rubiaceae | Rubiaceae | GC | + | + | - | + | + | + |
| 131 | *Canthium pobeguinii Hutch. et Dalz* | Rubiaceae | Rubiaceae | GCW | - | - | - | - | + | + |
| 132 | *Canthium schimperianum A. Rich.* | Rubiaceae | Rubiaceae | AT | - | - | - | + | + | + |
| 133 | *Canthium subcordatum DC. = Psydrax subcordata* | Rubiaceae | Rubiaceae | GC | + | - | - | - | + | + |
| 134 | *Canthium vernosum (Oliv.)Hiern.* | Rubiaceae | Rubiaceae | AT | - | + | - | - | + | + |
| 135 | *Canthium vulgare (K. Schum.) Bullock* | Rubiaceae | Rubiaceae | GC | - | - | - | + | - | + |
| 136 | *Capparis erythrocarpos Isert* | Capparaceae | Capparaceae | GC | - | - | - | + | - | + |
| 137 | *Capsicum annuum Linn.* | Solanaceae | Solanaceae | Pan | - | + | - | + | + | + |
| 138 | *Carapa procera DC.* | Meliaceae | Meliaceae | AA | + | - | + | - | - | + |
| 139 | *Cardiospermum grandiflorum Swartz* | Sapindaceae | Sapindaceae | AA | - | - | - | + | + | + |
| 140 | *Carisa edulis Vahl* | Apocynaceae | Apocynaceae | Paleo | - | - | + | + | + | + |
| 141 | *Carica papaya Linn* | Caricaceae | Caricaceae | I | - | + | - | + | + | + |
| 142 | *Carpolobia lutea G. Don.* | Polygalaceae | Polygalaceae | GC | + | - | - | + | + | + |
| 143 | *Cassia alata Linn.= Senna alata* | Caesalpiniaceae | Fabaceae | Pan | - | + | - | - | + | + |
| 144 | *Cassia hirsuta Linn.= Senna hirsuta* | Caesalpiniaceae | Fabaceae | AA | - | + | - | + | - | + |
| 145 | *Cassia occidentalis Linn.= Senna accidentalis* | Caesalpiniaceae | Fabaceae | Pan | - | + | - | + | + | + |
| 146 | *Cassia siamea Lam. = Senna siamea* | Caesalpiniaceae | Fabaceae | I | - | - | - | + | + | + |
| 147 | *Cassia sieberiana DC.* | Caesalpiniaceae | Fabaceae | AT | - | + | - | + | + | + |
| 148 | *Cassia tora Linn.= Senna tora* | Caesalpiniaceae | Fabaceae | Pan | - | - | - | + | - | + |
| 149 | *Cathormion altissimum (Hook.f.)Hutch. & Dandy* | Mimosaceae | Fabaceae | GC | - | - | + | + | - | + |
| 150 | *Cayratia gracilis (Guill. & Perr.) Suessenguth* | Vitaceae | Vitaceae | GC | - | - | - | + | + | + |
| 151 | *Cayratia ibuensis (Hook.f.) Suessenguth* | Vitaceae | Vitaceae | AT | - | - | - | + | - | + |
| 152 | *Cedrela odorata Linn.* | Meliaceae | Meliaceae | I | - | - | - | - | + | + |
| 153 | *Ceiba pentandra (Linn.) Gaerth.* | Bombacaceae | Malvaceae | Pan | + | + | + | + | + | + |
| 154 | *Celosia isertii C. Towns.* | Amaranthaceae | Amaranthaceae | GC | - | - | - | + | + | + |
| 155 | *Celosia trigyna Linn.* | Amaranthaceae | Amaranthaceae | AM | - | + | - | + | - | + |
| 156 | *Celtis adolfi-friderici Engel.* | Ulmaceae | Canabaceae | GC | + | - | + | - | - | + |
| 157 | *Celtis integrifolia (Forssk)Hepper&Wood.= C. toka* | Ulmaceae | Canabaceae | SZ | - | + | - | - | - | + |
| 158 | *Celtis mildbraedii Engl.* | Ulmaceae | Canabaceae | GC | + | - | + | - | + | + |
| 159 | *Celtis philippensis Blanco* | Ulmaceae | Canabaceae | AT | - | - | + | + | + | + |
| 160 | *Celtis zenkeri Engl.* | Ulmaceae | Canabaceae | GC | + | - | - | + | + | + |
| 161 | *Centrosema pubescens Benth.* | Papilionaceae | Fabaceae | I | - | + | - | + | + | + |
| 162 | *Cercestis afzelii Schott.* | Araceae | Araceae | GC | + | - | - | - | - | + |
| 163 | *Ceropegia aristolochioides Hutch. & Dalziel* | Asclepiadaceae | Apocynaceae | AT | - | - | - | + | - | + |
| 164 | *Chaetacme aristata Planch.* | Ulmaceae | Ulmaceae | GC | - | - | - | + | + | + |
| 165 | *Chasmanthera dependens hochst.* | Menispermaceae | Menispermaceae | AT | - | + | - | + | + | + |
| 166 | *Chassalia kolly (Schumach.) Hepper* | Rubiaceae | Rubiaceae | GC | + | + | - | + | + | + |
| 167 | *Chlorophytum macrophyllum (A. Rich.) Aeschers* | Liliaceae | Asparagaceae | GC | - | + | - | + | - | + |
| 168 | *Chloris robusta Stapf.* | Gramineae | Gramineae | AT | - | - | - | - | - | + |
| 169 | *Chlorophytum togoense Engl.* | Liliaceae | Asparagaceae | AT | - | + | - | + | + | + |
| 170 | *Christiana africana A. DC.* | Tiliaceae | Malvaceae | GC | - | - | - | + | + | + |
| 171 | *Chrysophyllum africanum A. DC. var. Aubrevillei Pellegr.* | Sapotaceae | Sapotaceae | GC | - | - | + | - | + | + |
| 172 | *Chytranthus macrobotrys (Gilg)Excell&Mendonça* | Sapindaceae | Sapindaceae | GC | + | - | - | - | + | + |
| 173 | *Cissus aralioides (Welw.ex bak.) Planch.* | Vitaceae | Vitaceae | AT | + | + | - | + | + | + |
| 174 | *Cissus petiolata Hook. f.* | Vitaceae | Vitaceae | AT | - | + | - | + | + | + |
| 175 | *Cissus populnea Guill.& Perr.* | Vitaceae | Vitaceae | AT | - | + | - | + | + | + |
| 176 | *Cissus producta Afzel.* | Vitaceae | Vitaceae | GC | + | - | - | + | + | + |
| 177 | *Citrullus lanatus (Thunb.) Matsumura &Nakai* | Cucurbitaceae | Cucurbitaceae | AT | - | - | - | + | - | + |
| 178 | *Citrus spp.* | Rutaceae | Rutaceae | I | - | - | - | - | - | + |
| 179 | *Clausena anisata (Willd) hook.f. ex Benth.* | Rutaceae | Rutaceae | AT | - | + | - | + | + | + |
| 180 | *Cleistopholis patens (Benth.) Engl. & Diels* | Annonaceae | Annonaceae | GC | + | + | + | + | + | + |
| 181 | *Clerodendrum capitatum (Wild) Schum.& Thonn.* | Verbenaceae | Verbenaceae | AT | + | + | - | + | + | + |
| 182 | *Clerodendroum polycephalum Bak.* | Verbenaceae | Verbenaceae | GC | - | + | - | - | + | + |
| 183 | *Clerodendrum sassandrense Jongkind* | Verbenaceae | Verbenaceae | GC | - | - | - | - | - | + |
| 184 | *Clerodendrum volubile P. Beauv.* | Verbenaceae | Verbenaceae | AT | + | - | - | + | + | + |
| 185 | *Cnestis ferruginea DC.* | Connaraceae | Connaraceae | GC | + | - | - | + | + | + |
| 186 | *Coccinia barteri (Hook.f.) Keay* | Cucurbitaceae | Cucurbitaceae | GC | - | - | - | + | + | + |
| 187 | *Coccinia grandis (Linn.) J.O.Voigt.* | Cucurbitaceae | Cucurbitaceae | Paleo | - | + | - | + | + | + |
| 188 | *Coffea ebracteolata (Hiern)Brenan* | Rubiaceae | Rubiaceae | GC | + | - | - | - | + | + |
| 189 | *Coffea eketensis Wernham* | Rubiaceae | Rubiaceae | AT | - | - | - | + | - | + |
| 190 | *Coffea spp.* | Rubiaceae | Rubiaceae | I | - | - | - | - | + | + |
| 191 | *Coffea togoensis A.Chev.* | Rubiaceae | Rubiaceae | GCW | - | - | - | + | + | + |
| 192 | *Coix lacryma-jobi Linn.* | Gramineae | Gramineae | Paleo | - | - | - | - | + | + |
| 193 | *Cola gigantea var. glabrescens Brenan & Keay* | Sterculiaceae | Malvaceae | GC | + | + | - | + | + | + |
| 194 | *Cola laurifolia Mast* | Sterculiaceae | Malvaceae | GC | + | + | - | + | - | + |
| 195 | *Cola millenii K. Schum.* | Sterculiaceae | Malvaceae | GCW | - | + | - | + | + | + |
| 196 | *Cola nitida (Vent.) Scott. & Endl.* | Sterculiaceae | Malvaceae | GC | + | - | + | - | + | + |
| 197 | *Cola verticillata (Thonn.) Stapf* | Sterculiaceae | Malvaceae | GC | - | - | - | - | - | + |
| 198 | *Colocasia esculenta (Linn.) Schott* | Araceae | Araceae | Paleo | - | - | - | - | + | + |
| 199 | *Combretum hispidum Laws.* | Combretaceae | Combretaceae | GC | - | - | - | - | - | + |
| 200 | *Combretum molle R.Br.ex G.Don* | Combretaceae | Combretaceae | SZ | - | + | - | + | + | + |
| 201 | *Combretum paniculatum Vent.* | Combretaceae | Combretaceae | AT | + | + | - | + | + | + |
| 202 | *Combretum platypterum (Welw.)Hutch. & Dalz.* | Combretaceae | Combretaceae | GC | + | - | - | - | + | + |
| 203 | *Combretum racemosum P. Beauv.* | Combretaceae | Combretaceae | GC | + | + | - | + | + | + |
| 204 | *Combretum smeathmannii G. Don.* | Combretaceae | Combretaceae | GC | - | + | - | + | - | + |
| 205 | *Commelina africana (C.B.CL.) Brenan* | Commelinaceae | Commelinaceae | GC | - | - | - | - | + | + |
| 206 | *Commelina bengalensis Linn.* | Commelinaceae | Commelinaceae | AT | + | + | - | + | + | + |
| 207 | *Commelina capitata Benth.* | Commelinaceae | Commelinaceae | GC | + | - | - | + | - | + |
| 208 | *Commelina diffusa subsp. Diffusa Burm.f.* | Commelinaceae | Commelinaceae | Pan | - | + | - | + | + | + |
| 209 | *Commelina erecta Linn. subsp. erecta* | Commelinaceae | Commelinaceae | Pan | + | + | - | + | + | + |
| 210 | *Connarus thonningii (CD.) Schellenb.* | Connaraceae | Connaraceae | GC | + | - | - | + | + | + |
| 211 | *Conyza aegyptiaca (Linn.) Ait.var. aegyptiaca.* | Asteraceae | Asteraceae | Paleo | - | - | - | - | - | + |
| 212 | *Cordia alliodora (Ruiz & Pav.) Oken* | Boraginaceae | Boraginaceae | I | - | - | - | - | - | + |
| 213 | *Cordia platythyrsa Baker* | Boraginaceae | Boraginaceae | GC | + | - | + | - | + | + |
| 214 | *Cordia senegalensis Juss.* | Boraginaceae | Boraginaceae | GC | - | - | - | + | + | + |
| 215 | *Costus afer Ker-Gawl.* | Zingiberaceae | Costaceae | GC | + | + | - | + | + | + |
| 216 | *Costus albus A. Chev. ex J. Koechlin* | Zingiberaceae | Costaceae | GC | + | - | - | - | + | + |
| 217 | *Craterosiphon scandens Engel & Gilg.* | Thymelaeaceae | Thymelaeaceae | AT | + | - | - | - | + | + |
| 218 | *Cremaspora triflora (Thonn.) K. Schum.* | Rubiaceae | Rubiaceae | AT | - | + | - | + | + | + |
| 219 | *Crinum jagus (Thomp.) Dandy* | Amaryllidaceae | Amaryllidaceae | AT | + | + | - | + | + | + |
| 220 | *Crinum zeylanicum Auct.= C. ornatum* | Amaryllidaceae | Amaryllidaceae | AT | + | + | - | + | - | + |
| 221 | *Crotonogyne chevalieri (Beille) Keay* | Euphorbiaceae | Euphorbiaceae | GCW | + | - | - | - | - | + |
| 222 | *Crotalaria doniana Baker* | Papilionaceae | Fabaceae | GC | - | - | - | - | + | + |
| 223 | *Croton lobatus Linn.* | Euphorbiaceae | Euphorbiaceae | AT | - | + | - | + | - | + |
| 224 | *Crotalaria pallida Ait.* | Papilionaceae | Fabaceae | Pan | - | + | - | + | - | + |
| 225 | *Ctenitis lanigera (Kuhn) Tard.* | Aspidiaceae | Aspidiaceae | GC | - | - | - | - | + | + |
| 226 | *Cucumis melo var. agrestis Linn. var.agrestis* | Cucurbitaceae | Cucurbitaceae | Pan | - | - | - | + | - | + |
| 227 | *Culcasia angolensis Welw.= C. barombiensis* | Araceae | Araceae | GC | + | - | - | - | + | + |
| 228 | *Culcasia piperoides A. Chev.= C. parviflora* | Araceae | Araceae | GC | + | - | - | - | + | + |
| 229 | *Culcasia saxatilis A. Chev.* | Araceae | Araceae | GC | + | - | - | + | + | + |
| 230 | *Culcasia scandens P. Beauv.* | Araceae | Araceae | GC | - | + | - | + | + | + |
| 231 | *Cussonia kirkii Seemann.* | Araliaceae | Araliaceae | SZ | - | - | - | + | + | + |
| 232 | *Cuviera acutiflora DC.* | Rubiaceae | Rubiaceae | GC | + | - | - | - | + | + |
| 233 | *Cuviera macroura K. Schum.* | Rubiaceae | Rubiaceae | AT | + | - | - | + | + | + |
| 234 | *Cyathula achyrantoides (H. B. & K.) Moq.* | Amaranthaceae | Amaranthaceae | AA | - | + | - | + | + | + |
| 235 | *Cyathea camerouniana Hook.,* | Cyatheaceae | Cyatheaceae | GC | - | - | - | - | - | + |
| 236 | *Cyclosorus afer (Christ) Ching* | Thelypteridaceae | Thelypteridaceae | GC | - | - | - | - | + | + |
| 237 | *Cyclosorus dentatus (Forsk.) ching* | Thelypteridaceae | Thelypteridaceae | GC | - | - | - | - | - | + |
| 238 | *Cyclosorus striatus (Schumach.) Ching.* | Thelypteridaceae | Thelypteridaceae | GC | - | + | - | - | - | + |
| 239 | *Cynanchum longipes N. E. Br.* | Asclepiadaceae | Apocynaceae | GC | - | - | - | - | - | + |
| 240 | *Cynometra megalophylla Harms* | Caesalpiniaceae | Fabaceae | GC | + | + | - | + | - | + |
| 241 | *Cyperus diffusus Vahl* | Cyperaceae | Cyperaceae | GC | - | - | - | - | + | + |
| 242 | *Cyperus halpan Linn.* | Cyperaceae | Cyperaceae | Pan | - | + | - | + | - | + |
| 243 | *Cyperus pectinatus Vahl* | Cyperaceae | Cyperaceae | AT | - | - | - | - | - | + |
| 244 | *Cyphostemma adenopodum (Sprague) Descoings* | Vitaceae | Vitaceae | GC | - | - | - | + | + | + |
| 245 | *Dacryodes klaineana (Pierre) H.J. Lam.* | Burseraceae | Burseraceae | GC | + | - | + | - | + | + |
| 246 | *Dalbergia adami Berh.* | Papilionaceae | Fabaceae | GC | - | - | - | - | + | + |
| 247 | *Dalbergia altissima Bak.f .* | Papilionaceae | Fabaceae | GC | - | - | - | + | + | + |
| 248 | *Dalbergia bignonae Berh.* | Papilionaceae | Fabaceae | GC | - | - | - | - | - | + |
| 249 | *Dalbergia dalzielli Bak. F.* | Papilionaceae | Fabaceae | GCW | - | - | - | - | + | + |
| 250 | *Dalbergia saxatilis Hook.f.* | Papilionaceae | Fabaceae | GCE | - | + | - | + | + | + |
| 251 | *Dalbergiella welwitschii (Bak.)Bak.f.* | Papilionaceae | Fabaceae | GC | + | + | - | - | + | + |
| 252 | *Daniellia thurifera Benn.* | Caesalpiniaceae | Fabaceae | GCW | + | - | - | - | + | + |
| 253 | *Davalia vogelii H.K.* | Davalliaceae | Davalliaceae | GC | - | - | - | - | - | + |
| 254 | *Deinbollia grandifolia Hook.f.* | Sapindaceae | Sapindaceae | GC | + | - | - | + | + | + |
| 255 | *Deinbollia pinnata Schum. & Thonn.* | Sapindaceae | Sapindaceae | GC | - | - | - | + | + | + |
| 256 | *Delonix regia ( Boj.ex Hook.) Raf.* | Caesalpiniaceae | Fabaceae | I | - | + | - | + | - | + |
| 257 | *Dennettia tripetala Bak. f.* | Annonaceae | Annonaceae | GC | + | + | - | + | - | + |
| 258 | *Desmodium adscendens (Sw.) DC* | Papilionaceae | Fabaceae | AA | + | + | - | - | + | + |
| 259 | *Desmodium gangeticum (Linn.) DC.* | Papilionaceae | Fabaceae | Paleo | - | + | - | + | + | + |
| 260 | *Desmodium salicifolium (Poir.) DC.* | Papilionaceae | Fabaceae | AT | - | + | - | + | + | + |
| 261 | *Desmodium triflorum (Linn.)DC.* | Papilionaceae | Fabaceae | Pan | - | + | - | + | + | + |
| 262 | *Desmodium velutinum (Willd.) DC.* | Papilionaceae | Fabaceae | Paleo | - | + | - | + | + | + |
| 263 | *Detarium senegalense J.F.Gmi* | Caesalpiniaceae | Fabaceae | GC | + | + | - | - | + | + |
| 264 | *Dialium guineense Willd.* | Caesalpiniaceae | Fabaceae | GC | + | + | - | + | + | + |
| 265 | *Diaphananthe pellucida (Lindl.) Schltr* | Orchidaceae | Orchidaceae | GCW | - | - | - | - | + | + |
| 266 | *Dichapetalum crassifolium Chodat* | Dichapetalaceae | Dichapetalaceae | GC | + | - | - | - | + | + |
| 267 | *Dichapetalum madagascariense Poir. Var. madagascariense* | Dichapetalaceae | Dichapetalaceae | GC | + | + | - | + | + | + |
| 268 | *Dichapetalum oblongum (Hook.f.ex Benth.) Engel.* | Dichapetalaceae | Dichapetalaceae | GC | - | - | - | + | + | + |
| 269 | *Dicranolepis grandiflora Engel.* | Thymelaeaceae | Thymelaeaceae | GC | - | - | - | - | + | + |
| 270 | *Dicranolepis laciniata Gilg.* | Thymelaeaceae | Thymelaeaceae | GC | - | - | - | - | + | + |
| 271 | *Dictyandra arborescens Welw.* | Rubiaceae | Rubiaceae | GC | - | - | + | - | + | + |
| 272 | *Didymosalpinx abbeokutae Hiern* | Rubiaceae | Rubiaceae | GC | - | - | - | - | + | + |
| 273 | *Digitaria horizontalis Willd.* | Gramineae | Gramineae | Pan | - | + | - | + | - | + |
| 274 | *Dioclea reflexa Hook.f .* | Papilionaceae | Fabaceae | GC | - | + | - | + | + | + |
| 275 | *Diodia scandens Sw.* | Rubiaceae | Rubiaceae | Pan | - | + | - | + | + | + |
| 276 | *Diospyros abyssinica (Hiern)F. White* | Ebenaceae | Ebenaceae | AT | - | - | - | + | + | + |
| 277 | *Dioscorea alata L.* | Dioscoreaceae | Dioscoreaceae | I | - | - | - | - | - | + |
| 278 | *Dioscorea bulbifera L.* | Dioscoreaceae | Dioscoreaceae | Pan | - | + | - | + | + | + |
| 279 | *Dioscorea cayenensis Lam.* | Dioscoreaceae | Dioscoreaceae | GC | - | - | - | + | + | + |
| 280 | *Dioscoreophyllum cumminsii (Stapf) Diels* | Menispermaceae | Menispermaceae | GC | - | - | - | - | + | + |
| 281 | *Dioscorea dumetorum (Kunth) Pax* | Dioscoreaceae | Dioscoreaceae | AT | - | + | - | - | + | + |
| 282 | *Diospyros elliotii (Hiern) F. White* | Ebenaceae | Ebenaceae | GC | - | - | - | - | - | + |
| 283 | *Dioscorea hirtifolia Benth.* | Dioscoreaceae | Dioscoreaceae | GC | - | + | - | - | + | + |
| 284 | *Diospyros liberiensis A. Chev. ex Hutch.* | Ebenaceae | Ebenaceae | GCW | + | - | - | - | + | + |
| 285 | *Dioscorea mangenotiana Miège* | Dioscoreaceae | Dioscoreaceae | GC | - | - | - | + | + | + |
| 286 | *Diospyros mespiliformis Hochst. Ex A. DC.* | Ebenaceae | Ebenaceae | AT | - | + | - | + | + | + |
| 287 | *Dioscorea minutiflora Engel* | Dioscoreaceae | Dioscoreaceae | GC | + | + | - | - | + | + |
| 288 | *Diospyros monbuttensis Gürke* | Ebenaceae | Ebenaceae | GC | - | + | - | + | + | + |
| 289 | *Dioscorea praehensilis Benth.= D. liebrechtsiana* | Dioscoreaceae | Dioscoreaceae | GC | - | + | - | + | + | + |
| 290 | *Dioscorea preussii Pax.* | Dioscoreaceae | Dioscoreaceae | GC | - | + | - | + | + | + |
| 291 | *Dioscorea sagitifolia Pax= D . Lecardii* | Dioscoreaceae | Dioscoreaceae | GC | + | + | - | - | + | + |
| 292 | *Dioscorea sansibarensis Pax* | Dioscoreaceae | Dioscoreaceae | GC | + | + | - | - | + | + |
| 293 | *Dioscorea smilacifolia De Wild.* | Dioscoreaceae | Dioscoreaceae | GC | + | - | - | - | + | + |
| 294 | *Dioscorea togoensis Kunth.* | Dioscoreaceae | Dioscoreaceae | AT | - | + | - | + | + | + |
| 295 | *Dioscoreophyllum volkensii Engl.* | Menispermaceae | Menispermaceae | GC | - | - | - | - | - | + |
| 296 | *Discoglypremna caloneura (Pax) Prain.* | Euphorbiaceae | Euphorbiaceae | GC | + | - | + | - | + | + |
| 297 | *Dissotis multiflora (Sm.) Tri* | Melastomataceae | Melastomataceae | GC | - | - | - | - | - | + |
| 298 | *Distemonanthus benthamianus Baill.* | Caesalpiniaceae | Fabaceae | GC | + | - | - | - | + | + |
| 299 | *Dombeya buettneri K.Schum.* | Sterculiaceae | Malvaceae | AT | - | - | - | - | + | + |
| 300 | *Dombeya elliotii* | Sterculiaceae | Malvaceae | GC | - | - | - | - | + | + |
| 301 | *Doryopteris kirkii (Hook.) Alston* | Adiantaceae | Adiantaceae | GC | - | - | - | - | + | + |
| 302 | *Dovyalis zenkeri Gilg. I.c* | Flacourtiaceae | Salicaceae | GC | - | - | - | - | - | + |
| 303 | *Dracaena arborea Link.* | Agavaceae | Asparagaceae | GC | + | + | + | + | + | + |
| 304 | *Dracaena camerooniana Bak.* | Agavaceae | Asparagaceae | AT | + | - | - | - | + | + |
| 305 | *Dracaena mannii Bak.* | Agavaceae | Asparagaceae | GC | + | - | + | - | + | + |
| 306 | *Dracaena ovata Ker-Gawl.* | Agavaceae | Asparagaceae | GC | + | - | - | - | - | + |
| 307 | *Dracaena surculosa Lindl.var. surculosa* | Agavaceae | Asparagaceae | GC | + | - | - | + | + | + |
| 308 | *Drypetes aframensis Hutch.* | Euphorbiaceae | Putranjivaceae | GC | + | - | - | - | + | + |
| 309 | *Drypetes afzeli Hutch.* | Euphorbiaceae | Putranjivaceae | GCW | + | - | - | - | - | + |
| 310 | *Drypetes aylmeri Hutct .& Dalz.* | Euphorbiaceae | Putranjivaceae | GCW | - | - | - | - | - | + |
| 311 | *Drypetes floribunda (Müll. Arg.) Hutch.* | Euphorbiaceae | Putranjivaceae | AT | - | + | + | + | - | + |
| 312 | *Drypetes gilgiana(Pax)(Pax)&K.Hoffm.* | Euphorbiaceae | Putranjivaceae | GC | + | - | - | - | - | + |
| 313 | *Drypetes klainei Pierre ex Pax* | Euphorbiaceae | Putranjivaceae | GC | + | - | - | - | - | + |
| 314 | *Drypetes leonensis Pax* | Euphorbiaceae | Putranjivaceae | GCW | + | - | - | - | + | + |
| 315 | *Drypetes parviflora Müll. Arg.Pax. & K. Hoffm.* | Euphorbiaceae | Putranjivaceae | GC | + | - | - | + | - | + |
| 316 | *Dyschoriste perrottetii ( Nees) O. Kuntze* | Acanthaceae | Acanthaceae | GC | - | + | - | - | - | + |
| 317 | *Elaeophorbia drupifera ( Thonn.) Stapf.* | Euphorbiaceae | Euphorbiaceae | AT | - | - | + | + | - | + |
| 318 | *Elaeophorbia grandifolia (Haw.) Croizat* | Euphorbiaceae | Euphorbiaceae | AT | + | - | - | - | + | + |
| 319 | *Elaeis guineensis Jacq.* | Arecaceae | Arecaceae | GC | + | - | - | + | + | + |
| 320 | *Elytraria marginata Vahl* | Acanthaceae | Acanthaceae | GC | + | + | - | + | + | + |
| 321 | *Embelia guineensis Baker* | Myrsinaceae | Primulaceae | GC | - | - | - | - | + | + |
| 322 | *Emilia coccinea (Sims) G. Don* | Asteraceae | Asteraceae | GC | - | - | - | - | - | + |
| 323 | *Entandrophragma angolense (Welw.) C.DC.* | Meliaceae | Meliaceae | GC | + | - | + | - | + | + |
| 324 | *Entandrophragma cylindricum (sprague) Sprague* | Meliaceae | Meliaceae | GC | + | - | + | - | + | + |
| 325 | *Entada mannii (Oliv.) Tisserant* | Mimosaceae | Fabaceae | AT | - | + | - | + | + | + |
| 326 | *Entada pursaetha DC.* | Mimosaceae | Fabaceae | GC | - | - | - | - | + | + |
| 327 | *Eremomastax speciosa (Hochst.) Cufod.* | Acanthaceae | Acanthaceae | GC | - | + | - | - | + | + |
| 328 | *Eriocoelum kerstingii Gilg. & Engl.* | Sapindaceae | Sapindaceae | AT | - | + | - | - | + | + |
| 329 | *Erythrococca africana (Baill.) Prain* | Euphorbiaceae | Euphorbiaceae | GC | - | - | - | - | + | + |
| 330 | *Erythrococca anomala (Juss.ex Poir.)Prain.* | Euphorbiaceae | Euphorbiaceae | GC | + | - | - | + | + | + |
| 331 | *Erythroxylum emarginatum Thonn.* | Erythroxylaceae | Erythroxylaceae | AT | - | + | + | + | - | + |
| 332 | *Erythroxylum mannii Oliv.* | Erythroxylaceae | Erythroxylaceae | GC | + | - | - | - | + | + |
| 333 | *Erythrina mildbraedii Harms* | Papilionaceae | Fabaceae | GC | + | - | - | - | + | + |
| 334 | *Erythrophleum suaveolens (Guill. & Pherr.) Brenan.* | Caesalpiniaceae | Fabaceae | AT | - | + | + | + | + | + |
| 335 | *Erythrina vogelii Hook. f.* | Papilionaceae | Fabaceae | GCW | + | - | - | - | - | + |
| 336 | *Euadenia trifoliolata (Schum. & Thonn.) Oliv.* | Capparaceae | Capparaceae | GC | + | + | - | + | + | + |
| 337 | *Euclinia longiflora Salisb.* | Rubiaceae | Rubiaceae | GC | - | - | - | - | + | + |
| 338 | *Eugenia calophylloides DC* | Myrtaceae | Myrtaceae | GCW | - | - | - | - | - | + |
| 339 | *Eugenia leonensis Engl. & v. Brehm.* | Myrtaceae | Myrtaceae | GCW | - | - | - | - | + | + |
| 340 | *Eugenia malaccensis Linn.* | Myrtaceae | Myrtaceae | I | - | - | - | - | + | + |
| 341 | *Eugenia salacioides Laws. ex Hutch = Eugenia leonensis* | Myrtaceae | Myrtaceae | GCW | - | - | - | - | - | + |
| 342 | *Eulophia guineensis Lindl* | Orchidaceae | Orchidaceae | AT | + | + | - | - | + | + |
| 343 | *Eulophia maculata (Lindley.) Reichenb.* | Orchidaceae | Orchidaceae | GC | + | - | - | + | + | + |
| 344 | *Eupatorium microstemon Cass.* | Asteraceae | Asteraceae | I | - | + | - | - | + | + |
| 345 | *Eupatorium odoratum Linn.* | Asteraceae | Asteraceae | Pan | + | - | - | - | + | + |
| 346 | *Fagara leprieurii (Guill.& perr.) Engl.* | Rutaceae | Rutaceae | GC | - | - | - | + | + | + |
| 347 | *Fagara macrophylla Engl. = Zanthoxylum macrophylla* | Rutaceae | Rutaceae | GC | + | - | + | - | + | + |
| 348 | *Fagara viridis A. Chev.* | Rutaceae | Rutaceae | GC | - | - | - | - | - | + |
| 349 | *Ficus asperifolia Miq.* | Moraceae | Moraceae | AT | - | + | - | + | - | + |
| 350 | *Ficus barteri Sprague* | Moraceae | Moraceae | GC | - | - | - | - | + | + |
| 351 | *Ficus congensis Engel. = F. trichopoda* | Moraceae | Moraceae | AM | - | + | - | + | + | + |
| 352 | *Ficus craterostoma Mildbr.* | Moraceae | Moraceae | GC | + | - | - | - | + | + |
| 353 | *Ficus dicranostyla Mildbr.* | Moraceae | Moraceae | AT | - | + | - | + | + | + |
| 354 | *Ficus elegans (Miq.) Miq.* | Moraceae | Moraceae | GC | - | - | - | + | + | + |
| 355 | *Ficus exasperata Vahl.* | Moraceae | Moraceae | AT | - | + | - | + | + | + |
| 356 | *Ficus lutea Vahl.* | Moraceae | Moraceae | GC | + | - | - | + | + | + |
| 357 | *Ficus lyrata Warb.* | Moraceae | Moraceae | GC | + | + | - | + | + | + |
| 358 | *Ficus macrosperma Mildbr = F. sansibarica* | Moraceae | Moraceae | GC | + | - | - | - | - | + |
| 359 | *Ficus mucuso Ficalho* | Moraceae | Moraceae | GC | + | - | + | + | + | + |
| 360 | *Ficus natalensis Hochtst.* | Moraceae | Moraceae | AT | + | - | - | + | + | + |
| 361 | *Ficus ovata Vahl.* | Moraceae | Moraceae | GC | - | + | - | + | + | + |
| 362 | *Ficus polita Vahl.* | Moraceae | Moraceae | AT | - | + | - | + | + | + |
| 363 | *Ficus populifolia Vahl* | Moraceae | Moraceae | AT | - | - | - | - | + | + |
| 364 | *Ficus sagittifolia Warb.ex Mildbr.* | Moraceae | Moraceae | GC | + | - | - | - | + | + |
| 365 | *Ficus saussureana DC.* | Moraceae | Moraceae | GC | - | - | - | - | + | + |
| 366 | *Ficus scott-elliotii Mildbr.& Burrett* | Moraceae | Moraceae | GC | - | - | - | - | - | + |
| 367 | *Ficus sur Forssk= F. capencis* | Moraceae | Moraceae | AT | - | - | + | - | + | + |
| 368 | *Ficus tesselata Warb* | Moraceae | Moraceae | GC | + | + | - | - | + | + |
| 369 | *Ficus thonningii Blume* | Moraceae | Moraceae | AT | + | + | + | + | + | + |
| 370 | *Ficus umbellata Vahl.* | Moraceae | Moraceae | AT | + | - | - | + | + | + |
| 371 | *Ficus vallis-choudae Delile* | Moraceae | Moraceae | AT | - | - | - | + | + | + |
| 372 | *Ficus varifolia Delilie* | Moraceae | Moraceae | GC | - | - | - | - | + | + |
| 373 | *Ficus vogeliana (Miq.) Miq.* | Moraceae | Moraceae | GC | + | + | + | - | + | + |
| 374 | *Flabellaria paniculata Cav.* | Malpighiaceae | Malpighiaceae | GC | + | + | - | + | + | + |
| 375 | *Flacourtia vogelii Hook. f.* | Flacourtiaceae | Salicaceae | GC | - | - | - | - | - | + |
| 376 | *Flocospa africana (P. Beauv.) C. B. Cl.* | Commelinaceae | Commelinaceae | GC | + | + | - | - | + | + |
| 377 | *Funtumia africana (Benth.) Stapf* | Apocynaceae | Apocynaceae | GC | + | + | + | - | + | + |
| 378 | *Gaertnera paniculata Benth.* | Rubiaceae | Rubiaceae | GC | - | + | - | - | + | + |
| 379 | *Garcinia acuminata A.Chev.= G. ovalifolia* | Clusiaceae | Clusiaceae | AT | + | + | - | - | + | + |
| 380 | *Garcinia afzelii Engl.* | Clusiaceae | Clusiaceae | AT | + | - | - | - | + | + |
| 381 | *Garcinia kola Heckel.* | Clusiaceae | Clusiaceae | GC | + | - | + | - | - | + |
| 382 | *Garcinia livingtonei T. Anders* | Clusiaceae | Clusiaceae | SZ | - | + | - | - | - | + |
| 383 | *Garcinia polyantha Oliv. =G. smeathmanii* | Clusiaceae | Clusiaceae | GC | - | - | + | - | - | + |
| 384 | *Garcinia punctata Oliv.* | Clusiaceae | Clusiaceae | GCE | - | - | + | - | - | + |
| 385 | *Gardenia nitida Hook.* | Rubiaceae | Rubiaceae | GC | + | - | - | + | - | + |
| 386 | *Geophila obvallata (Schum.)F.Didr* | Rubiaceae | Rubiaceae | GC | + | + | - | - | - | + |
| 387 | *Geophila rupens Linn.* | Rubiaceae | Rubiaceae | GC | + | - | - | - | - | + |
| 388 | *Gerrardanthus zenkeri Harms & Gilg* | Cucurbitaceae | Cucurbitaceae | GC | - | - | - | - | - | + |
| 389 | *Gliricidia sepium (Jacq.) Walp* | Papilionaceae | Fabaceae | I | - | - | - | - | - | + |
| 390 | *Gloriosa simplex Linn. = G. superba* | Liliaceae | Colchiacaceae | GC | + | + | - | - | + | + |
| 391 | *Glyphaea brevis (Spreng.) Monachino* | Tiliaceae | Malvaceae | GC | + | + | + | + | + | + |
| 392 | *Gmelina arborea Roxb.* | Verbenaceae | Verbenaceae | I | - | + | - | - | - | + |
| 393 | *Gongronema angolense (N.E.Br.) Bullock.* | Asclepiadaceae | Apocynaceae | GC | - | - | - | + | + | + |
| 394 | *Gongronema latifolium Benth.* | Asclepiadaceae | Apocynaceae | GC | - | + | - | + | + | + |
| 395 | *Gouania longipetala Hermsl.* | Rhamnaceae | Rhamnaceae | GC | + | - | - | + | + | + |
| 396 | *Grewia barteri Burret.* | Tiliaceae | Malvaceae | SZ | - | - | - | - | + | + |
| 397 | *Grewia carpinifolia Juss.* | Tiliaceae | Malvaceae | GC | + | - | - | + | + | + |
| 398 | *Grewia flavescens Juss.* | Tiliaceae | Malvaceae | GC | - | + | - | - | - | + |
| 399 | *Grewia malacocarpa Mast.* | Tiliaceae | Malvaceae | GC | + | - | - | + | + | + |
| 400 | *Grewia mollis Juss.* | Tiliaceae | Malvaceae | AT | + | + | - | - | + | + |
| 401 | *Grewia pubescens P. Beav.* | Tiliaceae | Malvaceae | GC | - | - | - | + | + | + |
| 402 | *Grewia villosa Willd.* | Tiliaceae | Malvaceae | AA | + | - | - | + | - | + |
| 403 | *Griffonia simplicifolia (Vahl. ex DC.) Baill.* | Caesalpiniaceae | Fabaceae | GC | + | - | - | + | + | + |
| 404 | *Guarea cedrata (A.Chev.)Pellegr.* | Meliaceae | Meliaceae | GC | + | - | + | - | + | + |
| 405 | *Gymnostemon zaizou Aubrév. et Pellegr.* | Simaroubaceae | Simaroubaceae | GCW | + | - | - | - | + | + |
| 406 | *Habenaria buettneriana Kraenz* | Orchidaceae | Orchidaceae | AT | - | - | - | - | + | + |
| 407 | *Hannoa klaineana Pierre et Engl.= Quassia klaineana* | Simaroubaceae | Simaroubaceae | GC | + | - | - | - | - | + |
| 408 | *Harrisonia abyssinica Oliv.* | Simaroubaceae | Simaroubaceae | AT | - | - | - | + | + | + |
| 409 | *Harungana madagascariensis Lam. ex Poir.* | Clusiaceae | Hypericaceae | AM | - | - | + | - | + | + |
| 410 | *Heisteria parvifolia Sim.* | Olacaceae | Olacaceae | GC | + | - | + | - | + | + |
| 411 | *Heterotis rotundifolia (Sm.) Jac.-Fel.* | Melastomataceae | Melastomataceae | GC | + | - | - | + | + | + |
| 412 | *Hevea brasiliensis (Kunth)Mull.arg.* | Euphorbiaceae | Euphorbiaceae | I | - | - | - | - | - | + |
| 413 | *Hexalobus crispiflorus A. Rich.* | Annonaceae | Annonaceae | AT | - | + | + | - | - | + |
| 414 | *Hibiscus asper Hook. f.* | Malvaceae | Malvaceae | AT | + | + | - | + | - | + |
| 415 | *Hibiscus lunarifolius Willd.* | Malvaceae | Malvaceae | AT | - | + | - | + | + | + |
| 416 | *Hibiscus rostellatus Guill. & Perr.* | Malvaceae | Malvaceae | GC | + | - | - | + | + | + |
| 417 | *Hibiscus surattensis Linn.* | Malvaceae | Malvaceae | Paleo | - | + | - | + | - | + |
| 418 | *Hildegardia barteri (Mast.) Kosterm.* | Sterculiaceae | Malvaceae | AT | - | - | - | + | + | + |
| 419 | *Hilleria latifolia (Lam.) H.Walt.* | Phytolaccaceae | Phytolaccaceae | Pan | - | - | - | + | + | + |
| 420 | *Hippocratea africana (Willd.) Loes. ex Engl.* | Celastraceae | Celastraceae | GC | - | - | - | + | + | + |
| 421 | *Hippocratea apocynoides Welw.ex Oliv.* | Celastraceae | Celastraceae | GC | - | - | - | + | + | + |
| 422 | *Hippocratea indica Willd.* | Celastraceae | Celastraceae | GC | - | - | - | + | + | + |
| 423 | *Hippocratea pallens Planch. ex Oliv.= Apodostigma pallens* | Celastraceae | Celastraceae | AT | + | - | - | + | + | + |
| 424 | *Hippocratea welwitchii (Oliv.)N.Hallé* | Celastraceae | Celastraceae | GC | - | + | - | + | - | + |
| 425 | *Holarrhena floribunda (G. DON.) Dur. & Schinz.* | Apocynaceae | Apocynaceae | AT | + | + | + | + | + | + |
| 426 | *Holoptelea grandis (Hutch.) MildBr.* | Ulmaceae | Ulmaceae | GC | + | - | + | + | + | + |
| 427 | *Homalium letestui Pellegr* | Flacourtiaceae | Salicaceae | GC | + | - | + | - | + | + |
| 428 | *Hoslundia opposita Vahl.* | Labiatae | Lamiaceae | AM | - | + | - | + | + | + |
| 429 | *Hugonia obtusifolia C.H.* | Linaceae | Linaceae | GCE | - | - | - | - | - | + |
| 430 | *Hugonia planchonii Hook.f.* | Linaceae | Linaceae | GC | + | - | - | + | + | + |
| 431 | *Hugonia platysepala Welw.* | Linaceae | Linaceae | GC | + | - | - | - | + | + |
| 432 | *Hunteria ghanensis J.B.Hall & Leeuwenberg* | Apocynaceae | Apocynaceae | GCW | - | - | - | - | + | + |
| 433 | *Hura crepitans Linn.* | Euphorbiaceae | Euphorbiaceae | I | - | - | - | - | - | + |
| 434 | *Hymenostegia afzelii (Oliver) Harms* | Caesalpiniaceae | Fabaceae | GC | + | - | - | + | + | + |
| 435 | *Hymenocardia lyrata Tul* | Euphorbiaceae | Phyllantaceae | GCW | + | - | - | - | + | + |
| 436 | *Hypselodelphys poggeana (K. Schum.) Milne-Redh.* | Marantaceae | Marantaceae | GC | - | + | - | + | + | + |
| 437 | *Hypselodelphys violacea (Ridl.) Milne-Redh.* | Marantaceae | Marantaceae | GC | + | + | - | + | + | + |
| 438 | *Illigera pentaphylla Welw* | Hernandiaceae | Hernandiaceae | GC | - | - | - | - | - | + |
| 439 | *Indigofera heudolotii Benth. ex Bak* | Papilionaceae | Fabaceae | AT | - | - | - | - | - | + |
| 440 | *Indigofera macrophylla Schum. & Thonn.* | Papilionaceae | Fabaceae | GC | - | + | - | + | + | + |
| 441 | *Ipomoea alba Linn.* | Convolvulaceae | Convolvulaceae | Pan | - | + | - | + | + | + |
| 442 | *Ipomoea involucrata P. Beauv.* | Convolvulaceae | Convolvulaceae | AT | + | + | - | + | + | + |
| 443 | *Ipomoea mauritiana Jacq.* | Convolvulaceae | Convolvulaceae | Pan | + | + | - | + | + | + |
| 444 | *Ipomoea pileata Roxb.* | Convolvulaceae | Convolvulaceae | AT | - | - | - | + | - | + |
| 445 | *Irvingia gabonensis Aubry-Lecomte ex O'Rorke) Baill.* | Irvingiaceae | Irvingiaceae | GC | + | + | + | + | + | + |
| 446 | *Isachne buettneri Hack.* | Gramineae | Gramineae | GC | + | - | - | + | + | + |
| 447 | *Isolona cooperi Hutch. & Dalziel ex Coop. & Record* | Annonaceae | Annonaceae | GCW | + | - | - | - | - | + |
| 448 | *Ixora brachypoda DC.* | Rubiaceae | Rubiaceae | AT | - | + | - | - | - | + |
| 449 | *Jasminum dichotomum Vahl.* | Oleaceae | Oleaceae | GC | + | + | - | + | - | + |
| 450 | *Jasminum pauciflorum Benth.* | Oleaceae | Oleaceae | GC | + | + | - | + | + | + |
| 451 | *Jaundea pinnata (P. Beauv.) Schellenb.=Rourea thomsonii* | Connaraceae | Connaraceae | GC | + | + | - | + | + | + |
| 452 | *Justicia flava (Forsk.) Vahl* | Acanthaceae | Acanthaceae | GC | - | - | - | + | - | + |
| 453 | *Kalanchoe crenata (andr.) Ham.* | Crassulaceae | Crassulaceae | AT | - | - | - | + | - | + |
| 454 | *Khaya anthotheca (Welw.) C.DC.* | Meliaceae | Meliaceae | GC | - | - | + | - | + | + |
| 455 | *Khaya grandifoliola DC.* | Meliaceae | Meliaceae | GC | - | + | - | - | + | + |
| 456 | *Khaya senegalensis Desr.)A.Juss* | Meliaceae | Meliaceae | AT | - | + | - | - | - | + |
| 457 | *Kigelia africana (Lam.) Benth.* | Bignoniaceae | Bignoniaceae | AT | + | + | + | + | - | + |
| 458 | *Klainedoxa gabonensis Pierre* | Irvingiaceae | Irvingiaceae | GC | + | - | + | - | + | + |
| 459 | *Kolobopetalum leonense Hutch.& Dalz.* | Menispermaceae | Menispermaceae | GC | - | - | - | - | - | + |
| 460 | *Kolobopetalum ovatum Stapf* | Menispermaceae | Menispermaceae | GC | - | - | - | - | - | + |
| 461 | *Lagenaria breviflora Benth.* | Cucurbitaceae | Cucurbitaceae | GC | - | - | - | + | + | + |
| 462 | *Lagenaria guinensis ( Don)Jeff.* | Cucurbitaceae | Cucurbitaceae | GC | - | - | - | - | + | + |
| 463 | *Landolphia dulcis (R.Br. ex Sabine) Pichon.* | Apocynaceae | Apocynaceae | AT | + | - | - | + | + | + |
| 464 | *Landolphia hirsuta (Hua) Pichon.* | Apocynaceae | Apocynaceae | AT | + | - | - | - | + | + |
| 465 | *Landolphia owariensis P. Beauv.* | Apocynaceae | Apocynaceae | GC | + | + | - | + | + | + |
| 466 | *Landolphia togolana (Hall.f.)Pichon.* | Apocynaceae | Apocynaceae | GC | - | + | - | + | + | + |
| 467 | *Alafia multiflora (Stapf) Stapf* | Apocynaceae | Apocynaceae | GC | - | - | - | - | + | + |
| 468 | *Lannea nigritana (Sc. Elliot) Keay var.nigritana* | Anacardiaceae | Anacardiaceae | AT | + | + | - | + | + | + |
| 469 | *Lannea welwitchii (Hiern) Engl.* | Anacardiaceae | Anacardiaceae | GC | + | - | + | + | - | + |
| 470 | *Laportea ovalifolia (Schum & Thonn.) Chew* | Urticaceae | Urticaceae | GC | - | - | - | + | + | + |
| 471 | *Lasiodiscus mildbraedii Engl.= L. mannii* | Rhamnaceae | Rhamnaceae | GC | + | - | - | + | - | + |
| 472 | *Lecaniodiscus cupanioides Planch. & Benth.* | Sapindaceae | Sapindaceae | GC | - | + | + | + | + | + |
| 473 | *Leea guineensis G. Don.* | Leeaceae | Leeaceae | AM | + | + | - | + | + | + |
| 474 | *Lepistemon owariense (P.Beauv.)Hallier f.* | Convolvulaceae | Convolvulaceae | AT | - | + | - | + | + | + |
| 475 | *Leptoderris brachyptera (Benth.) Dunn.* | Papilionaceae | Fabaceae | GC | - | + | - | - | + | + |
| 476 | *Leptaspis cochleata Thwaites = L. zeylanica* | Gramineae | Gramineae | Paleo | + | - | - | - | - | + |
| 477 | *Leptaulus daphnoides Benth.* | Icacinaceae | Cardiopteridaceae | GC | + | - | - | + | - | + |
| 478 | *Leptoderris fasciculata (Benth.) Dunn.* | Papilionaceae | Fabaceae | GC | + | - | - | + | + | + |
| 479 | *Lindackeria dentata (Oliv.) Gilg* | Flacourtiaceae | Achariaceae | GC | - | + | - | - | + | + |
| 480 | *Linociera nilotica Oliv.= Chionanthus niloticus* | Oleaceae | Oleaceae | AT | - | + | - | - | + | + |
| 481 | *Lonchitis currori (H.K.) Mett.ex Kühn* | Dennstaedtiaceae | Dennstaedtiaceae | GC | - | - | - | - | + | + |
| 482 | *Lonchocarpus cyanescens (Schum. & Thonn.) Benth.* | Papilionaceae | Fabaceae | AT | - | + | - | + | + | + |
| 483 | *Lonchocarpus sericeus (Poir.)Kunth* | Papilionaceae | Fabaceae | AA | - | + | - | + | + | + |
| 484 | *Lovoa trichilioides Harms* | Meliaceae | Meliaceae | GC | + | - | + | - | + | + |
| 485 | *Loxogramme lanceolata (Sw.) Presl.* | Polypodiaceae | Polypodiaceae | GC | - | - | - | - | + | + |
| 486 | *Ludwigia abyssinica A. Rich.* | Onagraceae | Onagraceae | AM | - | - | - | - | - | + |
| 487 | *Ludwigia erecta (Linn.) Hara.* | Onagraceae | Onagraceae | AM | - | - | - | + | - | + |
| 488 | *Ludwigia octovalvis (Jacq) Raven* | Onagraceae | Onagraceae | AM | - | - | - | + | - | + |
| 489 | *Ludwigia stenorraphe (Brenan ) Hara subsp. stenorraphe* | Onagraceae | Onagraceae | AT | - | - | - | + | - | + |
| 490 | *Luffa cylindrica (Linn.) M.J.Roem.* | Cucurbitaceae | Cucurbitaceae | I | - | + | - | + | - | + |
| 491 | *Lygodium smithianum Pl.* | Schizaeaceae | Lygodiaceae | GC | + | - | - | - | - | + |
| 492 | *Macaranga barteri Müll. Arg.* | Euphorbiaceae | Euphorbiaceae | GC | + | - | + | - | + | + |
| 493 | *Macaranga heterophylla (Mll. Arg.) Müll. Arg.* | Euphorbiaceae | Euphorbiaceae | GC | - | - | - | - | + | + |
| 494 | *Macaranga heudelotii Baill.* | Euphorbiaceae | Euphorbiaceae | GC | - | - | - | - | + | + |
| 495 | *Macaranga hurifolia Beille* | Euphorbiaceae | Euphorbiaceae | GC | + | - | - | - | - | + |
| 496 | *Macaranga spinosa Müll. Arg.* | Euphorbiaceae | Euphorbiaceae | GC | - | - | + | - | + | + |
| 497 | *Macrosphyra longistyla (DC.) Hiern.* | Rubiaceae | Rubiaceae | GC | - | + | - | + | + | + |
| 498 | *Maesopsis eminii Engl.* | Rhamnaceae | Rhamnaceae | GC | + | - | + | - | + | + |
| 499 | *Malacantha alnifolia (Bak.) Pierre = Pouteria alnifolia* | Sapotaceae | Sapotaceae | AT | - | + | - | + | + | + |
| 500 | *Mallotus oppositifolius (Geisel.) Müll.* | Euphorbiaceae | Euphorbiaceae | AM | + | + | + | + | + | + |
| 501 | *Mammea africana Sabine* | Clusiaceae | Clusiaceae | GC | + | - | + | - | + | + |
| 502 | *Mangifera indica Linn.* | Anacardiaceae | Anacardiaceae | I | - | + | - | + | + | + |
| 503 | *Manihot esculenta Crantz* | Euphorbiaceae | Euphorbiaceae | I | - | + | - | + | + | + |
| 504 | *Manilkara multinervis (Baker) Dubard. = M. obovata* | Sapotaceae | Sapotaceae | AT | + | + | - | - | + | + |
| 505 | *Mansonia altissima (A. Chev.)A.Chev.var.altissima* | Sterculiaceae | Malvaceae | GC | + | - | - | + | + | + |
| 506 | *Marattia fraxinea J.Smith.* | Marattiaceae | Marattiaceae | Pan | - | - | - | - | - | + |
| 507 | *Marantochloa holostachya (Bak)* | Marantaceae | Marantaceae | GCE | - | - | - | - | - | + |
| 508 | *Marantochloa leucantha (K.Schum) Milne-Redhead* | Marantaceae | Marantaceae | GC | + | - | - | - | - | + |
| 509 | *Marantochloa purpurea (Ridl.) Milne-Redh.* | Marantaceae | Marantaceae | GC | + | + | - | + | - | + |
| 510 | *Mareya micrantha (Benth.) Muell.* | Euphorbiaceae | Euphorbiaceae | GC | + | - | - | - | + | + |
| 511 | *Margaritaria discoidea (Baiil) Webster* | Euphorbiaceae | Phyllantaceae | AT | + | + | + | + | + | + |
| 512 | *Markhamia lutea (Benth.) K. Schum.* | Bignoniaceae | Bignoniaceae | GC | - | - | - | - | - | + |
| 513 | *Markhamia tomentosa (Benth.) K.Schum.* | Bignoniaceae | Bignoniaceae | GC | - | + | + | + | + | + |
| 514 | *Massularia acuminata (G.Don) Bullock ex hoyle* | Rubiaceae | Rubiaceae | GC | + | - | + | - | + | + |
| 515 | *Megaphrynium macrostachyum (Benth.) Milne-Redhead* | Marantaceae | Marantaceae | GC | + | + | - | - | + | + |
| 516 | *Melastomastrum capitatum Vahl* | Melastomataceae | Melastomataceae | AT | - | - | - | - | + | + |
| 517 | *Melanthera elliptica O. Hoffm.* | Asteraceae | Asteraceae | GC | - | - | - | - | - | + |
| 518 | *Melanthera scandens (Schum. & Thonn.)Roberty* | Asteraceae | Asteraceae | GC | - | + | - | + | + | + |
| 519 | *Memecylon afzelii var. afzelii G. Don.* | Melastomataceae | Melastomataceae | GC | + | + | - | + | + | + |
| 520 | *Memecylon cinnamomoides G. Don Gen.= Warneckea cinnamomoides* | Melastomataceae | Melastomataceae | GC | + | - | - | - | + | + |
| 521 | *Merremia tridentata subsp angustifolia (Jacq.) Ooststr* | Convolvulaceae | Convolvulaceae | AT | - | - | - | + | - | + |
| 522 | *Mezoneuron benthamianum Baill.* | Caesalpiniaceae | Fabaceae | GC | - | + | - | + | + | + |
| 523 | *Microglossa afzelii O. Offm.var. afzelii* | Asteraceae | Asteraceae | GC | + | - | - | + | - | + |
| 524 | *Microgramma ovariensis (Desv.) Alston* | Polypodiaceae | Polypodiaceae | GC | + | - | - | - | - | + |
| 525 | *Microdesmis puberula Hook. f. Planch.= M. keayana* | Pandaceae | Pandaceae | GC | + | + | + | + | + | + |
| 526 | *Microsorium punctatum (Linn.) Kopel* | Polypodiaceae | Polypodiaceae | Paleo | + | - | - | - | + | + |
| 527 | *Microlepia speluncae (Linn.) Moore* | Dennstaedtiaceae | Dennstaedtiaceae | Paleo | - | - | - | - | + | + |
| 528 | *Mikania chenopodiifolia Willd.* | Asteraceae | Asteraceae | GC | - | - | - | + | + | + |
| 529 | *Mikania cordata (Burm.f.) B.L.Robinson var. cordata* | Asteraceae | Asteraceae | GC | + | + | - | - | + | + |
| 530 | *Mikaniopsis maitlandii C.D Adams* | Asteraceae | Asteraceae | GCW | - | - | - | - | - | + |
| 531 | *Milicia excelsa (Welw.) C.C. Berg.* | Moraceae | Moraceae | GC | + | + | + | + | + | + |
| 532 | *Millettia chrysophylla Dunn* | Papilionaceae | Fabaceae | GC | + | - | - | - | + | + |
| 533 | *Millettia thonningii (Schum. & Thonn. ) Bak.* | Papilionaceae | Fabaceae | DG | - | + | - | + | + | + |
| 534 | *Millettia warneckei var. warneckei Harms var. warneckei* | Papilionaceae | Fabaceae | GC | - | + | - | + | - | + |
| 535 | *Millettia zechiana Harms* | Papilionaceae | Fabaceae | GC | + | - | - | + | + | + |
| 536 | *Mimosa invisa Mart. ex Colla* | Mimosaceae | Fabaceae | I | - | - | - | + | + | + |
| 537 | *Mimusops kummel Hochst.* | Sapotaceae | Sapotaceae | AT | - | + | - | - | + | + |
| 538 | *Mitragyna stipulosa O. Kuntze* | Rubiaceae | Rubiaceae | AT | + | - | - | - | + | + |
| 539 | *Momordica angustisepala Harms* | Cucurbitaceae | Cucurbitaceae | GC | - | - | - | + | + | + |
| 540 | *Momordica charantia Linn.* | Cucurbitaceae | Cucurbitaceae | Paleo | + | + | - | + | + | + |
| 541 | *Momordica cissoides Linn.* | Cucurbitaceae | Cucurbitaceae | GC | + | + | - | + | + | + |
| 542 | *Momordica foetida Schum.* | Cucurbitaceae | Cucurbitaceae | AT | - | - | - | - | + | + |
| 543 | *Momordica multiflora Hook.f.* | Cucurbitaceae | Cucurbitaceae | GC | - | - | - | - | - | + |
| 544 | *Monanthotaxis parvifolia (Oliv.) Verdc.* | Annonaceae | Annonaceae | AT | + | + | - | + | - | + |
| 545 | *Monantotaxis whytei (Stapf) Verdc.* | Annonaceae | Annonaceae | GC | + | + | - | - | - | + |
| 546 | *Mondia withei (Hook. F.) Skeels* | Asclepiadaceae | Apocynaceae | AT | - | - | - | - | + | + |
| 547 | *Monechma ciliatum (Jacq.) Milne-Redhead* | Acanthaceae | Acanthaceae | AT | - | + | - | + | + | + |
| 548 | *Monodora myristica (Gaertn.) Dunal* | Annonaceae | Annonaceae | GC | + | - | - | - | + | + |
| 549 | *Monodora tenuifolia Benth.* | Annonaceae | Annonaceae | GC | + | + | - | + | + | + |
| 550 | *Morelia senegalensis A.Rich.ex DC* | Rubiaceae | Rubiaceae | AT | + | + | - | + | - | + |
| 551 | *Morinda lucida Benth.* | Rubiaceae | Rubiaceae | AT | + | + | + | + | + | + |
| 552 | *Morus mesozygia Stapf* | Moraceae | Moraceae | GC | - | - | + | + | + | + |
| 553 | *Motandra guineensis A. DC.* | Apocynaceae | Apocynaceae | AT | - | + | - | + | + | + |
| 554 | *Mucuna poggei Taub. Var. occidentalis Hepper* | Papilionaceae | Fabaceae | AT | - | + | - | - | + | + |
| 555 | *Mucuna pruriens var. pruriens (Linn.) DC.* | Papilionaceae | Fabaceae | Pan | - | + | - | + | + | + |
| 556 | *Mucuna sloanei Fawc. & Rendle var.pruriens* | Papilionaceae | Fabaceae | GC | - | + | - | + | + | + |
| 557 | *Murdannia simplex (Vahl) Brenan* | Commelinaceae | Commelinaceae | Paleo | - | + | - | + | - | + |
| 558 | *Musanga cecropioides R. Br.* | Moraceae | Urticaceae | GC | + | - | + | - | + | + |
| 559 | *Musa paradisiaca Linn.* | Musaceae | Musaceae | I | - | - | - | - | - | + |
| 560 | *Musa sapientum Linn.* | Musaceae | Musaceae | I | - | + | - | + | + | + |
| 561 | *Mussaenda arcuata Lam.* | Rubiaceae | Rubiaceae | AT | - | - | - | - | - | + |
| 562 | *Mussaenda elegans Schum. & Thonn.* | Rubiaceae | Rubiaceae | GC | - | + | - | + | + | + |
| 563 | *Mussaenda erythrophylla Schum. & Thonn.* | Rubiaceae | Rubiaceae | GC | + | - | - | - | + | + |
| 564 | *Myrianthus arboreus P. Beauv.* | Moraceae | Urticaceae | GC | + | - | + | - | + | + |
| 565 | *Napoleonaea vogelii Hook. ex Planch.* | Lecytidaceae | Lecytidaceae | GC | + | + | + | + | + | + |
| 566 | *Nauclea diderrichii (de Wild. & Th. Dur.) Merril.* | Rubiaceae | Rubiaceae | GC | + | - | + | - | + | + |
| 567 | *Nauclea latifolia Sm. = Sarcocephalus latifolius* | Rubiaceae | Rubiaceae | AT | - | + | - | + | + | + |
| 568 | *Nauclea pobeguinii Pobeg. ex pellegr. = Sarcocephalus pobeguinii* | Rubiaceae | Rubiaceae | GC | + | - | + | + | - | + |
| 569 | *Nelsonia canescens (Lam.) Spreng.* | Acanthaceae | Acanthaceae | Pan | - | + | - | + | - | + |
| 570 | *Nephrolepis bisserata (Sw.) Schott.* | Nephrolepidaceae | Lomariopsidaceae | Paleo | + | + | - | + | + | + |
| 571 | *Nephrolepis undulata (Afzel.ex Sw.) J.Sm.* | Nephrolepidaceae | Lomariopsidaceae | Pan | - | - | - | + | + | + |
| 572 | *Nervilia kotschyi (Rchb.f.) Schiltr.* | Orchidaceae | Orchidaceae | AT | - | - | - | - | - | + |
| 573 | *Nervilia umbrosa (Rchb.f.)Schltr* | Orchidaceae | Orchidaceae | AT | - | + | - | - | + | + |
| 574 | *Nesogordonia papaverifolia (A.Chev.) R. Capuron* | Sterculiaceae | Malvaceae | GC | + | - | + | - | + | + |
| 575 | *Newbouldia laevis (P. Beauv.) Seemann. ex Bureau* | Bignoniaceae | Bignoniaceae | GC | + | + | - | + | + | + |
| 576 | *Ochna afzelii R. Br. ex Oliv.* | Ochnaceae | Ochnaceae | AT | - | + | - | + | + | + |
| 577 | *Ochna schweinfurtiana F. Hoff* | Ochnaceae | Ochnaceae | AT | - | + | - | - | - | + |
| 578 | *Octolobus angustatus Welw.ex benth. = O. spectabilis* | Sterculiaceae | Malvaceae | GC | + | - | - | - | + | + |
| 579 | *Olax subscorpioidea Oliv.= O. gambecola* | Olacaceae | Olacaceae | GC | + | + | - | + | + | + |
| 580 | *Olyra latifolia Linn.* | Gramineae | Gramineae | AA | + | + | - | + | + | + |
| 581 | *Oncoba spinosa Forsk.* | Flacourtiaceae | Salicaceae | AT | + | + | - | + | + | + |
| 582 | *Ophioglossum reticulatum Linn.* | Ophioglossaceae | Ophioglossaceae | AT | - | - | - | - | - | + |
| 583 | *Opilia amentacea Roxb.* | Opiliaceae | Opiliaceae | Pan | - | + | - | + | + | + |
| 584 | *Oplismenus burmanii (Retz.) P. Beauv.* | Gramineae | Gramineae | GC | + | + | - | + | + | + |
| 585 | *Oplismenus hirtellus (Retz.) P.Beauv.* | Gramineae | Gramineae | Paleo | - | + | - | + | + | + |
| 586 | *Ormocarpum sennoides (Willd.)DC.* | Papilionaceae | Fabaceae | GC | - | + | - | + | + | + |
| 587 | *Oxyanthus formosus Hook. f.ex Planc* | Rubiaceae | Rubiaceae | GC | + | + | - | - | + | + |
| 588 | *Oxyanthus racemosus ( Schum. & Thonn.)Keay* | Rubiaceae | Rubiaceae | GC | + | + | - | + | - | + |
| 589 | *Oxyanthus speciosus DC.= subpunctatus* | Rubiaceae | Rubiaceae | GC | + | + | - | - | + | + |
| 590 | *Oxyanthus unilocularis Hiern* | Rubiaceae | Rubiaceae | GC | - | + | + | + | + | + |
| 591 | *Pachystela brevipes (Bak) Baill.ex Engl. = Synsepalum brevipes* | Sapotaceae | Sapotaceae | GC | + | + | + | + | + | + |
| 592 | *Palisota barteri Hook.f.* | Commelinaceae | Commelinaceae | GC | + | - | - | - | + | + |
| 593 | *Palisota hirsuta (Thunb.) K. Schum.* | Commelinaceae | Commelinaceae | GC | + | - | - | + | + | + |
| 594 | *Pancovia pedicellaris Radlk.& Gilg= P. turbinata* | Sapindaceae | Sapindaceae | GC | + | - | - | - | + | + |
| 595 | *Pandanus candelabrum P.Beauv.* | Pandanaceae | Pandanaceae | GC | - | + | + | - | - | + |
| 596 | *Panda oleosa Pierre* | Pandaceae | Pandaceae | GC | + | - | + | - | + | + |
| 597 | *Panicum maximum Jacq.* | Gramineae | Gramineae | GC | - | + | - | + | + | + |
| 598 | *Pararistolochia goldieana (Hook.f.) Hutch. & Dalz.* | Aristolochiaceae | Aristolochiaceae | GC | - | + | - | - | + | + |
| 599 | *Parinari congensis F. Didr.* | Chrysobalanaceae | Chrysobalanaceae | GC | - | + | - | - | + | + |
| 600 | *Parinari excelsa Sabine* | Chrysobalanaceae | Chrysobalanaceae | AA | + | - | + | - | + | + |
| 601 | *Parinari glabra Oliv. = Maranthes glabra* | Chrysobalanaceae | Chrysobalanaceae | GC | + | - | + | - | + | + |
| 602 | *Parkia bicolor A.Cheval.* | Mimosaceae | Fabaceae | GC | + | - | + | - | + | + |
| 603 | *Parkia filicoidea Welw. Ex Oliv.* | Mimosaceae | Fabaceae | GC | - | - | + | - | + | + |
| 604 | *Parquetina nigrescens (Afzel.) Bullock.* | Asclepiadaceae | Apocynaceae | GC | - | + | - | + | + | + |
| 605 | *Paullinia pinnata Linn.* | Sapindaceae | Sapindaceae | AA | - | + | - | + | + | + |
| 606 | *Pauridiantha afzelii (Hiern.)Bremek.* | Rubiaceae | Rubiaceae | GC | + | - | - | - | + | + |
| 607 | *Pavetta corymbosa (DC.) F.N. Williams* | Rubiaceae | Rubiaceae | AT | + | + | - | + | + | + |
| 608 | *Pavetta genipifolia Schumach.* | Rubiaceae | Rubiaceae | GCW | - | - | - | - | + | + |
| 609 | *Pavetta lasioclada (K. Krause) Mildbr. ex Bremek.* | Rubiaceae | Rubiaceae | AT | - | - | - | + | + | + |
| 610 | *Pavetta mollissima Hutch.et Dalz* | Rubiaceae | Rubiaceae | GCW | - | - | - | + | - | + |
| 611 | *Peddiea fischeri Engl.* | Thymelaeaceae | Thymelaeaceae | GC | - | - | - | - | + | + |
| 612 | *Pellaea doniana Hook.* | Adiantaceae | Adiantaceae | GC | + | - | - | - | + | + |
| 613 | *Pennisetum purpureum Schumach.* | Gramineae | Gramineae | GC | - | - | - | + | + | + |
| 614 | *Pentadesma butyraccea Sabine* | Clusiaceae | Clusiaceae | AT | + | + | + | - | - | + |
| 615 | *Pentaclethra macrophylla Benth.* | Mimosaceae | Fabaceae | GC | + | - | + | - | - | + |
| 616 | *Pentodon pentandrus (Schum. & Thonn.) Vatke* | Rubiaceae | Rubiaceae | AT | - | + | - | + | - | + |
| 617 | *Pergularia daemia (Forssk.) Chiov.* | Asclepiadaceae | Apocynaceae | Paleo | - | + | - | + | + | + |
| 618 | *Persea americana Miller* | Lauraceae | Lauraceae | I | - | - | - | - | + | + |
| 619 | *Phaulopsis barteri (T. Anders.) Lindau* | Acanthaceae | Acanthaceae | AT | - | + | - | + | + | + |
| 620 | *Phaulopsis ciliata (Willd.) Hepper= P. falcisepala* | Acanthaceae | Acanthaceae | Paleo | + | + | - | + | + | + |
| 621 | *Philodendron spp* | Araceae | Araceae | Ind | - | - | - | - | - | + |
| 622 | *Phoenix reclinata Jacq.* | Arecaceae | Arecaceae | AT | - | + | - | + | - | + |
| 623 | *Phragmites karka (Retz.) Trin. ex Steud.* | Gramineae | Gramineae | Paleo | - | + | - | - | - | + |
| 624 | *Phragmanthera nigritana (Hook.f. ex Benth.) Balle* | Loranthaceae | Loranthaceae | GC | - | - | - | + | + | + |
| 625 | *Phyllanthus magnificens Brunel & Roux* | Euphorbiaceae | Phyllantaceae | GCW | - | - | - | + | + | + |
| 626 | *Phyllanthus muellerianus (O. Ktze.) Exell.* | Euphorbiaceae | Phyllantaceae | AT | + | + | - | - | + | + |
| 627 | *Phyllanthus reticulatus Poir.* | Euphorbiaceae | Phyllantaceae | AT | - | + | - | + | + | + |
| 628 | *Phymatodes scolopendria (Burm) Ching.* | Polypodiaceae | Polypodiaceae | Paleo | + | - | - | + | + | + |
| 629 | *Physalis angulata Linn.* | Solanaceae | Solanaceae | Pan | - | + | - | + | - | + |
| 630 | *Picralima nitida (Stapf) Th. et Hel.Durand* | Apocynaceae | Apocynaceae | GC | + | - | + | - | + | + |
| 631 | *Pierreodendron kerstingii (Engl.) Little* | Simaroubaceae | Simaroubaceae | GCW | - | + | - | + | - | + |
| 632 | *Piper arboreum Aublet* | Piperaceae | Piperaceae | Pan | - | - | - | - | + | + |
| 633 | *Piper guineense Schum. & Thonn.* | Piperaceae | Piperaceae | GC | + | - | - | + | + | + |
| 634 | *Piper nigrum Linn.* | Piperaceae | Piperaceae | I | - | - | - | - | - | + |
| 635 | *Piper umbellatum Linn.= Lepianthes umbellatum* | Piperaceae | Piperaceae | Pan | + | - | - | - | + | + |
| 636 | *Piptadeniastrum africanum (Hook. f.) Brenan* | Mimosaceae | Fabaceae | GC | + | - | + | - | + | + |
| 637 | *Pityrogramma calomelanos (Linn.) Link* | Adiantaceae | Adiantaceae | Pan | - | - | - | - | + | + |
| 638 | *Platostoma africanum P. Beauv.* | Labiatae | Lamiaceae | Paleo | - | + | - | + | + | + |
| 639 | *Platycerium angolense Welw.* | Polypodiaceae | Polypodiaceae | GC | + | + | - | + | + | + |
| 640 | *Platycerium stemaria (P. Beauv.) Desv.* | Polypodiaceae | Polypodiaceae | GC | + | - | - | - | + | + |
| 641 | *Pleiocarpa pycnantha (K. Schum.) Stapf.* | Apocynaceae | Apocynaceae | GC | - | - | - | + | - | + |
| 642 | *Polysphaeria arbuscula K. Schum.* | Rubiaceae | Rubiaceae | SZ | - | + | - | + | + | + |
| 643 | *Polystachya dolichophylla Schltr* | Orchidaceae | Orchidaceae | GC | - | - | - | - | - | + |
| 644 | *Polycoryne fernandensis (Hiern) Keay* | Rubiaceae | Rubiaceae | GC | - | - | - | + | + | + |
| 645 | *Polyscias fulva (Hiern) Harms* | Araliaceae | Araliaceae | GC | - | - | - | - | + | + |
| 646 | *Polystachya modesta Rchb.f.* | Orchidaceae | Orchidaceae | AT | - | - | - | - | - | + |
| 647 | *Pouchetia africana A. Rich.* | Rubiaceae | Rubiaceae | AT | - | - | - | - | - | + |
| 648 | *Pouchetia parviflora Benth.* | Rubiaceae | Rubiaceae | GC | - | - | - | + | - | + |
| 649 | *Pouzolzia guineensis Benth.* | Urticaceae | Urticaceae | AT | - | + | - | + | - | + |
| 650 | *Premna angolensis Gürke* | Verbenaceae | Lamiaceae | GC | - | + | - | + | + | + |
| 651 | *Premna hispida Benth.* | Verbenaceae | Lamiaceae | GCW | - | + | - | - | + | + |
| 652 | *Premna quadrifolia Schum. & Thonn.* | Verbenaceae | Lamiaceae | GC | - | - | - | + | + | + |
| 653 | *Protomagabaria stoptefana (Beille) Hutch.* | Euphorbiaceae | Euphorbiaceae | GC | + | - | - | - | - | + |
| 654 | *Pseudospondias microcarpa ( A. Rich.) Engl.* | Anacardiaceae | Anacardiaceae | AT | + | + | + | - | - | + |
| 655 | *Pseuderanthemum tunicatum (Afzel.)Milne-Redhead* | Acanthaceae | Acanthaceae | GC | - | - | - | - | + | + |
| 656 | *Psidium guajava Linn.* | Myrtaceae | Myrtaceae | I | - | - | - | + | + | + |
| 657 | *Psophocarpus palustris Desv.* | Papilionaceae | Fabaceae | AT | - | - | - | + | + | + |
| 658 | *Psychotria calva Hiern* | Rubiaceae | Rubiaceae | GC | - | + | - | + | + | + |
| 659 | *Psychotria elongato-sepala (Hiern.)Petit.* | Rubiaceae | Rubiaceae | GC | + | - | - | - | + | + |
| 660 | *Psychotria latistipula Benth.* | Rubiaceae | Rubiaceae | GCE | - | + | - | - | + | + |
| 661 | *Psychotria linderi Hepper* | Rubiaceae | Rubiaceae | GCW | - | - | - | + | - | + |
| 662 | *Psychotria peduncularis (Salisb.) Steyerm.* | Rubiaceae | Rubiaceae | GC | + | - | - | + | + | + |
| 663 | *Psychotria psychotrioides (DC.) Roberty* | Rubiaceae | Rubiaceae | AT | - | + | - | + | + | + |
| 664 | *Psychotria vogeliana Benth.* | Rubiaceae | Rubiaceae | AT | + | + | - | + | + | + |
| 665 | *Pteridium aquilinum (Linn.) Kunth* | Dennstaedtiaceae | Dennstaedtiaceae | Cosm | - | - | - | - | + | + |
| 666 | *Pteris atrovirens Willd.* | Adiantaceae | Adiantaceae | GC | + | - | - | - | + | + |
| 667 | *Pteris burtonii Back.* | Adiantaceae | Adiantaceae | GC | + | - | - | - | + | + |
| 668 | *Pterygota macrocarpa K. Schum.* | Sterculiaceae | Malvaceae | GC | + | - | + | - | + | + |
| 669 | *Pterocarpus mildbraedii Harms* | Papilionaceae | Fabaceae | GC | - | - | - | - | + | + |
| 670 | *Pterocarpus santalinoides Linn. Herit.ex DC.* | Papilionaceae | Fabaceae | AA | + | + | - | + | - | + |
| 671 | *Pteris togoensis Hieron.* | Adiantaceae | Adiantaceae | AT | - | - | - | - | + | + |
| 672 | *Pueraria phaseiloides (Roxb.) Benth.* | Papilionaceae | Fabaceae | I | - | + | - | + | - | + |
| 673 | *Pycnanthus angolensis (Welw.) Warb.* | Myristicaceae | Myristicaceae | GC | + | - | + | + | + | + |
| 674 | *Pyrenacantha vogeliana Baill.* | Icacinaceae | Icacinaceae | GC | + | - | - | + | + | + |
| 675 | *Rangaeris rhipsololisocia (Rchb. f.) Summerh.* | Orchidaceae | Orchidaceae | GC | - | - | - | - | + | + |
| 676 | *Raphia hookeri Mann & Wendl= R. sassandrensis* | Arecaceae | Arecaceae | GC | + | - | - | - | - | + |
| 677 | *Raphidiocystis mannii Hook.f.* | Cucurbitaceae | Cucurbitaceae | GC | - | - | - | - | + | + |
| 678 | *Raphia sudanica A.Chev.* | Arecaceae | Arecaceae | SZ | - | + | - | - | - | + |
| 679 | *Rauvolfia caffra Sond.* | Apocynaceae | Apocynaceae | AT | - | - | + | - | + | + |
| 680 | *Rauvolfia vomitoria Afzel.* | Apocynaceae | Apocynaceae | AT | + | + | + | + | + | + |
| 681 | *Rhaphidophora africana N. E. Br.* | Araceae | Araceae | GC | + | - | - | - | + | + |
| 682 | *Rhaphiostylis beninensis (Hook.f. ex Planch) Planch. ex Benth.* | Icacinaceae | Icacinaceae | GC | + | + | - | + | + | + |
| 683 | *Rhigiocarya racemifera Miers.* | Menispermaceae | Menispermaceae | GC | + | - | - | + | + | + |
| 684 | *Rhinacanthus virens (Nees) Milne-Redhead var.virens* | Acanthaceae | Acanthaceae | GC | - | - | - | - | + | + |
| 685 | *Rhynchosia densiflora (Roth) DC.* | Papilionaceae | Fabaceae | Paleo | - | + | - | + | + | + |
| 686 | *Rhynchosia pycnostachya (DC.) Meikle* | Papilionaceae | Fabaceae | AT | - | - | - | + | + | + |
| 687 | *Ricinus communis Linn.* | Euphorbiaceae | Euphorbiaceae | Pan | - | + | - | + | - | + |
| 688 | *Ricinodendron heudelotii (Baill.) Pierre ex Pax* | Euphorbiaceae | Euphorbiaceae | GC | + | - | + | + | + | + |
| 689 | *Rinorea brachypetala (Turcz.) O. Ktze* | Violaceae | Violaceae | GC | - | - | - | - | - | + |
| 690 | *Rinorea illicifolia (Welw.ex Oliv.)O.Ktze* | Violaceae | Violaceae | AM | + | - | - | - | - | + |
| 691 | *Rinorea longicuspis Engl. =welwitschii* | Violaceae | Violaceae | GC | + | - | + | - | - | + |
| 692 | *Rinorea yaundensis Engl.* | Violaceae | Violaceae | GC | - | - | - | - | - | + |
| 693 | *Ritchiea capporoides (Andr.)Britten* | Capparaceae | Capparaceae | AT | + | + | - | + | - | + |
| 694 | *Ritchiea reflexa (Thonn.)Gild&benedict.* | Capparaceae | Capparaceae | AT | + | - | - | + | - | + |
| 695 | *Rothmannia longiflora Salisb.* | Rubiaceae | Rubiaceae | GC | + | - | - | + | + | + |
| 696 | *Rothmannia urcelliformis (Hiern) Bullock.ex Robyns* | Rubiaceae | Rubiaceae | AT | - | + | - | + | + | + |
| 697 | *Rothmannia whitfildi (Lindl.)Dandy* | Rubiaceae | Rubiaceae | GC | + | - | - | - | + | + |
| 698 | *Rottboellia aristata Linn.f* | Gramineae | Gramineae | AT | - | - | - | - | - | + |
| 699 | *Rubus pinnatus Willd.* | Rosaceae | Rosaceae | AM | - | - | - | - | + | + |
| 700 | *Ruthalicia eglandulosa (Hook.f.) C. Jeffrey* | Cucurbitaceae | Cucurbitaceae | GC | + | - | - | - | + | + |
| 701 | *Rutidea parviflora DC.* | Rubiaceae | Rubiaceae | AT | + | - | - | - | + | + |
| 702 | *Rytigynia canthioides (Benth.) Robyns* | Rubiaceae | Rubiaceae | GC | + | + | - | - | + | + |
| 703 | *Rytigynia umbellulata (Hiern) Robyns* | Rubiaceae | Rubiaceae | GC | + | + | - | + | + | + |
| 704 | *Saba comorensis (Boj.) Pichon* | Apocynaceae | Apocynaceae | AM | - | + | - | - | + | + |
| 705 | *Saba senegalensis (A.DC.) Pichon* | Apocynaceae | Apocynaceae | SZ | - | - | - | - | + | + |
| 706 | *Saba thompsonii (A.Chev.)Pichon.* | Apocynaceae | Apocynaceae | AT | - | + | - | - | + | + |
| 707 | *Sabicea calycina Benth.* | Rubiaceae | Rubiaceae | GC | + | + | - | + | + | + |
| 708 | *Sabicea speciosa K. Schum.* | Rubiaceae | Rubiaceae | GC | - | - | - | - | + | + |
| 709 | *Saccharum officinarum Linn.* | Gramineae | Gramineae | I | - | - | - | + | - | + |
| 710 | *Sacosperma paniculatum (Benth.)G.Tayl* | Rubiaceae | Rubiaceae | GC | - | - | - | - | + | + |
| 711 | *Salacia chlorantha Oliv.* | Celastraceae | Celastraceae | GC | - | - | - | - | - | + |
| 712 | *Salacia erecta (G. Don) Walp.* | Celastraceae | Celastraceae | GC | + | - | - | - | + | + |
| 713 | *Salacia leptoclada Tul. = S. baumannii* | Celastraceae | Celastraceae | GC | + | + | - | + | + | + |
| 714 | *Salacia pallescens Oliv.* | Celastraceae | Celastraceae | GC | - | + | - | + | + | + |
| 715 | *Salacia togoica Loes.* | Celastraceae | Celastraceae | GC | - | - | - | + | - | + |
| 716 | *Sansevieria liberica Gér.& Labr.* | Agavaceae | Asparagaceae | AT | - | + | - | + | + | + |
| 717 | *Sapium ellipticum (Hochst) Pax* | Euphorbiaceae | Euphorbiaceae | AT | - | + | - | - | + | + |
| 718 | *Sarcophrynium brachystachyum (Benth.) K.Schum.* | Marantaceae | Marantaceae | GC | + | - | - | - | + | + |
| 719 | *Scadoxus multiflorus (Martyn.) Rafin. =Haementus multiflorus* | Amaryllidaceae | Amaryllidaceae | AT | + | + | - | + | - | + |
| 720 | *Scleria boivinii Steud.* | Cyperaceae | Cyperaceae | GC | + | - | - | - | + | + |
| 721 | *Scleria lagoensis Boeck* | Cyperaceae | Cyperaceae | Pan | - | - | - | - | - | + |
| 722 | *Sebaea pumila (Bak.) Schinz* | Gentianaceae | Gentianaceae | GC | - | - | - | - | - | + |
| 723 | *Secamone afzelii (Schultes) K. Schum.* | Asclepiadaceae | Apocynaceae | GC | + | + | - | + | + | + |
| 724 | *Securinega virosa (Roxb. ex Willd) Baill = Flueggea virosa* | Euphorbiaceae | Phyllantaceae | Paleo | - | + | - | + | + | + |
| 725 | *Selaginella kalbreyeri Bak.* | Selaginellaceae | Selaginellaceae | GC | - | - | - | - | + | + |
| 726 | *Selaginella myosorus (Sm.) Alston* | Selaginellaceae | Selaginellaceae | GC | + | - | - | - | + | + |
| 727 | *Selaginella versicolor Spring* | Selaginellaceae | Selaginellaceae | GC | - | - | - | - | + | + |
| 728 | *Senecio biafrae Oliv. & Hiern.* | Asteraceae | Asteraceae | GC | - | - | - | + | - | + |
| 729 | *Sericanthe chevalieri (K. Krause) Robbrecht var Chevaleri* | Rubiaceae | Rubiaceae | AT | - | + | - | - | + | + |
| 730 | *Setaria barbata (Lam.) Kunth.* | Gramineae | Gramineae | Pan | - | + | - | + | + | + |
| 731 | *Setaria megaphylla (Steud.)Dur. & Schinz.* | Gramineae | Gramineae | GC | - | + | - | + | + | + |
| 732 | *Sherbournia bignoniiflora (Welw.) Hua.* | Rubiaceae | Rubiaceae | GC | + | - | - | + | + | + |
| 733 | *Sherbournia calycina (G. Don.) Hua* | Rubiaceae | Rubiaceae | GCW | - | - | - | + | + | + |
| 734 | *Sida acuta Burm. f.* | Malvaceae | Malvaceae | Pan | - | + | - | - | + | + |
| 735 | *Sida rhombifolia var. beta Linn.* | Malvaceae | Malvaceae | Pan | - | + | - | + | + | + |
| 736 | *Smeathmannia laevigata Soland. ex R. Br.* | Passifloraceae | Passifloraceae | AT | - | - | - | - | + | + |
| 737 | *Smilax kraussiana Meisn.* | Liliaceae | Similacaceae | AM | - | + | - | + | + | + |
| 738 | *Solanum verbascifolium Linn.* | Solanaceae | Solanaceae | Pan | - | + | - | - | - | + |
| 739 | *Solenostemon monostachyus (P.Beauv.) var. monostachyus* | Labiatae | Lamiaceae | AT | - | + | - | + | - | + |
| 740 | *Sorghum arundinaceum (Desv.) Stapf.* | Gramineae | Gramineae | AT | - | + | - | + | + | + |
| 741 | *Sorindeia grandifolia Engel. = S. warneckei* | Anacardiaceae | Anacardiaceae | GC | - | + | - | - | + | + |
| 742 | *Sorindeia juglandifolia (A.Rich.)Planch.ex Oliv.* | Anacardiaceae | Anacardiaceae | AT | - | - | + | - | + | + |
| 743 | *Spathodea campanulata P. Beauv.* | Bignoniaceae | Bignoniaceae | GC | + | + | + | + | + | + |
| 744 | *Spondias monbin Linn.* | Anacardiaceae | Anacardiaceae | Pan | - | + | - | - | + | + |
| 745 | *Spondiathus preussii Engl.* | Euphorbiaceae | Phyllantaceae | GC | + | - | - | - | - | + |
| 746 | *Sporobolus pyramidalis P.Beauv.* | Gramineae | Gramineae | AT | - | + | - | - | - | + |
| 747 | *Stachyanthus occidentalis (Keay & miège) Boutique* | Icacinaceae | Icacinaceae | GC | - | + | - | + | + | + |
| 748 | *Stephania dinklagei (Engl.) Diels* | Menispermaceae | Menispermaceae | GC | - | - | - | - | + | + |
| 749 | *Stereospermum acuminatissimum K. Schum.* | Bignoniaceae | Bignoniaceae | GC | + | + | - | + | + | + |
| 750 | *Sterculia foetida Linn.* | Sterculiaceae | Malvaceae | AT | - | - | - | - | - | + |
| 751 | *Sterculia oblonga Mast.* | Sterculiaceae | Malvaceae | GC | + | - | + | - | + | + |
| 752 | *Sterculia rhinopetala K. Schum.* | Sterculiaceae | Malvaceae | GC | - | - | - | - | + | + |
| 753 | *Sterculia tragacantha Lindl.* | Sterculiaceae | Malvaceae | AT | + | + | + | + | + | + |
| 754 | *Stictocardia beraviensis (Vatke) Hallier f.* | Convolvulaceae | Convolvulaceae | AM | - | - | - | + | - | + |
| 755 | *Streptogyna crinita P. Beauv.* | Gramineae | Gramineae | GC | + | + | - | - | + | + |
| 756 | *Strombosia glauscescens J.Leonard var. lucida =S. pustulata* | Olacaceae | Strombosiaceae | GC | + | - | + | - | + | + |
| 757 | *Strophanthus hispidus DC.* | Apocynaceae | Apocynaceae | AT | + | + | - | + | + | + |
| 758 | *Strophanthus sarmentosus DC.* | Apocynaceae | Apocynaceae | AT | + | + | - | + | + | + |
| 759 | *Struchium sparganophora ( Linn.)O. Ktze.* | Asteraceae | Asteraceae | Pan | - | - | - | + | + | + |
| 760 | *Strychnos afzelii Gilg* | Loganiaceae | Loganiaceae | GC | + | - | - | + | + | + |
| 761 | *Strychnos barteri Solered.* | Loganiaceae | Loganiaceae | GC | + | + | - | + | + | + |
| 762 | *Strychnos floribunda Gilg* | Loganiaceae | Loganiaceae | GC | + | + | - | + | + | + |
| 763 | *Strychnos johnsonii Hutch.* | Loganiaceae | Loganiaceae | GC | - | - | - | - | + | + |
| 764 | *Strychnos nigritana Bak.* | Loganiaceae | Loganiaceae | GC | - | + | - | + | - | + |
| 765 | *Strychnos soubrensis Hutch.* | Loganiaceae | Loganiaceae | GC | + | - | - | + | + | + |
| 766 | *Strychnos usambarensis Gilg.* | Loganiaceae | Loganiaceae | GC | + | + | - | + | - | + |
| 767 | *Stylochiton hostifolius Engl.* | Araceae | Araceae | SZ | - | + | - | - | + | + |
| 768 | *Symphonia globulifera Linn. f.* | Clusiaceae | Clusiaceae | AA | + | - | + | - | - | + |
| 769 | *Synaptolepis retusa H. W. pearson* | Tiliaceae | Malvaceae | GC | - | - | - | - | - | + |
| 770 | *Synedrella nodiflora Gaertn.* | Asteraceae | Asteraceae | Pan | - | + | - | + | + | + |
| 771 | *Syzygium guineense (Willd.) DC. Subsp. Guineense* | Myrtaceae | Myrtaceae | SZ | - | + | - | - | - | + |
| 772 | *Syzygium ovariense ( P.Beauv.)Benth.* | Myrtaceae | Myrtaceae | GC | + | - | - | - | - | + |
| 773 | *Tabernaemontana pachysiphon Stapf. Var. cumminsii* | Apocynaceae | Apocynaceae | GCW | - | - | - | - | + | + |
| 774 | *Tacazzea apiculata Oliv.* | Asclepiadaceae | Apocynaceae | AT | - | + | - | + | + | + |
| 775 | *Tarenna pavettoides (Harv.) Sim ssp. guineensis Degreef* | Rubiaceae | Rubiaceae | GC | - | - | - | - | + | + |
| 776 | *Tectaria fernandensis (Bak.) C.Chr.* | Aspidiaceae | Dryopteridaceae | GC | - | - | - | - | + | + |
| 777 | *Tectona grandis Linn. f.* | Verbenaceae | Verbenaceae | I | - | + | - | + | - | + |
| 778 | *Telosma africanum (N. E. Br.) Colville* | Asclepiadaceae | Apocynaceae | GC | - | - | - | + | + | + |
| 779 | *Tephrosia linearis (Willd.)Pers* | Papilionaceae | Fabaceae | AM | - | - | - | - | - | + |
| 780 | *Teramnus labialis (Linn. f.) Spreng.* | Papilionaceae | Fabaceae | AT | - | - | - | + | - | + |
| 781 | *Terminalia glaucescens Planch.ex Benth.* | Combretaceae | Combretaceae | SZ | - | + | - | + | + | + |
| 782 | *Terminalia superba Engl. & Diels* | Combretaceae | Combretaceae | GC | + | + | + | + | + | + |
| 783 | *Tetracera affinis Hutch.* | Dilleniaceae | Dilleniaceae | GCW | + | - | - | - | - | + |
| 784 | *Tetracera alnifolia Willd.* | Dilleniaceae | Dilleniaceae | AT | + | + | - | + | + | + |
| 785 | *Tetrorchidium didymostemon (Baill.) Pax & Hoffm.* | Euphorbiaceae | Euphorbiaceae | GC | + | - | + | - | + | + |
| 786 | *Tetracera stuhlmanniana Gilg.= T. potatoria* | Dilleniaceae | Dilleniaceae | GC | + | - | - | - | + | + |
| 787 | *Tetrapleura tetraptera (Schum.& Thonn.) Taub.* | Mimosaceae | Fabaceae | GC | - | + | + | + | + | + |
| 788 | *Thalia welwitchii Ridl.= T. geniculata L.* | Marantaceae | Marantaceae | AT | + | - | - | - | + | + |
| 789 | *Thaumatococus danielii (Benn.) Benth.* | Marantaceae | Marantaceae | GC | + | - | - | - | + | + |
| 790 | *Theobroma cacao Linn.* | Sterculiaceae | Malvaceae | I | - | - | - | + | + | + |
| 791 | *Thonningia sanguinea Vahl* | Balanophoraceae | Balanophoraceae | GC | + | + | - | + | + | + |
| 792 | *Thunbergia chrysops Hook.* | Acanthaceae | Acanthaceae | GC | - | - | - | - | + | + |
| 793 | *Thunbergia cynanchyfolia Benth.* | Acanthaceae | Acanthaceae | GC | - | + | - | + | + | + |
| 794 | *Thunbergia fasciculata Lindeau* | Acanthaceae | Acanthaceae | AT | - | - | - | - | - | + |
| 795 | *Tiliacora leonensis (Sc. Elliot)Diels* | Menispermaceae | Menispermaceae | GCW | + | - | - | - | - | + |
| 796 | *Tragia benthamii Bak.* | Euphorbiaceae | Euphorbiaceae | GC | - | - | - | + | + | + |
| 797 | *Tragia mildbraediana Px & Hoffm.* | Euphorbiaceae | Euphorbiaceae | GC | - | - | - | - | + | + |
| 798 | *Treculia africana Decne* | Moraceae | Moraceae | GC | + | - | + | + | + | + |
| 799 | *Trema orientalis (Linn.) Bl.* | Ulmaceae | Canabaceae | Paleo | - | + | + | + | + | + |
| 800 | *Tricalysia chevaleri K. Krause* | Rubiaceae | Rubiaceae | GC | - | - | - | - | + | + |
| 801 | *Tricalysia coriacea Hiern.* | Rubiaceae | Rubiaceae | GC | - | - | + | - | - | + |
| 802 | *Triclisia dictyophylla Diels* | Menispermaceae | Menispermaceae | GC | + | - | - | - | + | + |
| 803 | *Trichilia heudelotii Planch. ex Oliv. = T. monadelpha* | Meliaceae | Meliaceae | GC | + | - | - | - | + | + |
| 804 | *Trichilia megalantha Harms* | Meliaceae | Meliaceae | GC | - | - | - | - | - | + |
| 805 | *Tricalysia okelensis Hiern var. okelensis* | Rubiaceae | Rubiaceae | SZ | - | + | - | - | + | + |
| 806 | *Trichilia prieureana A. Juss.subsp. prieuriana* | Meliaceae | Meliaceae | GC | + | + | + | + | + | + |
| 807 | *Tricalysia reflexa Hutch.= Pleiocarpa mutica* | Rubiaceae | Rubiaceae | GC | + | - | - | - | + | + |
| 808 | *Tricalysia reticulata (Benth.)Hiern* | Rubiaceae | Rubiaceae | GC | - | + | - | - | - | + |
| 809 | *Triclisia subcordata Oliv.* | Menispermaceae | Menispermaceae | GC | - | + | - | + | + | + |
| 810 | *Trilepisium madagascariense DC.* | Moraceae | Moraceae | AM | - | + | + | - | + | + |
| 811 | *Triplochiton scleroxylon K.Schum.* | Sterculiaceae | Malvaceae | GC | + | - | + | + | + | + |
| 812 | *Triumfetta cordifolia A. Rich.* | Tiliaceae | Malvaceae | Pan | - | + | - | + | + | + |
| 813 | *Triumfetta rhomboidea Jacq.* | Tiliaceae | Malvaceae | Pan | + | + | - | + | - | + |
| 814 | *Trydactyle bicaudata (Lindl.) Schltr.* | Orchidaceae | Orchidaceae | GC | - | - | - | - | - | + |
| 815 | *Turraeanthus africana (Welw. ex C.DC.) Pellegr.* | Meliaceae | Meliaceae | GC | - | - | + | - | + | + |
| 816 | *Turraea heterophylla Sm.* | Meliaceae | Meliaceae | AT | - | - | - | + | - | + |
| 817 | *Tylophora conspicua N.E.Br.* | Asclepiadaceae | Apocynaceae | GC | - | - | - | + | + | + |
| 818 | *Tylophora dahomensis K. Schum.* | Asclepiadaceae | Apocynaceae | GCW | - | + | - | + | - | + |
| 819 | *Tylophora glauca Buliock* | Asclepiadaceae | Apocynaceae | GC | - | - | - | + | + | + |
| 820 | *Tylophora oculata N. E. Br.* | Asclepiadaceae | Apocynaceae | GC | + | - | - | + | - | + |
| 821 | *Tylophora sylvatica Decne* | Asclepiadaceae | Apocynaceae | GC | - | + | - | + | + | + |
| 822 | *Uapaca esculentus A. Chev.* | Euphorbiaceae | Phyllantaceae | GC | + | - | - | - | - | + |
| 823 | *Uapaca guineensis Müll. Arg.* | Euphorbiaceae | Phyllantaceae | GC | + | - | + | - | - | + |
| 824 | *Uapaca heudelotii Baill.* | Euphorbiaceae | Phyllantaceae | AT | + | + | + | + | - | + |
| 825 | *Uapaca paludosa Aubrév. et Léandri* | Euphorbiaceae | Phyllantaceae | GC | + | - | - | - | + | + |
| 826 | *Uncaria africana G.Don.* | Rubiaceae | Rubiaceae | GC | + | - | - | - | + | + |
| 827 | *Uncaria talbotii Wernham* | Rubiaceae | Rubiaceae | GC | + | - | - | - | + | + |
| 828 | *Urena lobata Linn.* | Malvaceae | Malvaceae | Pan | + | + | - | + | - | + |
| 829 | *Urera oblongifolia Benth.* | Urticaceae | Urticaceae | GCW | + | - | - | - | + | + |
| 830 | *Urera obovata Benth.* | Urticaceae | Urticaceae | GCW | + | - | - | + | + | + |
| 831 | *Usteria guineensis Willd.* | Loganiaceae | Loganiaceae | AT | - | + | - | - | + | + |
| 832 | *Uvaria chamae P. Beauv.* | Annonaceae | Annonaceae | AT | - | + | - | + | + | + |
| 833 | *Uvaria doeringii Diels* | Annonaceae | Annonaceae | GCW | - | - | - | + | + | + |
| 834 | *Uvaria ovata subsp. Ovata (Dunal. ) A. DC. subsp.ovata* | Annonaceae | Annonaceae | AT | - | + | - | + | + | + |
| 835 | *Uvaria sofa Sc. Elliot* | Annonaceae | Annonaceae | AT | - | - | - | + | - | + |
| 836 | *Vahadenia caillei (A.Chev.) Stapf ex Hutch* | Apocynaceae | Apocynaceae | GCW | - | - | - | - | - | + |
| 837 | *Vangueriopsis discolor (Benth.)Verdc.* | Rubiaceae | Rubiaceae | GCW | - | - | - | - | - | + |
| 838 | *Vangueriopsis nigerica Robyns* | Rubiaceae | Rubiaceae | SZ | - | - | - | - | + | + |
| 839 | *Vangueriopsis spinosa (Schum.& Thonn.) Hepper* | Rubiaceae | Rubiaceae | AT | - | + | - | + | + | + |
| 840 | *Ventilago diffusa ( G. Don.) Exell = V. africana* | Rhamnaceae | Rhamnaceae | GC | + | - | - | + | + | + |
| 841 | *Vernonia amygdalina Del.* | Asteraceae | Asteraceae | AT | - | - | - | - | + | + |
| 842 | *Vernonia cinerea (Linn.) Less.* | Asteraceae | Asteraceae | Pan | - | - | - | + | + | + |
| 843 | *Vernonia colorata (Willd.) Drake* | Asteraceae | Asteraceae | AT | - | + | - | + | + | + |
| 844 | *Vernonia conferta Benth.* | Asteraceae | Asteraceae | GC | + | - | + | - | - | + |
| 845 | *Vigna vexillata (Linn.) A.Rich.* | Papilionaceae | Fabaceae | Pan | - | + | - | + | - | + |
| 846 | *Vincentella passargei (Engl.)Aubrév.= Synsepalum passargei* | Sapotaceae | Sapotaceae | SZ | - | + | - | - | - | + |
| 847 | *Vismia guineensis (Linn.)Choisy* | Clusiaceae | Hypericaceae | GC | - | - | - | - | - | + |
| 848 | *Vitex doniana Sweet* | Verbenaceae | Lamiaceae | AT | - | + | + | + | + | + |
| 849 | *Vitex ferruginea Schum & Thonn.* | Verbenaceae | Lamiaceae | GC | - | - | + | - | + | + |
| 850 | *Vitex oxicuspis Bak.* | Verbenaceae | Lamiaceae | GC | + | - | - | - | - | + |
| 851 | *Vitex rivularis Gürke* | Verbenaceae | Lamiaceae | GC | + | - | - | - | - | + |
| 852 | *Vitex thyrsiflora Bak.* | Verbenaceae | Lamiaceae | GC | - | - | - | - | - | + |
| 853 | *Vittaria guinensis Desv.* | Vittariaceae | Pteridaceae | GC | - | - | - | - | - | + |
| 854 | *Voacanga africana Stapf. = V. thouarsii* | Apocynaceae | Apocynaceae | GC | + | + | + | + | + | + |
| 855 | *Waltheria indica Linn.* | Sterculiaceae | Malvaceae | Pan | - | - | - | + | - | + |
| 856 | *Warneckea cinnamomoides (Benth.) Exell.* | Melastomataceae | Melastomataceae | GCW | + | - | - | + | - | + |
| 857 | *Warneckea membranifolia (Hook. f.) Jacq.-Fél.-* | Melastomataceae | Melastomataceae | GC | - | - | - | - | + | + |
| 858 | *Whitfieldia elongata (P.Beauv.)De Wild.et Th.Dur* | Acanthaceae | Acanthaceae | GCE | - | - | - | - | + | + |
| 859 | *Wissadula rostrata (Schum. & Thonn.) Hook. f.* | Malvaceae | Malvaceae | AT | - | - | - | + | + | + |
| 860 | *Xanthosoma mafafa Schott* | Araceae | Araceae | I | - | - | - | + | + | + |
| 861 | *Ximenia americana Linn.* | Olacaceae | Ximeniaceae | Pan | - | + | - | + | + | + |
| 862 | *Xylopia aethiopica (Dural) A. Rich.* | Annonaceae | Annonaceae | AT | + | + | + | - | + | + |
| 863 | *Xylopia parviflora (A.Rich.) Benth.* | Annonaceae | Annonaceae | AT | + | + | + | + | + | + |
| 864 | *Xylopiastrum taiense Aubré.= X. acutiflora* | Annonaceae | Annonaceae | GC | + | - | - | - | - | + |
| 865 | *Xylopia villosa Chipp* | Annonaceae | Annonaceae | GC | + | - | - | - | + | + |
| 866 | *Zanha golungensis Hiern* | Sapindaceae | Sapindaceae | AT | - | + | - | - | + | + |
| 867 | *Zehneria hallii C. Jeffrey* | Cucurbitaceae | Cucurbitaceae | AT | - | + | - | - | - | + |
| 868 | *Zehneria minutiflora (Cogn.)Jeffey* | Cucurbitaceae | Cucurbitaceae | AT | - | - | - | - | - | + |
